# Polymerizing Like Mussels Do: Toward Synthetic Mussel Foot Proteins and Resistant Glues

**DOI:** 10.1002/anie.201809587

**Published:** 2018-10-31

**Authors:** Justus Horsch, Patrick Wilke, Matthias Pretzler, Maximilian Seuss, Inga Melnyk, Dario Remmler, Andreas Fery, Annette Rompel, Hans G. Börner

**Affiliations:** ^1^ Laboratory for Organic Synthesis of Functional Systems Department of Chemistry Humboldt-Universität zu Berlin Brook-Taylor-Straße 2 12489 Berlin Germany; ^2^ Universität Wien Fakultät für Chemie Institut für Biophysikalische Chemie Althanstraße 14 1090 Wien Austria; ^3^ Leibniz-Institut für Polymerforschung Dresden e.V. Institute of Physical Chemistry and Polymer Physics Hohe Straße 6 01069 Dresden Germany; ^4^ Technische Universität Dresden Chair of Physical Chemistry of Polymeric Materials Hohe Straße 6 01069 Dresden Germany

**Keywords:** adhesives, enzyme-induced polymerization, mussel glue, synthetic protein mimics, tyrosinase activation

## Abstract

A novel strategy to generate adhesive protein analogues by enzyme‐induced polymerization of peptides is reported. Peptide polymerization relies on tyrosinase oxidation of tyrosine residues to Dopaquinones, which rapidly form cysteinyldopa‐moieties with free thiols from cysteine residues, thereby linking unimers and generating adhesive polymers. The resulting artificial protein analogues show strong adsorption to different surfaces, even resisting hypersaline conditions. Remarkable adhesion energies of up to 10.9 mJ m^−2^ are found in single adhesion events and average values are superior to those reported for mussel foot proteins that constitute the gluing interfaces.

For decades, mussel glues have offered inspiration for a variety of bio‐mimetic materials.^1^ Progress in understanding the mechanisms of the concerted self‐assembly process of mussel foot proteins (mfps) that constitute the adhesion system led to the recognition of l‐3,4‐dihydroxyphenylalanine (Dopa) as a key moiety for adhesion.^2^ This triggered a rich class of Dopa‐carrying polymers with remarkable property profiles.^3^ Dopa could either be in‐built directly into polymers, leading to instant adhesives or be enzymatically generated on demand by tyrosinase processing of tyrosine‐bearing precursor polymers.^4^ Besides the contribution of Dopa to adhesion,^5^ the role for cohesion is increasingly recognized.^6^ The latter occurs either non‐covalently by Fe^3+^ cross‐linking of Dopa,^7^ or via a set of reaction mechanisms leading to covalent Dopa cross‐linking.^8^ Among those is the tyrosinase‐induced Michael‐type addition that constitutes an exploitable pathway for the establishment of a novel polymerization process. During formation of byssal threads, polyphenol oxidase (PPO) converts l‐Dopa residues in mfps into l‐Dopaquinone^9^ in a process that is referred to as quinone tanning. Cross‐linking occurs by nucleophilic addition of protein side chain functionalities^10^ and one of the most effective pathways involves cysteine addition to l‐Dopaquinones.^11^ Resulting cysteinyldopa cross‐links are observed in various adhesive protein systems such as Pcfp‐1 of *Perna canaliculus* and Mcfp‐6 footprints of *Mytilus californianus*.^12^

Herein we present our study on abstracting the cysteine–dopaquinone addition from mussel adhesive systems to polymerize peptides (unimers) via tyrosinase activation. The enzyme‐activated polyaddition exploits the formation of cysteinyldopa connectivities and leads to synthetic adhesive protein analogues.

The mfp‐1 family constitutes a tough and flexible coating of the byssal threads. In *Mytilus edulis*, the Mefp‐1 sequence contains a highly repetitive decamer AKPSYPPTYK (Figure 1).^13^ Hence, polymerization of this consensus sequence might result in a polymer that resembles Mefp‐1 in some aspects. To create a unimer capable of cysteinyldopa polymerization, AKPSYPPTYK was extended *C*‐terminally with Cys via a tri‐glycine spacer, resulting in AKPS**Y5**PPT**Y9**KGGGC (U_2_^C^). Owing to the presence of two tyrosines, U_2_^C^ would produce branched or cross‐linked polymers. For ease of analysis, a linear polymer was anticipated by polymerizing a unimer that contains only Tyr9 as Tyr5 was replaced by Ser5 (AKPS**S5**PPT**Y9**KGGGC, U_1_^C^).

**Figure 1:**
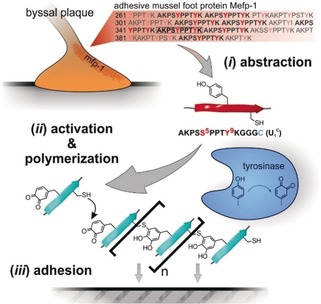
Principle of mussel‐inspired polymerization. *i*) An amino acid sequence is abstracted from Mefp‐1 and extension with Cys leads to the unimer U_1_^C^. *ii*) Polymerization of U_1_^C^ can be triggered by enzymatic oxidation of tyrosine to Dopaquinone, which reacts with thiol moieties of Cys residues, enabling the formation of cysteinyldopa linkages. *iii*) The obtained mfp analogues exhibit strong adhesion to various substrates, making them useful for coatings or glues.

Tyrosinase occurs almost ubiquitously throughout nature, is commercially available at low cost, and meets requirements for materials science applications.^14^ To avoid batch‐to‐batch variations,^15^ the enzymatic reactions were carried out with the active form of a recombinantly expressed tyrosinase (*Agaricus bisporus* polyphenol oxidase isoform 4, *Ab*PPO4).^14^, ^16^ The *Ab*PPO4 belongs to the PPO family from mushrooms and proved to practically instantaneously oxidize the tyrosine residues of U_1_^C^ and U_2_^C^ to Dopa and Dopaquinone as confirmed by MALDI‐TOF‐MS indicating species with +16 Da and +14 Da mass differences compared to non‐activated unimers (Supporting Information, Figures S1, S3). More importantly, mass spectrometry as a capable tool for polymer characterization^17^ proves the rapid formation of polymerization products. MALDI‐TOF‐MS shows species reaching up to about 30 kDa for polyU_1_^C^ and about 25 kDa for polyU_2_^C^, which could be assigned to a degree of polymerization (DP) of up to 21 (Figure 2 b). GPC analysis of polyU_1_^C^ revealed the formation of low‐ and high‐molecular weight fractions with apparent peak molecular weights of M_P,app._≈20 kDa and M_P,app._≈530 kDa, respectively. The fractions are already present after 5–10 min and correspond to DP_p,app._≈15 and 260, respectively. SDS PAGE confirmed the rapid formation of species with 10–25 kDa and suggests that the polymerization appears to be completed within 10 min, as no further band shifting is observed (Supporting Information, Figure S5). The high‐molecular‐weight fraction found by GPC was not resolved in SDS PAGE owing to the molecular weight cut‐off of the gel. Interestingly, the analysis suggests a limitation in primary polymer growth to occur at a molecular weight of about 20–25 kDa. The higher molecular weight fraction is presumably a result of subsequent cross‐linking by secondary reactions such as diDopa formation (Supporting Information, Section S5.12).

**Figure 2:**
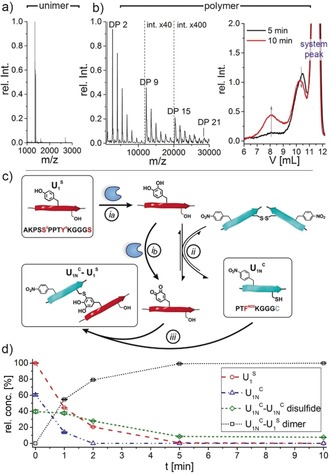
Enzyme‐activated polymerization of U_1_^C^ by tyrosinase to form polyU_1_^C^ (a,b) and model study revealing kinetics of tyrosinase‐induced dimerization of U_1_^S^ and U_1N_^C^ (c,d). MALDI‐TOF‐MS of U_1_^C^ at time zero (a) and of polyU_1_^C^ after 10 min (b, left). GPC traces of polyU_1_^C^ (b, right). c) Reaction pathway for the model dimerization shows enzymatic oxidation of tyrosine in U_1_^S^ to Dopaquinone (*i* *a,b*), U_1N_^C^ oxidation to disulfide by Dopaquinone reduction and the inverse reaction (*ii*) as well as U_1N_^C^‐U_1_^S^ dimer formation by cysteinyldopa linkage (*iii*). d) Relative concentrations of different species during model dimerization determined by HPLC kinetics. (Conditions: 0.25 mm of U_1_^S^ and 0.26 mm U_1N_^C^ (1/1.05, v/v) in pH 6.8 buffer, 50 U mL^−1^ *Ab*PPO4).

For polyU_2_^C^ rather related results are observed (Supporting Information, Figure S6). Considering the additional dispersity that is caused by branching, SDS PAGE analysis shows less defined, broader bands, which reach 100 kDa, though the main band also appears at about 20–25 kDa.

MALDI‐TOF‐MS/MS analysis of the U_1_^C^ polymerization mixture confirmed that the growth mechanism is based on cysteinyldopa linkages (Supporting Information, Section S5.11). The U_1_^C^ dimer species with *m*/*z* 2722.27–2726.30 was fragmented and confirmed the presence of a cysteinyldopa connectivity by showing required y and b fragmentation ions. Moreover, direct proof was provided by the molecular ions that result from the S−C_β_ bond cleavage of the cysteinyldopa species.

The cross‐linking reaction was investigated in a model dimerization of the monofunctional unimers AKPS**S**5PPT**Y**9KGGG**S** (U_1_^S^), that contains one tyrosine but no cysteine, and PT**F^NO2^**KGGG**C** (U_1N_^C^), which bears cysteine while tyrosine was replaced with *p*‐nitrophenylalanine. The dimerization of U_1_^S^ and U_1N_^C^ occurs rapidly after enzymatic oxidation of U_1_^S^ via cysteinyldopa formation. HPLC kinetics indicated the complete dimerization product formation within about 5 min, even at a marginal excess of U_1N_^C^ (Figure 2 d).

Interestingly, a secondary reaction pathway occurred leading to the formation of the disulfide bridged symmetric dimers U_1N_^C^–U_1N_^C^. The presence of redox active partners like Dopa/Dopaquinone is required to generate disulfides, as the control reaction of U_1N_^C^+U_1_^S^ without tyrosinase has shown only 2.5 % disulfide formation after 48 h (Supporting Information, Figure S16). Thiol oxidation to disulfide can be promoted by reduction of Dopaquinone to Dopa, as has been described for the rescue protein Mcfp‐6, which restores Dopa functionalities under the oxidizing seawater conditions.^18^ Considering this remarkable analogy, it is likely that the reduction of Dopaquinone leads to the formation of disulfide and Dopa (Figure 2 c, reaction *ii*). Intriguingly, the U_1N_^C^–U_1N_^C^ dimer is not a dead end, since the redox potential of the reduction of disulfides by Dopa is close to that of the oxidation of thiol by Dopaquinone.^18^ Thus, reaction *ii* remains reversible and U_1N_^C^ can be regenerated to undergo the favored formation of cysteinyldopa linked U_1N_^C^–U_1_^S^ dimers and drives the polymerization (reaction *iii*). Residual disulfide corresponds to the small excess of U_1N_^C^ that was used to reach equivalency of effective functional groups.

The model system was further employed to illuminate potential secondary cross‐linking routes. Upon enzymatic activation of U_1_^S^ in the absence of U_1N_^C^, no polymer was detectable by SDS PAGE. This suggests the need for cysteine to enable polymerization and confirms the lower reactivity of *ϵ*‐amino groups of Lys2 and Lys10 (Supporting Information, Figure S7). However, minor amounts of the dimerization product U_1_^S^–U_1_^S^ were found after 1 h reaction time by MALDI‐TOF‐MS. ESI‐LC‐MS/MS confirmed the nature of the connectivity to be a 5,5′‐diDopa linkage as directly associated fragment ions were observed (Supporting Information, Section S5.12). None of the y, b, c, and z ions or other fragments gave evidence for lysinyldopa links. Apparently, cysteinyldopa groups are less susceptible for further coupling reactions as no subsequent cross‐linking of cysteinyldopa linked U_1_^S^–U_1N_^C^ dimers was observed in a model reaction over 48 h. Conclusively, secondary cross‐linking can occur via 5,5′‐diDopa formation by unreacted Dopaquinones, which proceeds slower than producing the cysteinyldopa linkages. This evidence supports the hypothesis that the polymerization is driven by cysteinyldopa formation to generate primary polymerization products. These can further cross‐link via secondary reactions to form high molecular weight fractions.

One of the intriguing aspects of the polymerization is the generation of cysteinyldopa functionalities at each repeat unit, which provide catechol structures and promise high surface‐binding capabilities. A quartz‐crystal microbalance with dissipation (QCM‐D) was used to gain insights into the adsorption behavior as well as coating stability of polyU_1_^C^ and polyU_2_^C^. Both mussel foot protein analogues show rapid adsorption from aqueous solutions onto QCM sensors, exposing either alumina or fluoropolymer surfaces (Figure 3; Supporting Information, Sections S5.13 and S5.14). Multilayer formation occurs and prevents to reach equilibrium, as it is typical for protein adsorption processes. Swelling of the polyU_2_^C^ coating on alumina is indicated by the gradual decrease in Δ*f* during buffer rinsing (Supporting Information, Figure S30). Independent of the substrate, all of the coatings almost completely defy extensive rinsing with buffer and saline seawater equivalents (599 mm NaCl). Most impressively, the coatings withstand hypersaline conditions of 4.2 m NaCl as present in water of the Dead Sea, showing negligible mass losses of 1–7 %. Hence, the coating systems proved notable adhesion and robust cohesion stabilities.

**Figure 3:**
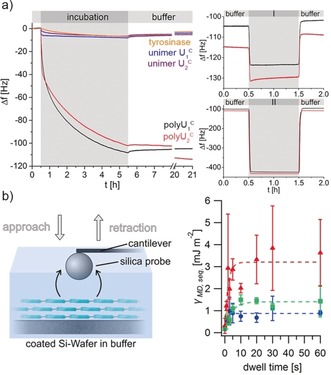
Adsorption (a) and adhesion (b) behavior of polyU_1_^C^ and polyU_2_^C^. a) QCM‐D adsorption and desorption kinetics of mfp analogues on Al_2_O_3_ coated sensors (incubation step and subsequent buffer rinsing (left) and stability tests of the coatings (right) by washing with 599 mm NaCl (I) and 4.2 m hypersaline solution (ΙΙ)). b) Adhesive properties of mfp analogue determined by colloidal probe AFM. Illustration of the CP‐AFM contact analysis (left) and work of adhesion per unit area of polyU_1_^C^ coatings in dependence of the dwell time (right; substrates were coated for 1 h (blue), for 2 h (green) and 1 h+post‐treatment with sodium ascorbate (red); constant load force of 2 nN; lines are to guide the eye only).

QCM data evaluation, applying the Voight model for viscoelastic films,^19^ enables the estimation of areal mass densities of 40±5 mg m^−2^ and 30±4 mg m^−2^ with corresponding layer thicknesses of 34±4 nm and 26±2 nm for polyU_1_^C^ and polyU_2_^C^, respectively. Using the Sauerbrey model^20^ for rigid films to calculate adsorbed polymer masses leads to lower areal mass densities and thus suggests a certain viscoelasticity of the polymer films (Supporting Information, Table S4). This is supported by the fact that a pronounced frequency dependence in the Δ*f* and Δ*D* response is visible for the coatings (Supporting Information, Figures S27, S35). By comparing the values of the different models it can be deduced that the polyU_2_^C^ films give more rigid coatings, since the deviation between both models is lower than for polyU_1_^C^ coatings. This can be rationalized by the different types of net‐points in the coatings: while polyU_1_^C^ shows only non‐covalent interchain contacts, the branched topology of polyU_2_^C^ has instead covalent net‐points that increase network rigidity. This is consistent with covalently cross‐linked Mefp‐1 proteins that form more rigid coatings, that better suit the Sauerbrey regime.^21^ A similar trend for coatings of polyU_1_^C^ and polyU_2_^C^ with respect to masses and thicknesses on alumina surfaces is evident for coatings on fluoropolymer substrates (Supporting Information, Figure S43). The latter are known to be highly challenging for coating, on which nonetheless marine mussels can effectively adhere well.^22^ Estimated areal mass densities of 31±3 mg m^−2^ and 18±1 mg m^−2^ (Voight model) give theoretical layer thicknesses of 26±2 nm and 15±1 nm for polyU_1_^C^ and polyU_2_^C^, respectively. The mass deposition kinetics onto fluoropolymer substrates are initially slightly faster than on alumina, but after 5 h of coating time the final areal mass density is lower. This can be correlated to the priming step in the film formation process. Depending on the surface, the priming involves different contacts between peptide and substrate, which will have an influence on follow‐up multilayer deposition.

Ultimately, QCM‐D experiments revealed that significantly different surfaces could be effectively coated, leading to stable coatings tolerant to harsh conditions. Both the cysteinyldopa connectivities and the repetitive mfp‐1 consensus sequence were probably synergistically contributing to the properties, as the unimers and the enzyme alone led to negligible adsorption.

After confirming the exceptional coating behavior of the mussel foot protein analogues, the wet adhesive performance of polyU_1_^C^ and polyU_2_^C^ was investigated with colloidal probe atomic force microscopy (CP‐AFM). PolyU_1_^C^ and polyU_2_^C^ were adsorbed onto passivated silicon wafers for 1 h and 2 h, respectively. Adhesive interactions of the coatings were quantified in force vs. distance measurements with a spherical silica probe of 2.4 μm radius. The probe was approached to the coated substrates, varying the maximum load (2–50 nN) and resting dwell times (0–60 s; Supporting Information, Figure S48). With increasing dwell times, the adhesion of the probe increases in a non‐linear fashion for the entire sample set (Supporting Information, Figure S49; Figure 3 b). This correlates with a dynamic character of the interface in the contact area. Both coating polymers will rearrange during contact, thereby optimizing adhesive interactions with the probe. As the linear polyU_1_^C^ offers higher mobility, work of adhesion at 60 s dwell time reaches higher values of 1.4 mJ m^−2^, compared to 0.5 mJ m^−2^ for the branched and less flexible polyU_2_^C^ (Figure 3 b (2 h); Supporting Information, Figure S49). The adhesion can be increased significantly to 3.6 mJ m^−2^ and 0.9 mJ m^−2^ at 60 s dwell time by applying sodium ascorbate antioxidant to the polymer coatings of polyU_1_^C^ and polyU_2_^C^. This suggests that oxidation of cysteinyldopa to quinone derivatives might occur during sample preparation and measurements, where the presence of antioxidants can regenerate the dopa functionalities. The highest single value that was observed under such conditions reached 10.9 mJ m^−2^ for polyU_1_^C^ coatings at 30 s dwell time and 20 nN load.

As expected, complex protein‐based adhesives like the native mfps exhibit individual surface binding behavior and adaptation dynamics. For instance, silica adhesion of mfp‐1, which is related to the synthetic polyU_1_^C^, showed only low work of adhesion of 0.1 mJ m^−2^ at pH 5.5.^23^ It is remarkable that the artificial mfps provide considerably higher work of adhesion. Moreover, the specialized mfp‐3 and mfp‐5 that constitute the adhesion interface of mussels, reached maximum adhesive energy values at pH 5.5 on silica of 2.99 and 2.44 mJ m^−2^ with a contact time of 60 min,^23^ respectively, which are still below the average work of adhesion attained by polyU_1_^C^ after only 60 s of contact time. It has also to be noted that a complete loss of adhesive function was reported at pH 7–7.5^22^ for several mfps, including mfp‐1, whereas the artificial mfp analogues are able to perform at pH 6.8.

Since there is only limited adhesion data for native mfps and absolute comparison is often difficult, CP‐AFM reference experiments with a commercially available protein extract from *Mytilus edulis* (Cell‐Tak) were performed. The control experiments were carried out under the same conditions as for polyU_1_^C^ (Supporting Information, Figure S50). CP‐AFM revealed adhesion energies in a similar range, with an average of 3.9 mJ m^−2^ after 60 s dwell time and addition of ascorbate at 2 nN load force. Considering that Cell‐Tak is an adhesive optimized for neutral pH, which consists of a mixture of mfps, wherein Mefp‐1 is one component, and isolated Mefp‐1 showed the lowest adhesion properties in previous studies,^24^ the polyU_1_^C^ performs very well. The artificial mfp analogue seems to constitute a capable mfp‐1 mimic by just relying on the consensus decapeptide of Mefp‐1, but still leaves room for further improvements by sequence adaptation.

In conclusion, a novel tyrosinase‐activated polymerization route to mussel foot protein (mfp) analogues was introduced. Polymerization of peptide‐based unimers could be achieved using cysteinyldopa linking of an analogue of the Mefp‐1 consensus decapeptide. Within a few minutes, polymers with an apparent molecular weight of up to 530 kDa are generated. The resulting artificial mfp analogues show versatile adsorption and result in coatings having excellent resistance to harsh hypersaline conditions, as demonstrated by QCM‐D measurements. The adhesive energies in thin‐film CP‐AFM studies on silica surfaces are superior to values reported from surface apparatus studies on isolated mfp‐1 as well as on isolated gluing interface proteins (mfp‐3 and mfp‐5). Moreover, similar adhesive energy ranges are achieved as with a commercial mussel foot protein extract from *Mytilus edulis*, which is optimized for adhesion at neutral pH. The tyrosinase‐induced polymerization of peptides offers facile access to artificial mfp analogues and avoids the complexity of naturally derived proteins. Next generation universal glues can be envisioned that perform effectively even under rigorous seawater conditions and adapt to a broad range of difficult surfaces.

## Conflict of interest

The authors declare no conflict of interest.

## Supporting information

As a service to our authors and readers, this journal provides supporting information supplied by the authors. Such materials are peer reviewed and may be re‐organized for online delivery, but are not copy‐edited or typeset. Technical support issues arising from supporting information (other than missing files) should be addressed to the authors.

Supplementary

## References

[1a] H. Lee , B. P. Lee , P. B. Messersmith , Nature 2007, 448, 338–341; 17637666 10.1038/nature05968

[1b] B. K. Ahn , S. Das , R. Linstadt , Y. Kaufman , N. R. Martinez-Rodriguez , R. Mirshafian , E. Kesselman , Y. Talmon , B. H. Lipshutz , J. N. Israelachvili , J. H. Waite , Nat. Commun. 2015, 6, 8663; 26478273 10.1038/ncomms9663 PMC4667698

[1c] P. Wilke , N. Helfricht , A. Mark , G. Papastavrou , D. Faivre , H. G. Börner , J. Am. Chem. Soc. 2014, 136, 12667—12674; 25133879 10.1021/ja505413e

[1d] Q. Wei , K. Achazi , H. Liebe , A. Schulz , P.-L. M. Noeske , I. Grunwald , R. Haag , Angew. Chem. Int. Ed. 2014, 53, 11650–11655; 10.1002/anie.201407113 25200129

[1e] H. Woehlk , J. Steinkoenig , C. Lang , L. Michalek , V. Trouillet , P. Krolla , A. S. Goldmann , L. Barner , J. P. Blinco , C. Barner-Kowollik , K. E. Fairfull-Smith , Langmuir 2018, 34, 3264–3274. 29442516 10.1021/acs.langmuir.7b03755

[2] J. H. Waiter , Ann. N. Y. Acad. Sci. 1999, 875, 301–309. 10415577 10.1111/j.1749-6632.1999.tb08513.x

[3a] E. Faure , C. Falentin-Daudré , C. Jérôme , J. Lyskawa , D. Fournier , P. Woisel , C. Detrembleur , Prog. Polym. Sci. 2013, 38, 236–270;

[3b] B. P. Lee , P. B. Messersmith , J. N. Israelachvili , J. H. Waite , Annu. Rev. Mater. Res. 2011, 41, 99–132. 22058660 10.1146/annurev-matsci-062910-100429 PMC3207216

[4a] B. P. Lee , J. L. Dalsin , P. B. Messersmith , Biomacromolecules 2002, 3, 1038–1047; 12217051 10.1021/bm025546n

[4b] P. Wilke , H. G. Börner , ACS Macro Lett. 2012, 1, 871–875; 10.1021/mz300258m 35607135

[4c] A. Lampel , S. A. McPhee , H.-A. Park , G. G. Scott , S. Humagain , D. R. Hekstra , B. Yoo , P. W. J. M. Frederix , T.-D. Li , R. R. Abzalimov , S. G. Greenbaum , T. Tuttle , C. Hu , C. J. Bettinger , R. V. Ulijn , Science 2017, 356, 1064–1068. 28596363 10.1126/science.aal5005

[5a] D. S. Hwang , H. Zeng , Q. Lu , J. Israelachvili , J. H. Waite , Soft Matter 2012, 8, 5640–5648; 23105946 10.1039/C2SM25173F PMC3482130

[5b] Q. Lu , D. S. Hwang , Y. Liu , H. Zeng , Biomaterials 2012, 33, 1903–1911; 22138031 10.1016/j.biomaterials.2011.11.021

[5c] Q. Lu , D. X. Oh , Y. Lee , Y. Jho , D. S. Hwang , H. Zeng , Angew. Chem. Int. Ed. 2013, 52, 3944–3948; 10.1002/anie.201210365 23450629

[6] J. H. Waite , Comp. Biochem. Physiol. Part B 1990, 97, 19–29. 10.1016/0305-0491(90)90172-p 2123765

[7] M. J. Harrington , A. Masic , N. Holten-Andersen , J. H. Waite , P. Fratzl , Science 2010, 328, 216–220. 20203014 10.1126/science.1181044 PMC3087814

[8] L. M. McDowell , L. A. Burzio , J. H. Waite , J. Schaefer , J. Biol. Chem. 1999, 274, 20293–20295. 10400649 10.1074/jbc.274.29.20293

[9] L. V. Zuccarello , Tissue Cell 1981, 13, 701–713. 6800062 10.1016/s0040-8166(81)80007-9

[10] M. Yu , J. Hwang , T. J. Deming , J. Am. Chem. Soc. 1999, 121, 5825–5826.

[11a] T. Kato , S. Ito , K. Fujita , Biochim. Biophys. Acta Gen. Subj. 1986, 881, 415–421; 10.1016/0304-4165(86)90034-6 2938636

[11b] D. C. S. Tse , R. L. McCreery , R. N. Adams , J. Med. Chem. 1976, 19, 37–40. 1246050 10.1021/jm00223a008

[12a] H. Zhao , J. H. Waite , Biochemistry 2005, 44, 15915–15923; 16313194 10.1021/bi051530g PMC1892533

[12b] H. Zhao , J. H. Waite , J. Biol. Chem. 2006, 281, 26150–26158. 16844688 10.1074/jbc.M604357200

[13] J. H. Waite , J. Biol. Chem. 1983, 258, 2911–2915. 6298211

[14] M. Pretzler , A. Bijelic , A. Rompel , Sci. Rep. 2017, 7, 1810. 28500345 10.1038/s41598-017-01813-1 PMC5431950

[15] A. Flurkey , J. Cooksey , A. Reddy , K. Spoonmore , A. Rescigno , J. Inlow , W. H. Flurkey , J. Agric. Food Chem. 2008, 56, 4760–4768. 18500813 10.1021/jf800109a

[16] S. G. Mauracher , C. Molitor , C. Michael , M. Kragl , A. Rizzi , A. Rompel , Phytochemistry 2014, 99, 14–25. 24461779 10.1016/j.phytochem.2013.12.016 PMC3969299

[17] T. S. Fischer , J. Steinkoenig , H. Woehlk , J. P. Blinco , K. Fairfull-Smith , C. Barner-Kowollik , Polym. Chem. 2017, 8, 5269–5274.

[18] S. C. T. Nicklisch , J. E. Spahn , H. Zhou , C. M. Gruian , J. H. Waite , Biochemistry 2016, 55, 2022–2030. 26998552 10.1021/acs.biochem.6b00044 PMC4934423

[19] M. V. Voinova , M. Rodahl , M. Jonson , B. Kasemo , Phys. Scr. 1999, 59, 391.

[20] G. Sauerbrey , Z. Phys. 1959, 155, 206–222.

[21] F. Höök , B. Kasemo , T. Nylander , C. Fant , K. Sott , H. Elwing , Anal. Chem. 2001, 73, 5796–5804. 11791547 10.1021/ac0106501

[22] Q. Lin , D. Gourdon , C. Sun , N. Holten-Andersen , T. H. Anderson , J. H. Waite , J. N. Israelachvili , Proc. Natl. Acad. Sci. USA 2007, 104, 3782–3786. 17360430 10.1073/pnas.0607852104 PMC1820661

[23] Q. Lu , E. Danner , J. H. Waite , J. N. Israelachvili , H. Zeng , D. S. Hwang , J. R. Soc. Interface 2013, 10, 20120759. 23173195 10.1098/rsif.2012.0759 PMC3565691

[24] J. Yu , Y. Kan , M. Rapp , E. Danner , W. Wei , S. Das , D. R. Miller , Y. Chen , J. H. Waite , J. N. Israelachvili , Proc. Natl. Acad. Sci. USA 2013, 110, 15680–15685. 24014592 10.1073/pnas.1315015110 PMC3785749

