# Supplementary material for: Polymerizing Like Mussels Do: Toward Synthetic Mussel Foot Proteins and Resistant Glues
Source: Angew Chem Int Ed Engl. 2018 Oct 31;57(48):15728–32. doi: 10.1002/anie.201809587 (PMC6282983; doi:10.1002/anie.201809587)
Supplement: Supplementary file 1 — Supplementary [file ANIE-57-15728-s001.pdf]

Angewandte Chemie
Eine Zeitschrift der Gesellschaft Deutscher Chemiker

Supporting Information

Polymerizing Like Mussels Do: Toward Synthetic Mussel Foot Proteins and Resistant Glues

*Justus Horsch, Patrick Wilke, Matthias Pretzler, Maximilian Seuss, Inga Melnyk, Dario Remmler, Andreas Fery, Annette Rompel, and Hans G. Börner**

anie_201809587_sm_miscellaneous_information.pdf

Table of Contents

1. Materials **S1**
1.1 Peptide Synthesis S1
1.2 SDS-PAGE S2
1.3 Enzymatic assay and reactions S2

2. Instrumentation **S2**

3. Methods **S4**
3.1 Peptide synthesis S4
3.2 Preparation of AbPPO4 tyrosinase from common mushrooms S5
3.3 AbPPO4 activity assay S5
3.4 Enzymatic unimer activation and polymerization reactions S6
3.5 Quartz crystal microbalance S6
3.6 SDS PAGE S6
3.7 Atomic force microscopy S7

4. Compound Characterization **S8**
4.1 U_1^C – AKPSSPPTYKGGGC S8
4.2 U_2^C – AKPSYPPTYKGGGC S9
4.3 U_1^S – AKPSSPPTYKGGGS S10
4.4 U_{1N}^C – PTF(4-NO_2)KGGGC S11

5. Experiments **S12**
5.1 MALDI-TOF polymerization kinetics of polyU_1^C S12
5.2 MALDI-TOF polymerization kinetics of polyU_2^C S14
5.3 SDS PAGE polymerization kinetics for U_1^C S16
5.4 SDS PAGE polymerization kinetics for U_2^C S17
5.5 SDS PAGE unimer zero reference S18
5.6 GPC kinetic measurements of polyU_1^C S19
5.7 SDS PAGE measurement of activated U_1^S S21
5.8 HPLC kinetic measurements of U_1^S activation S22
5.9 HPLC kinetic dimerization measurements of the model system S24
5.10 HPLC kinetic measurements of disulfide formation of U_{1N}^C S26
5.11 MALDI-TOF-MS/MS measurements of polyU_1^C S27
5.12 LC-ESI-MS/MS cross-linking experiments S30
5.13 QCM-D experiments on aluminium oxide surface S35
5.14 QCM-D experiments on fluoropolymer surface S43
5.15 Comparison of QCM-D kinetics and areal mass density S51
5.16 Colloidal Probe Atomic Force Microscopy to Characterize Adhesion Properties. S52

6. References **S61**

1. Materials

Calcium chloride dihydrate (≥99.5%), sodium citrate tribasic dihydrate (>98%), potassium chloride (≥99.5%), α-cyano-4-hydroxy-cinnamic acid (α-CHCA, 99%) and Formic acid (FA, ~98%) were purchased from Sigma Aldrich (Seelze, Germany). Acetonitrile (HPLC-MS grade) ethanol absolute (>99.7%) and hydrochloric acid (37%) were obtained from VWR chemicals (Philadelphia, USA). Citric acid (≥99.5%), sodium sulfate (≥99%), sodium hydrogen carbonate (≥99.5%), sodium bromide (≥99%) and strontium chloride hexahydrate (≥99%) were received from Carl Roth GmbH (Karlsruhe, Germany). Boric acid (99%) and magnesium chloride hexahydrate (98%) were purchased from abcr GmbH (Karlsruhe, Germany). Sodium chloride (≥99%) and potassium bromide (≥99%) were obtained from Acros Organics (Geel, Belgium). Hellmanex III was acquired from Hellma GmbH (Müllheim, Germany). Cell-Tak™ was purchased from Corning (Tewksbury, MA, USA). All chemicals were used as received without further purification.

All buffers and aqueous solutions were prepared with Milli-Q water.

1.1 Peptide Synthesis

N-α-Fmoc protected amino acids Fmoc-Ala-OH, Fmoc-Cys(Trt)-OH, Fmoc-Gly-OH, Fmoc-Lys(Boc)-OH, Fmoc-Pro-OH, Fmoc-Ser(*t*Bu)-OH, Fmoc-Thr(*t*Bu)-OH, Fmoc-Tyr(Boc)-OH, scavenger trimethyl silylbromide (TMSBr) as well as coupling reagents 2-(1H-benzotriazol-1-yl)-1,1,3,3-tetramethyluronoium hexafluorophosphate (HBTU), Benzotriazole-1-yl-oxy-tris-pyrrolidino-phosphonium hexafluorophosphate (PyBOP), and *N*-methyl-2-pyrrolidone (NMP, 99.9%, peptide synthesis grade) were used as received from IRIS Biotech GmbH (Marktredwitz, Germany). TentaGel S RAM resin (loading: 0.24 mmol/g) was obtained from Rapp Polymere GmbH (Tübingen, Germany). *N,N*-diisopropyl ethylamine (DIPEA, peptide grade), piperidine (peptide grade), 2,5-dihydroxybenzoic acid (99%) were purchased from Acros Organics (Geel, Belgium) and used without further purification. Triethylsilane (TES, 98+ %) was obtained from Alfa Aesar (Karlsruhe, Germany). Trifluoroacetic acid (TFA, peptide grade) from Acros Organics (Geel, Belgium) was distilled prior to use. Dichloromethane (DCM, peptide grade) from IRIS Biotech GmbH (Marktredwitz, Germany) was distilled from CaH_2 prior to use. Fmoc-Phe(4-NO_2)-OH was used as received from Merck KGaA (Darmstadt, Germany).

1.2 SDS-PAGE

Dodecyl sulfate sodium salt (85%) was purchased from Acros Organics (Geel, Belgium). Glycine (≥99%) was obtained from Sigma Aldrich (Seelze, Germany). Tris(hydroxymethyl)aminomethane (Tris, ≥99.9%) was acquired from Carl Roth GmbH (Karlsruhe, Germany). The PageRuler prestained protein ladder (10 - 180 kDa), the Pierce lane marker non-reducing sample buffer and the Pierce silver stain kit were purchased from Thermo Fisher Scientific (Waltham, MA, USA). The protein ladder and the lane marker were stored at -20 °C.

1.3 Enzymatic assay and reactions

The enzyme *Ab*PPO4 was prepared as described earlier (cf. section 3.2)[1]. Lyophilisates of the enzyme from sodium citrate buffer (50 mM, pH 6.8) were stored at -20 °C and dissolved in Milli-Q water prior to use. Enzyme solutions were stored at -20 °C as well. L-Tyrosine (≥98%) was obtained from Sigma Aldrich (Seelze, Germany) and L(+)-ascorbic acid (≥99%) was purchased from Carl Roth GmbH (Karlsruhe, Germany).

2. Instrumentation

UV/VIS spectroscopy (enzymatic assay) was carried out on an EonC Microplate Spectrophotometer with cuvette port (BioTek, Bad Friedrichshall, Germany) using quartz cuvettes.

MALDI-TOF mass spectrometry was performed on an autoflex III smartbeam system (Bruker, USA) with matrix assisted laser desorption/ionization and time of flight detector. On the sample plate, 2 µL of sample were mixed with 1 µL matrix solution, consisting either of 7 mg/mL α-cyano-4-hydroxy-cinnamic acid (CHCA) or 10 mg/mL 2,5-dihydroxybenzoic acid (DHB) in MQ-water-acetonitrile (1:1, v/v) with 0.1 % TFA. Samples were air-dried at ambient temperature. Measurements were performed in linear positive mode. Gating and deflection modes were used for detection of the higher mass area (m/z > 10.000).

MALDI-TOF-MS/MS measurements were conducted on a 5800 MALDI-TOF/TOF system (AB Sciex, USA). Samples were mixed in a 1:1 ratio with matrix solution, consisting of 7 mg/mL α-cyano-4-hydroxy-cinnamic acid in MQ-water-acetonitrile (1:1, v/v) with 0.1 % TFA, on the sample plate and air dried at ambient temperature. Measurements were performed in

reflector positive mode, with 4000 shots and laser intensity of 3200 for MS and 12500 shots and laser intensity of 4300 for MS/MS.

LC-ESI-MS/MS has been carried out on an Dionex UltiMate 3000 RSLCnano LC system (Thermo Scientific, USA) equipped with NCS-3500RS Binary Rapid Separation Nano/Capillary Pumps, a homemade InfinityLab Poroshell 120 EC-C18 pikoTip column (75 µm x 50 cm, Agilent, USA), an Acclaim PepMap 100 C18 precollumn (75 µm x 20 mm, Thermo Scientific, USA) and an Orbitrap Fusion Tribrid mass spectrometer (Thermo Scientific, USA). Measurements were performed with EThcd fragmentation using charge depending calibrated ETD parameters and 30% Collision energy at an isolation width of 1.2 Da.

Analytical HPLC was performed on a Shimadzu (Japan) system using a SCL-10A vp system controller, a SPD-M10A vp diode array detector, a LC-10AD vp liquid chromatograph pump unit and a CTO-10AC vp column oven equipped with a YMC-Pack ODS-AQ column (250 x 4.6 mm, YMC, Japan). For preparative HPLC a Shimadzu prominence system with a CBM-20A communications bus module, a LC-20AP preparative liquid chromatograph pump unit, a SPD-10A UV/VIS detector and a FRC-10A fraction collector was used. Chromatographic separation was conducted on a Synergi Fusion-RP column (250 x 21.2 mm, phenomenex, Germany). As solvent, mixtures of solvent A/solvent B (solvent A: 99.9% Milli-Q H_2O - 0.1% FA; solvent B: 99.9% acetonitrile - 0.1% FA) were used for both systems with a flow rate of 1.0 and 22.0 mL/min respectively.

UPLC-QMS was carried out on an Acquity UPLC H-class system (Waters, USA) with a PDA and QDa detector. Acquity UPLC BEH C18 columns (2.1 x 100 mm, 2.1 x 50 mm, Waters, USA) were used for chromatographic separation with a solvent mixture of solvent A/solvent B (solvent A: 99.9% Milli-Q H_2O - 0.1% FA; solvent B: 99.9% acetonitrile - 0.1% FA) and a flow rate of 0.5 mL/min.

Quartz crystal microbalance measurements were conducted on a Q-sense Explorer E1 single-sensor QCM-D module with dissipation combined with a QE 401 Electronic Unit (Biolin Scientific, Sweden) and equipped with a IPC-N 4 multichannel pump (Ismatec, Germany). Piezoelectric sensor crystals coated with 100 nm aluminum oxide (QSX 309, Biolin Scientific, Sweden) and with fluoropolymer (QSX 331 AF 1600, Biolin Scientific, Sweden) were used for adsorption measurements.

SDS-PAGE measurements were performed in a Mini-PROTEAN tetra system cell (Bio-Rad, USA) with purchased 4 - 20% gel percentage precast polyacrylamide Mini-PROTEAN TGX gels (Bio-Rad, USA). As running buffer a solution of 25 mM Tris, 192 mM glycine, 0.1% SDS in Milli-Q water was used. Staining was done according to standard protocol with a Pierce silver stain kit (Thermo Fisher Scientific, USA).

Aqueous GPC was carried out on a NOVEMA Max analytical linear XL column (PSS, Germany) calibrated with pullulanes with an AS-100 autosampler, P-100 pump (TSP Thermo Separation Products, Germany) and Shodex RI-101 detector (VDS-optilab, Germany). As mobile phase acetate buffer (100 mM, pH 4.5):methanol, 4:1 (v/v) was used with a flow rate of 1.0 mL/min. Data were recorded and evaluated with the PSS-WinGPC Unichrom software package.

AFM imaging and adhesion characterization was performed on a MFP-3D Bio (Asylum Research, An Oxford Instruments Company, Santa Barbara, CA) equipped with a top view optic. Protein coated silicon wafer were fixed in the closed fluid cell with a home-made inset and kept hydrated with sodium citrate buffer. For adhesion measurements two cantilevers (CSC 38, mikromasch Europe, Wetzlar, Germany) with thermal noise calibrated spring constants of 116 pN/nm and 245 pN/nm, respectively, were modified with silica colloidal probes (SiO_2-Forschungspartikel, diameter 4.8 µm, micro particles GmbH, Berlin, Germany). Force-distance measurements were performed with a fixed velocity of 2 µm/s while the dwell times on the surface were systematically varied between 0 s and 60 s and the load force between 2 nN and 50 nN. AFM images were recorded in sodium citrate buffer using the iDrive technique (Asylum Research, An Oxford Instruments Company, Santa Barbara, CA) as well as standard tapping mode.

3. Methods

3.1 Peptide synthesis

Peptides were synthesized following standard ABI-*Fastmoc* protocol (single coupling with capping) with NMP as solvent using standard Fmoc-amino acid derivatives. As solid support, TentaGel S RAM resin (loading 0.24 mmol/g, 0.1 mmol) was used. Synthesis was performed on an automated *ABI 433a* peptide synthesizer (Applied Biosystems, Foster City, USA). Fmoc-amino acid coupling was facilitated by HBTU/DIPEA. After final Fmoc removal the resin was transferred to a 10 mL syringe reactor and subsequently washed with dichloromethane. Peptides were cleaved from the solid support with a mixture of 95:4:1 vol.% TFA/H_2O/TES for 3 h,

which resulted in fully deprotected peptide. The resin was filtered, washed with TFA and the collected supernatants were concentrated *in vacuo*. The product was isolated by precipitation with diethyl ether and subsequent centrifugation. Purified products were obtained by lyophilization from Milli-Q water.

In total, the four peptides AKPSYPPTYKGGGC (U_2^C), AKPSSPPTYKGGGC (U_1^C), AKPSSPPTYKGGGS (U_1^S) and PTF(4-NO_2)KGGC (U_{1N}^C) [F(4-NO_2)-4-nitrophenylalanine] were obtained by this method.

3.2 Preparation of AbPPO4 tyrosinase from common mushrooms

The gene encoding *Ab*PPO4 was PCR-amplified from cDNA derived from an *A. bisporus* fruiting body at growth stage 5[2] and cloned into the expression vector pGEX-6P-1 (GE Healthcare Europe, Freiburg, Germany). The resulting construct encoding glutathione S-transferase (GST) N-terminally fused to *Ab*PPO4 was expressed in *E. coli* BL21(DE3) grown in LB media supplemented with 2 mM $MgSO_4$, 500 mM NaCl, 1x mineral stock M[3], 1x sugar stock 5052[3], 100 mg l^{-1} Na-ampicillin and 0.5 mM $CuSO_4$ at 20 °C for approximately 40 h. Cells were lysed by high-pressure extrusion[4] and non-target proteins were removed by affinity chromatography on Glutathione Sepharose (GE Healthcare). The fusion partner GST was removed by proteolysis with GST-tagged picornain 3C which was afterwards removed along with the cleaved-off GST by a second round of affinity chromatography on the same column material. The resulting latent *Ab*PPO4 was activated by limited proteolysis with proteinase K and the active *Ab*PPO4 was purified by size exclusion chromatography on a Superdex 200 Increase column (GE Healthcare).

3.3 AbPPO4 activity assay

Prior to use of enzyme, an activity assay was performed using UV spectroscopy based on the method of *Duckworth* and *Coleman*[5]. The absorbance from the oxidation of tyrosine to Dopaquinone is monitored at 280 nm over a period of 20 min at 25 °C using a 3 mL quartz cuvette. The assay solution contained 1 mL of sodium citrate buffer (50 mM, pH 6.8), 1 mL of tyrosine solution (1 mM in Milli-Q water), 0.9 mL of Milli-Q water and 0.1 mL of *Ab*PPO4 enzyme solution (in 50 mM sodium citrate buffer, pH 6.8). Enzyme solution was added immediately before starting the measurement. The activity was calculated using the average slope of the 3 minute interval Δ_{A280} with the maximum slope of the absorbance-time curve according to equation 3.3.1.

$$volumetric\ enzyme\ activity\left[\frac{Units}{mL}\right]=\frac{\Delta_{A280[min^{-1}]}}{0.0001} \qquad \text{(eq. 3.3.1)}$$

3.4 Enzymatic unimer activation and polymerization reactions

Standard enzymatic activation reactions were performed using a substrate concentration of 0.25 µmol/mL (from 1.0 mM stock solutions) and 100 U/mL of *Ab*PPO4 tyrosinase in a sodium citrate buffer solution (17 mM, pH 6.8) at 25 °C. *Ab*PPO4 was mixed with 0.7 nmol/U ascorbic acid as an activator for the enzyme prior to addition to the substrate solution. In polymerization reactions for GPC, AFM and QCM experiments, 0.75 µmol/mL substrate concentration and 50 U/mL *Ab*PPO4 were used.

3.5 Quartz crystal microbalance

The piezoelectric sensor crystals coated with 100 nm aluminum oxide (QSX 309, Biolin Scientific, Sweden) were cleaned with 2% Hellmanex III solution (in Milli-Q water) for 15 - 30 min and ethanol (absolute, >99.7%) in an ultrasonic bath for 10 min prior to use. Subsequently, the sensors were thoroughly washed with Milli-Q water and dried under a compressed air flow. Finally, the aluminium oxide coated crystals were cleaned by air plasma in a ZEPTO plasma cleaner (diener electronic GmbH, Germany) for 3 min at 75 W. The fluoropolymer coated sensor crystals (QSX 331 AF 1600, Biolin Scientific, Sweden) were cleaned in 1% Hellmanex III solution (in Milli-Q water) for 30 min, in Milli-Q water for 2 h and rinsed with ethanol (absolute, >99.7%). Subsequently, the sensors were thoroughly washed with Milli-Q water and dried under a compressed air flow.

The sensors were mounted into the QCM flow chamber and incubated with degassed buffer using a flow rate of 100 µL/min until the frequency signals were constant (1-3 h). The measurement was started and sample solutions were pumped into the flow chamber. Experiments were performed at 22° C in a stop-flow mode, and overtones 3, 5, 7, 9, 11 and 13 were recorded. The third overtones of all experiments were used for evaluation of the frequency shift.

3.6 SDS PAGE

Samples for gel electrophoresis as well as the protein ladder were diluted with Milli-Q water to a volume of 20 µL and subsequently 5 µL of lane marker were added and mixed. The gel cassettes were clamped into the electrode assembly and the assembly and the tank were filled with approximately 800 mL of running buffer (25 mM Tris, 192 mM glycine, 0.1% SDS in Milli-Q water). Samples of 20 µL were loaded into the wells of the gel using a 10-100 µL pipette and runs were performed at 140 V until the lane marker reached the lower end of the gel. Staining was performed using silver stain (Thermo Fisher Scientific, USA) according to the manufacturer’s standard protocol.

3.7 Atomic force microscopy

For adhesion measurements silicon wafers were coated with the polymers polyU$_1^C$ and polyU$_2^C$ for 1 and 2 h of coating time. The coating solutions were prepared according to procedure 3.4 using a unimer concentration of 0.75 mM and an *Ab*PPO4 tyrosinase concentration of 50 U/mL. The wafers were cleaned prior to use with RCA (H_2O:NH_3:H_2O_2, 5:1:1 v/v) at 80 °C for 10 min followed by excessive rinsing with MilliQ-water and O_2 plasma cleaning (0.2 mbar, 100W, 1 min; 440-G, TePla, Wettenberg, Germany). The samples were stored in sodium citrate buffer (17 mM, pH 6.8) not longer than 4 days. For AFM measurements the samples were transferred to a closed fluid cell, fixed at the edges with two screws, and the cell was completely filled with sodium citrate buffer. Adhesion experiments were conducted with two tipless cantilevers (CSC 38, Mikromasch Europe, Wetzlar, Germany) equipped with a silica colloidal probe (radius 2.4 µm). Prior to use the cantilevers were O_2 plasma treated (0.2 mbar, 100 W, 1 min; 440-G, TePla, Wettenberg, Germany) to activate the silica surface and establish reproducible conditions. Force calibration of the cantilevers was achieved by thermal noise (spring constant)[6] and by pressing against a non-deformable surface (lever sensitivity)[7]. Adhesion interactions were recorded by manually lowering the cantilever into contact and force distance curves were recorded. Beginning with a load force of 2 nN the time in contact was varied between 0 s and 60 s and repeated at higher load forces up to 50 nN. For every combination of load force and dwell time three curves were recorded. These experiments were repeated on at least 3 different spots for each of the 13 samples.

To extract adhesive interaction forces the recorded raw data where transformed to quantitative force versus deformation curves by accounting for the spring constant, lever sensitivity, and the lever deflection in contact.[7-8] From these curves, the adhesion force (maximum restoring force during retraction out of contact) is extracted and converted to the work of adhesion according to the procedure described in 5.17. All recorded data were processed and are shown in section 5.17. Only certain individual measurements were excluded when there were experimental errors or the evaluation model could not be applied (no fit match could be determined).

4. Compound Characterization

4.1 U_1^C – AKPSSPPTYKGGGC

MALDI-TOF-MS

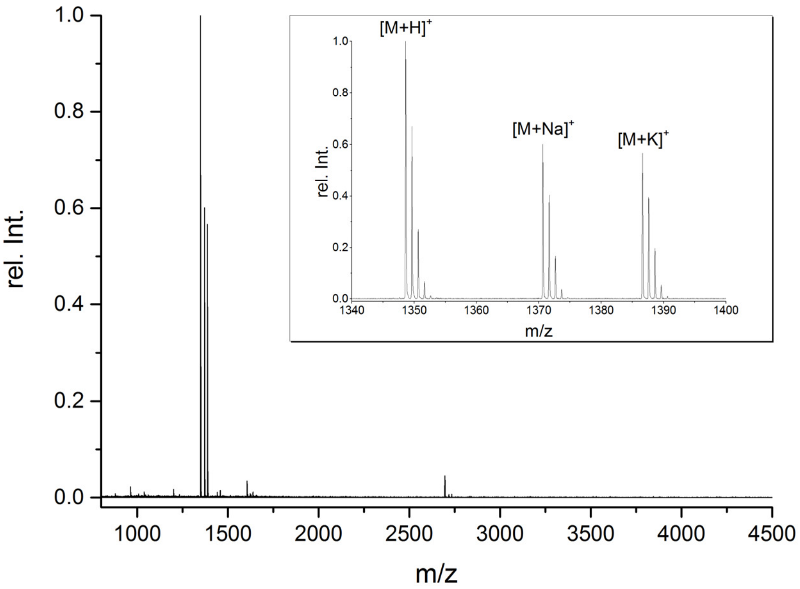

calculated: M = 1347.66 Da; found: $[M+H]^+$ = 1348.65 Da, $[M+Na]^+$ = 1370.65 Da, $[M+K]^+$ = 1386.63 Da.

UPLC-UV/VIS-QMS

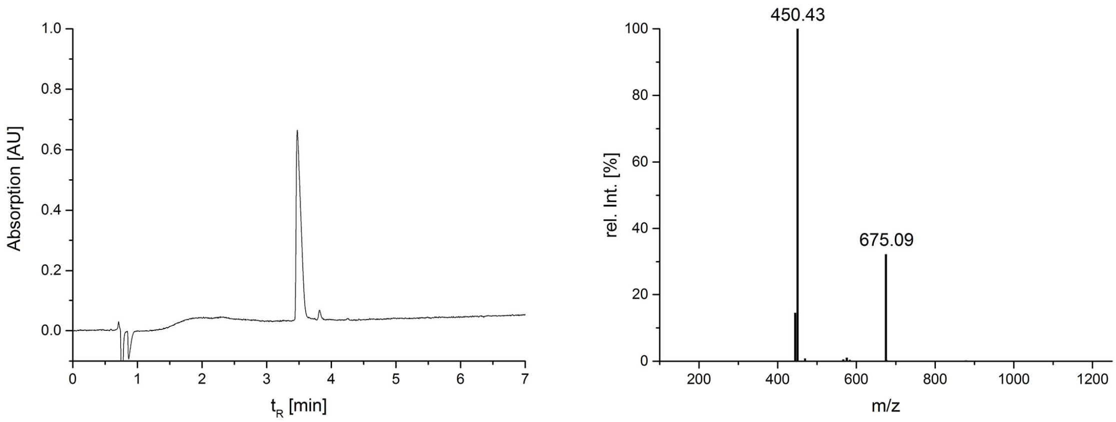

Solvent A: MQ-water, 0.1% FA, Solvent B: acetonitrile, 0.1% FA, gradient 5-23% B (7 min). UV/VIS: t_R = 3.47 min, 97.5% purity.

ESI-QMS: calculated: $[M+2H]^{2+}$ = 675.28, $[M+3H]^{3+}$ = 450.52; found: $[M+2H]^{2+}$ = 675.09, $[M+3H]^{3+}$ = 450.43.

4.2 U_2^C – AKPSYPPPTYKGGGC

MALDI-TOF-MS

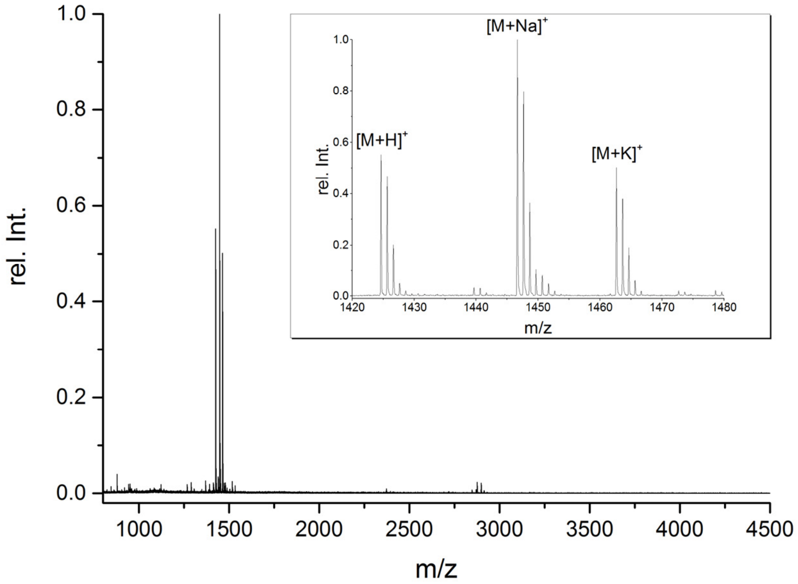

calculated: M = 1423.69 Da, found: $[M+H]^+$ = 1424.65 Da, $[M+Na]^+$ = 1446.65 Da, $[M+K]^+$ = 1462.63 Da.

UPLC-UV/VIS-QMS

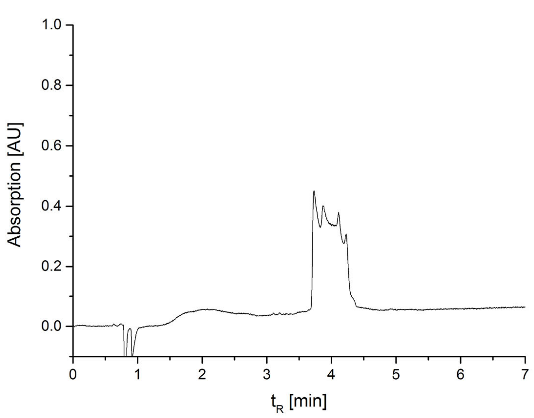

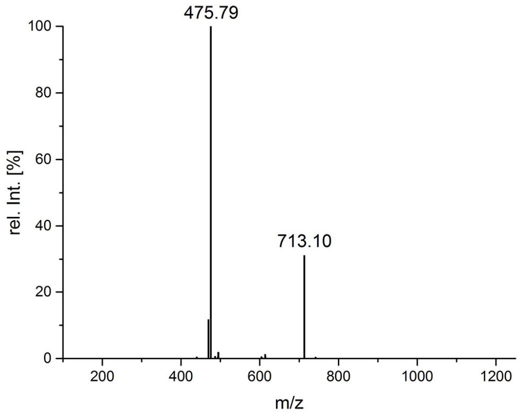

Solvent A: MQ-water, 0.1% FA, Solvent B: acetonitrile, 0.1% FA, gradient 5-30% B (7 min).
UV/VIS: t_R = 3.69 – 4.38 min, 100.0% purity.
ESI-QMS: calculated: $[M+2H]^{2+}$ = 713.33, $[M+3H]^{3+}$ = 475.89; found: $[M+2H]^{2+}$ = 713.10, $[M+3H]^{3+}$ = 475.79.

4.3 U_1^S – AKPSSPPTYKGGGS

MALDI-TOF-MS

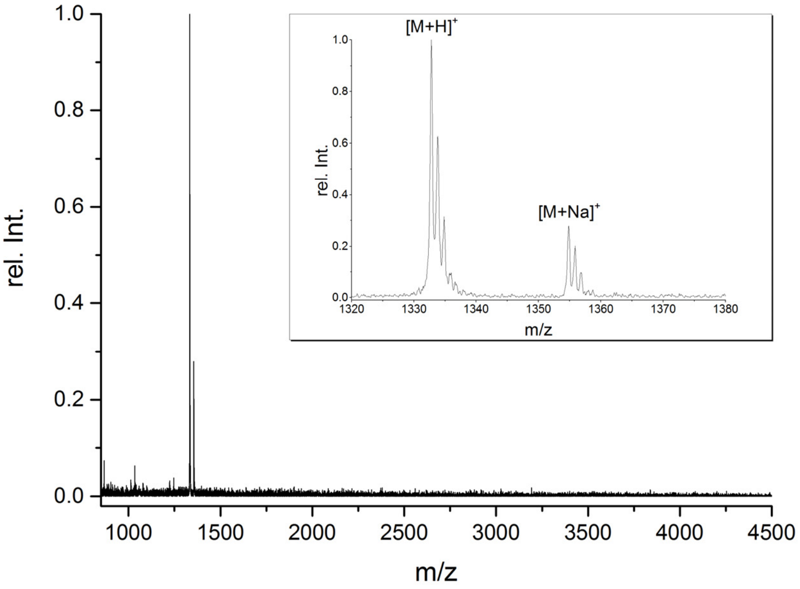

calculated: M = 1331.68 Da, found: $[M+H]^+$ = 1332.81 Da, $[M+Na]^+$ = 1354.83 Da.

UPLC-UV/VIS-QMS

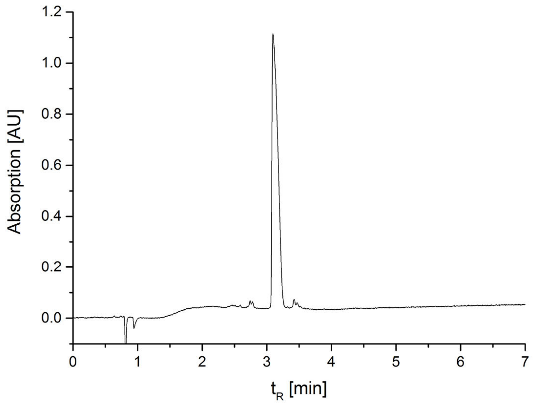

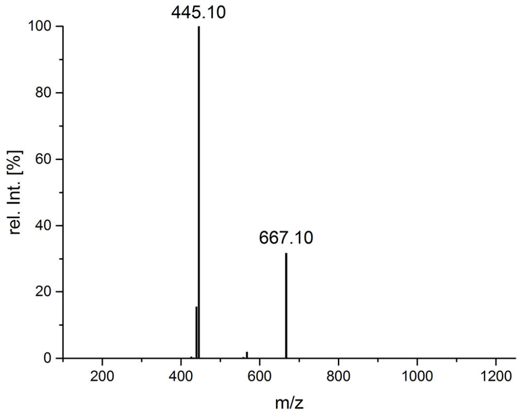

Solvent A: MQ-water, 0.1% FA, Solvent B: acetonitrile, 0.1% FA, gradient 5-23% B (7 min).
UV/VIS: t_R = 3.10 min, 97.4% purity.
ESI-QMS: calculated: $[M+2H]^{2+}$ = 667.25, $[M+3H]^{3+}$ = 445.18; found: $[M+2H]^{2+}$ = 667.10, $[M+3H]^{3+}$ = 445.10.

4.4 U_{1N}^{C} – PTF(4-NO_2)KGGGC

MALDI-TOF-MS

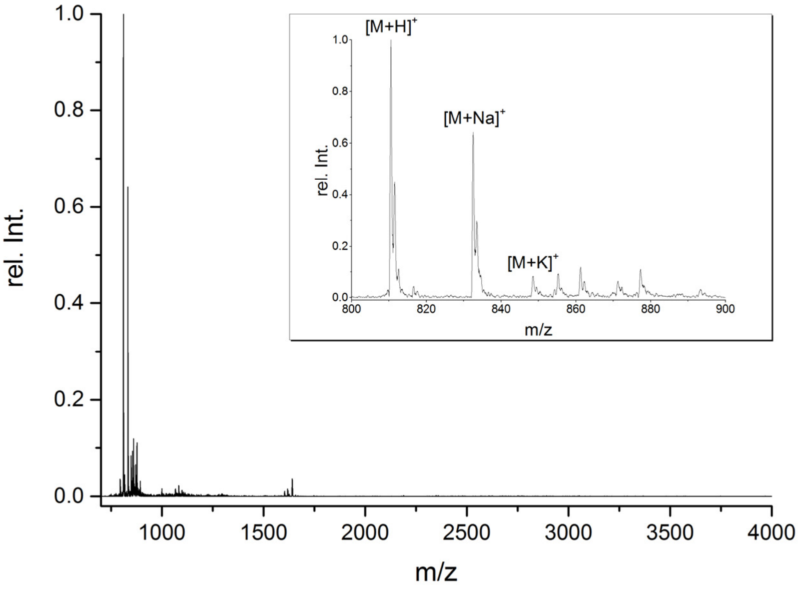

calculated: M = 809.35 Da, found: $[M+H]^+$ = 810.52 Da, $[M+Na]^+$ = 832.52 Da, $[M+K]^+$ = 848.53 Da.

UPLC-UV/VIS-QMS

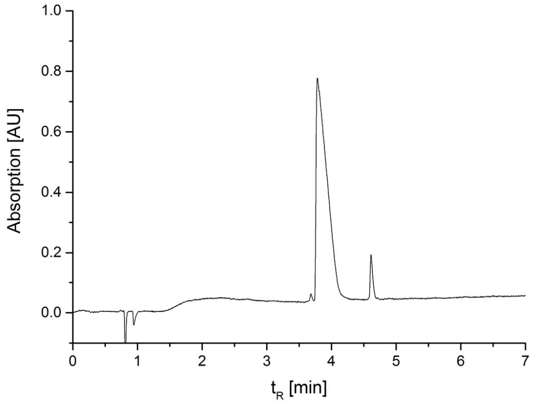

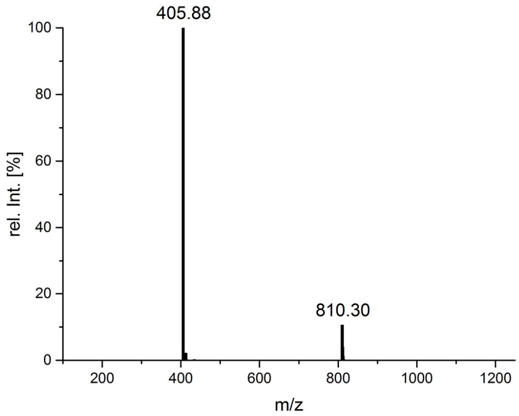

Solvent A: MQ-water, 0.1% FA, Solvent B: acetonitrile, 0.1% FA, gradient 5-23% B (7 min).
UV/VIS: t_R = 3.05 min, 94.7% purity.
ESI-QMS: calculated: $[M+H]^+$ = 810.91, $[M+2H]^{2+}$ = 405.96; found: $[M+H]^+$ = 810.30, $[M+2H]^{2+}$ = 405.88.

5. Experiments

5.1 MALDI-TOF polymerization kinetics of polyU$_1^C$

A solution of U$_1^C$ was activated according to protocol given in 3.4 at a 100 µL scale. 10 µL samples were taken after different reaction times and were immediately frozen in liquid nitrogen. Figure S1 shows measurements performed using CHCA matrix. After 5 min complete oxidation of tyrosine residues from U$_1^C$ to Dopa (+16 Da) and Dopaquinone (+14 Da) could be observed. Also a shift of +12 Da appeared which corresponds to an intramolecular addition reaction of thiol or amine to Dopaquinone or to a tautomerization of Dopaquinone to α,β-Dehydrodopa[9] and subsequent oxidation. Figure S2 shows polymerization of U$_1^C$ after 10 min with a DP of up to 21 measured using DHB matrix.

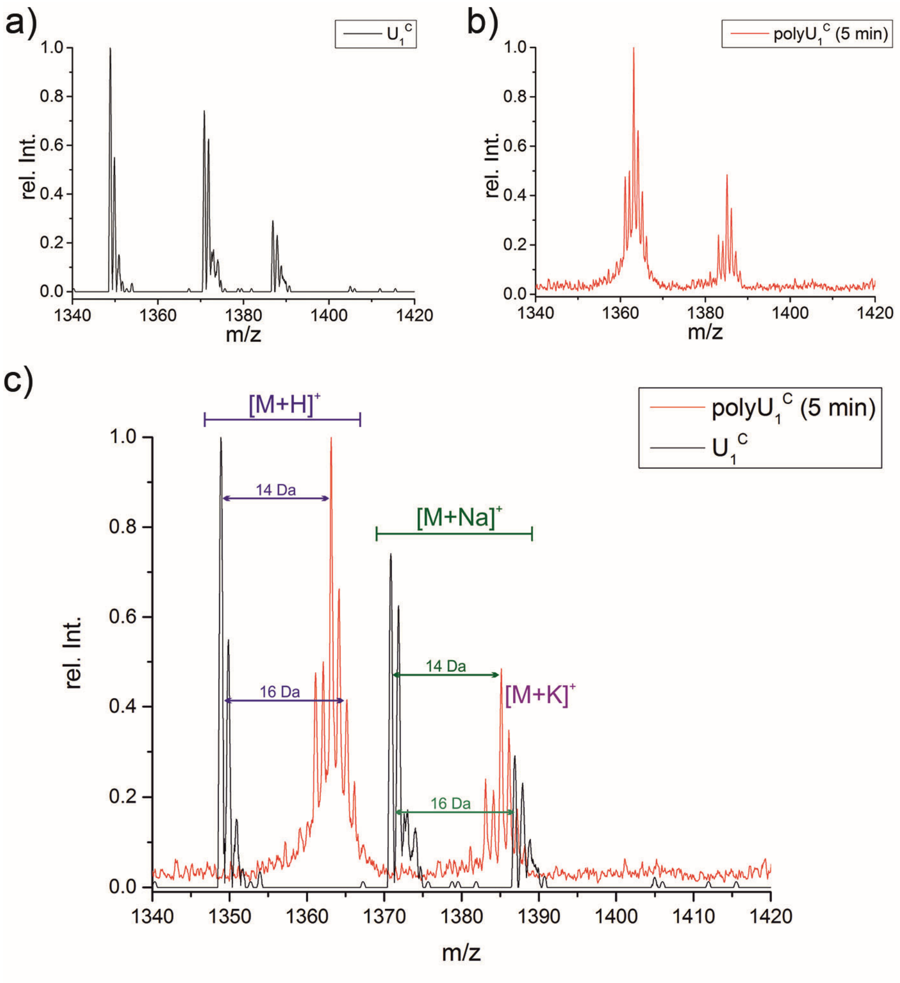

Figure S1. MALDI-TOF-MS measurement of U$_1^C$. a) before (black) and b) 5 min after enzymatic oxidation (red). c) Comparison of spectra before and after oxidation with indicated mass shifts.

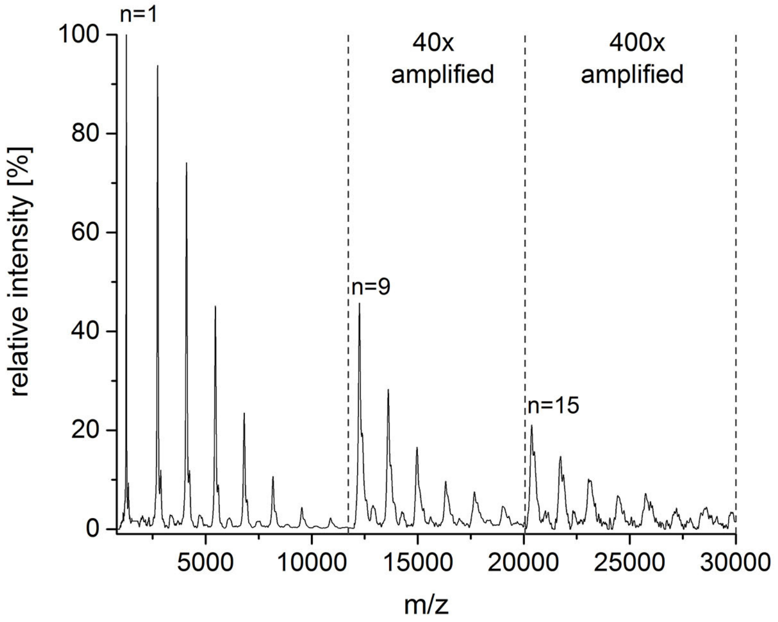

Figure S2. MALDI-TOF-MS spectrum of polyU$_1^C$ 10 min after enzymatic oxidation.

The MALDI-TOF-MS spectrum of polyU$_1^C$ was combined from three separately measured spectra, indicated by the dotted lines. Gating/deflection was used for mass areas m/z > 12000 to reduce saturation effects of the time of flight detector by matrix components or lower mass polymers. Optimal conditions were found for gating/deflection of m/z 2000 below the selected mass areas (m/z = 12000-20000 and m/z = 20000-30000). Signal intensities of the higher mass areas were amplified for better visualization.

5.2 MALDI-TOF polymerization kinetics of polyU$_2^C$

A solution of U$_2^C$ was activated according to protocol given in 3.4 at a 100 µL scale. 10 µL samples were taken after different reaction times and immediately frozen in liquid nitrogen. Measurements performed using CHCA matrix are shown in Figure S3. After 5 min complete oxidation of tyrosine residues from U$_2^C$ could be observed. A mixture of one oxidized tyrosine residue to Dopa (+16 Da) or Dopaquinone (+14 Da) and two oxidized tyrosine residues to Dopa/Dopa (+32 Da), Dopa/Dopaquinone (+30 Da) and Dopaquinone/Dopaquinone (+28 Da) was detected. The low signal to noise ratio after oxidation indicates a low concentration of lower order multimers measured in MALDI-TOF-MS and therefore a rapid polymerization process. Figure S4 shows the MALDI-TOF-MS spectrum measured using DHB matrix after 10 min of U$_2^C$ polymerization with a DP of up to 17.

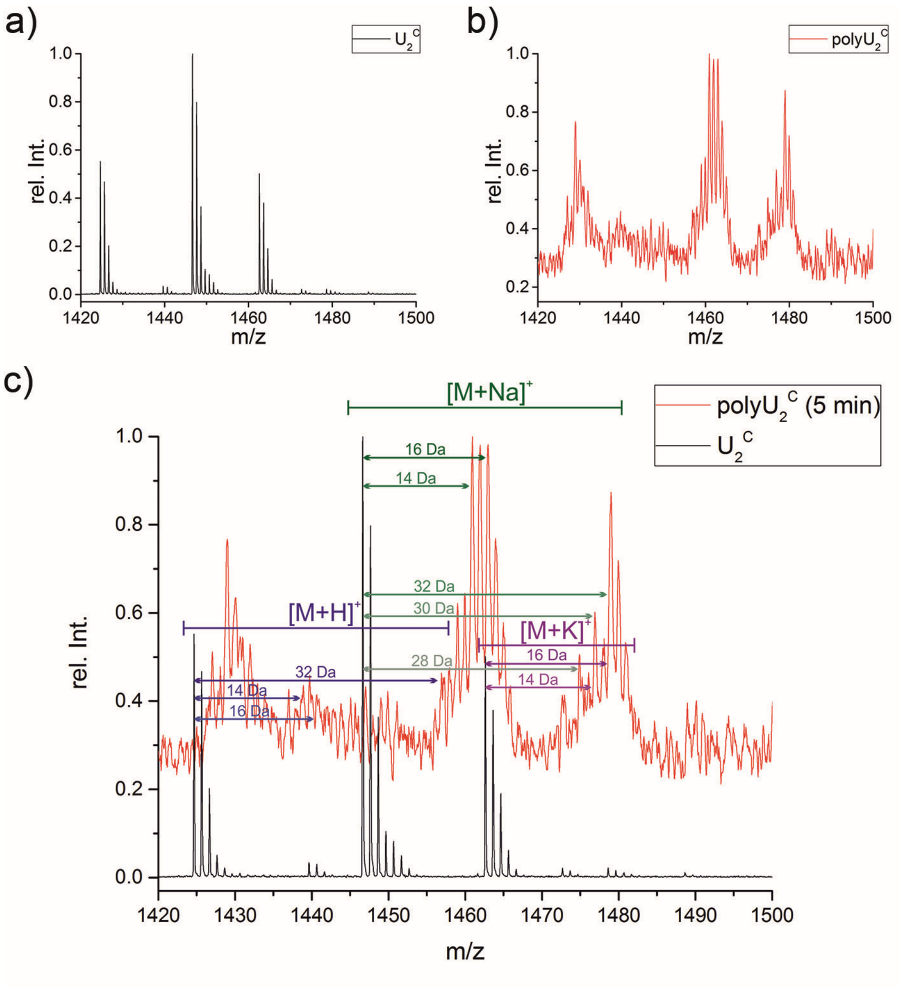

Figure S3. MALDI-TOF-MS measurement of U$_2^C$. a) before (black) and b) 5 min after enzymatic oxidation (red). c) Comparison of spectra before and after oxidation with indicated mass shifts.

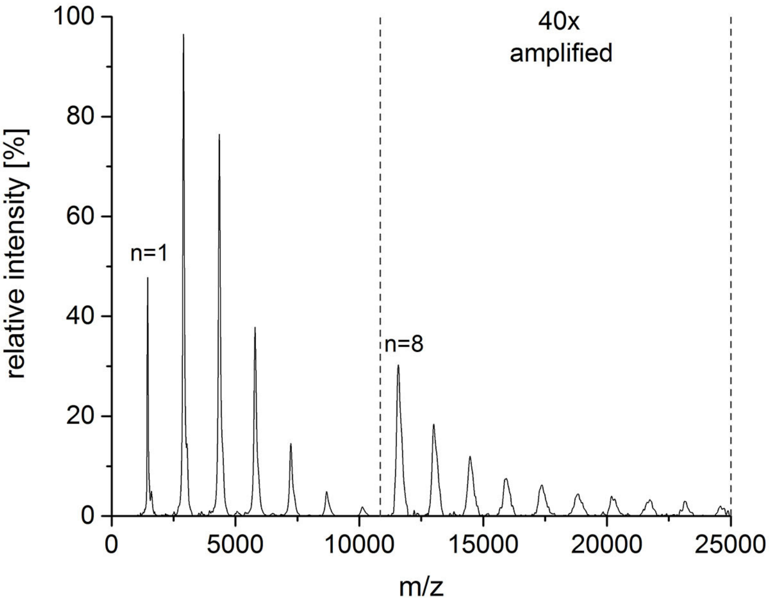

Figure S4. MALDI-TOF-MS spectrum of polyU$_2^C$ 10 min after enzymatic oxidation.

The MALDI-TOF-MS spectrum of polyU$_2^C$ was combined from two separately measured spectra, indicated by the dotted lines. Gating/deflection was used for the mass area m/z > 11000 to reduce saturation effects of the time of flight detector by matrix components or lower mass polymers. Optimal conditions were found for gating/deflection of m/z 2000 below the selected mass area (m/z = 11000-25000). Signal intensities of the higher mass area were amplified for better visualization.

5.3 SDS PAGE polymerization kinetics for U_1^C

A 0.25 mM solution of U_1^C was polymerized according to protocol given in 3.4 using 100 U/mL *Ab*PPO4 tyrosinase. Samples of 30 µL were taken after different reaction times and 0.5 µL of 3.6 M HCl were added (resulting in pH 2 of the sample solution) to stop the enzymatic reaction. Initial samples were taken from reaction mixture after ~5 seconds. Subsequently the samples were frozen in liquid nitrogen and stored at -20 °C until SDS PAGE measurement. 15 µL of each reaction sample and an *Ab*PPO4 tyrosinase reference were loaded onto the gel for measurement.

Figure S5 shows immediate formation of multimers ranging from ~10 kDa to ~25 kDa in apparent molecular weight. After 10 min reaction time the average molecular weight shifts to slightly higher values and an intense band at ~20 kDa is observed. Further, no significant changes in molecular weight distribution are observed. This indicates a rapid polymerization process that leads to high yields after several minutes at an *Ab*PPO4 concentration of 100 U/mL. Additionally stained area is visible at the bottom of the wells, which is not the case for the enzyme reference or the protein ladder. This indicates formation of high molecular weight material that can not be resolved in the polymer matrix due to its molecular weight cutoff.

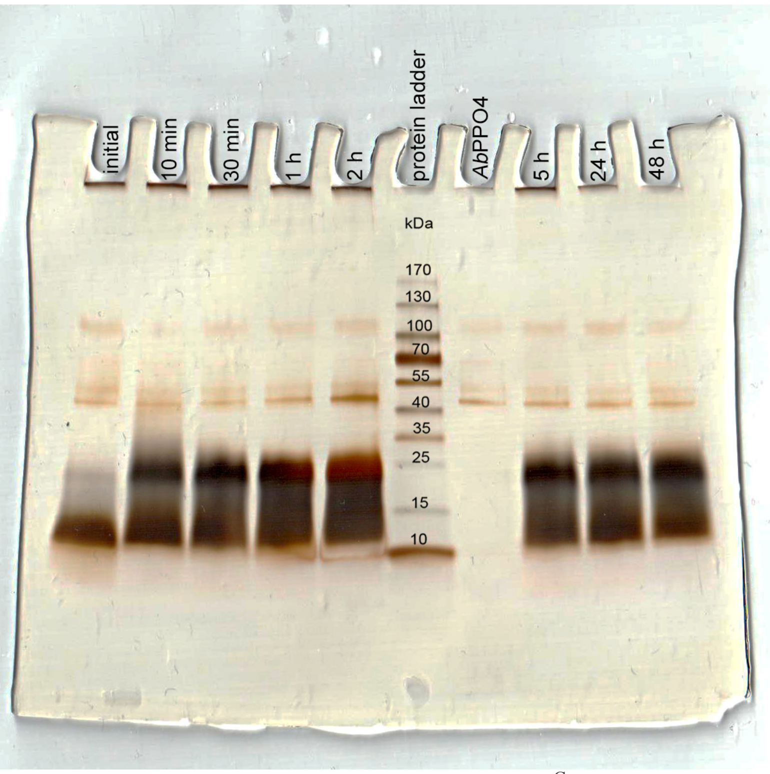

Figure S5. SDS PAGE measurement of polyU_1^C, 100 U/mL *Ab*PPO4 tyrosinase.

5.4 SDS PAGE polymerization kinetics for U_2^C

A 0.25 mM solution of U_2^C was polymerized according to protocol given in 3.4 using 100 U/mL *Ab*PPO4 tyrosinase. Samples of 30 µL were taken after different reaction times and 0.5 µL of 3.6 M HCl were added (resulting in pH 2 of the sample solution) to stop the enzymatic reaction. Initial samples were taken from reaction mixture after ~5 seconds. Subsequently the samples were frozen in liquid nitrogen and stored at -20 °C until SDS PAGE measurement. 15 µL of each reaction sample and an *Ab*PPO4 tyrosinase reference were loaded onto the gel for measurement.

The same trend for the polymerization kinetics as for polyU_1^C can be observed. Figure S6 shows again immediate polymerization of U_2^C. Compared to polyU_1^C higher masses are formed in polyU_2^C. In the first lane, a band which extends over the entire mass range is visible along with a stained area at the bottom of well. For subsequent lanes a mass range is formed for polyU_2^C ranging from ~10 to ~100 kDa in apparent molecular weight with the most intense band coloring at ~20 kDa. Again no significant change after 10 min of reaction time can be observed. The pronounced staining at the well bottom at 10 min and later indicates cross-linking and formation of material unable to penetrate the gel.

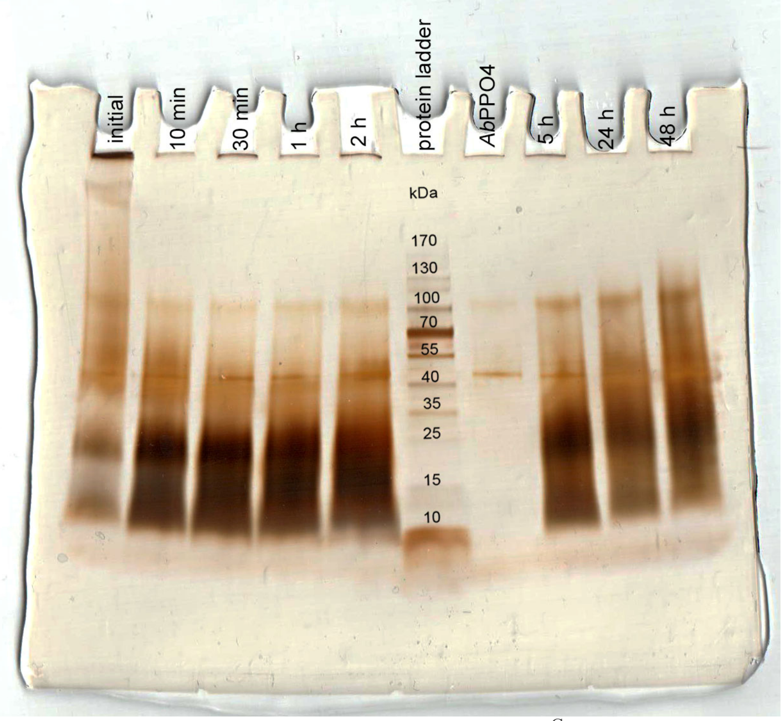

Figure S6. SDS PAGE measurement of polyU_2^C, 100 U/mL *Ab*PPO4 tyrosinase.

5.5 SDS PAGE unimer zero reference

SDS PAGE of non polymerized unimer samples in the same concentration as used for SDS PAGE experiments 5.3 and 5.4 (15 µL sample from 0.25 mM solution) was performed as zero time reference samples. As no bands are visible it is shown that multimerization of unimers starts immediately but only after addition of enzyme (initial samples in 5.3 and 5.4).

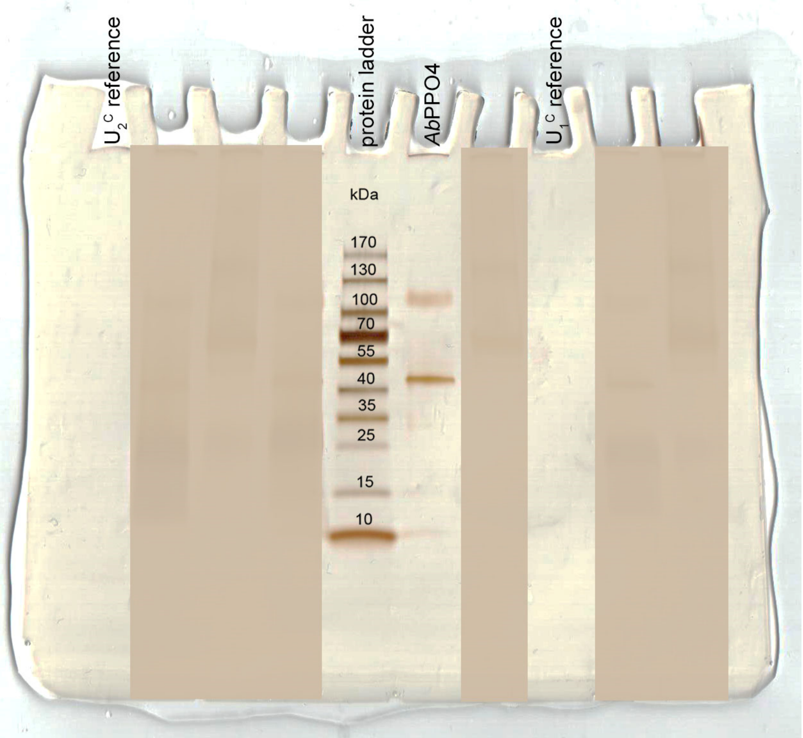

Figure S7. SDS PAGE measurement of unimers U_1^C (right) and U_2^C (left) as zero time reference in the same concentration as polymer samples in 5.3-4. Lanes with a dark overlay contain irrelevant samples.

5.6 GPC kinetic measurements of polyU$_1^C$

A 0.75 µM solution of U$_1^C$ was polymerized according to protocol given in 3.4 using an *Ab*PPO4 tyrosinase concentration of 50 U/mL. After different reaction times samples of 1.5 mL were taken and 13.5 µL of 6 M HCl were added (resulting in pH 2 of the sample solution). Afterwards samples were frozen in liquid nitrogen and lyophilized. For GPC measurement the lyophilized samples were completely redissolved in 2 mL Milli-Q water yielding 0.75 mg/mL polymer. Two molecular weight fractions are visible after 10 min reaction time. The low molecular weight fraction at 9-11 mL has a peak molecular weight of M_P=19.4 kg/mol (cf. Table S1). This is in accordance with SDS PAGE measurements (see 4.3) where a main band at ~20 kDa was observed. The high molecular weight fraction at 6-9 mL has a peak molecular weight of M_P=530 kg/mol (cf. Table S1) and is therefore exceeding the resolvable mass range in SDS PAGE. This explains the visible bands at the bottom of the gel wells and confirms the existence of higher molecular weight material being formed during polymerization. Elugrams of the aqueous GPC show a system peak and a salt peak at retention volumes of 11.4 and 13.2 mL, respectively that are omnipresent and characteristic to the system.

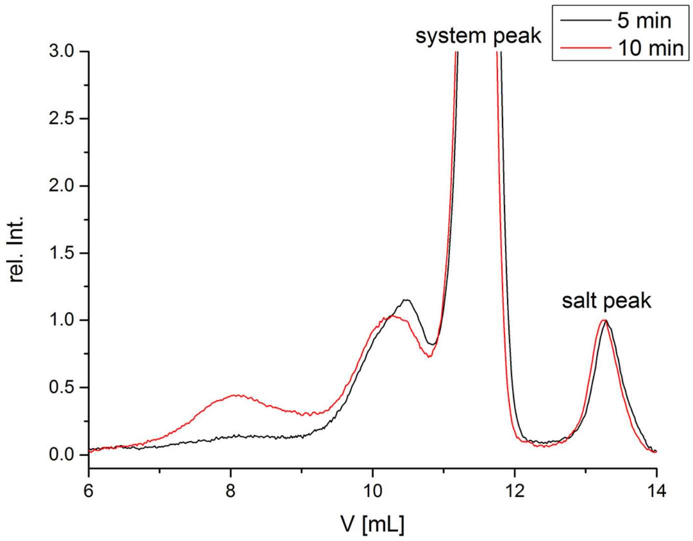

Figure S8. GPC kinetic measurements of polyU1C after different reaction times.

Table S1. Average apparent molecular weight (M_P) of each fraction of the GPC measurements.

t	M_P app.(fraction1) [g/mol]	M_P app.(fraction2) [g/mol]
**5 min**	14 400	-
**10 min**	19 400	530 000

GPC reference measurements

A reference experiment to investigate unimer consumption was performed using UPLC. A 0.75 µmol/mL solution of U_1^C was enzymatically activated using 50 U/mL of *Ab*PPO4 according to protocol given in 3.4. Figure S9 shows the chromatogram of the polymerization mixture after 10 min reaction time. The unimer peak at 3.41 min disappeared in the polymer sample, thus suggesting quantitative conversion of U_1^C.

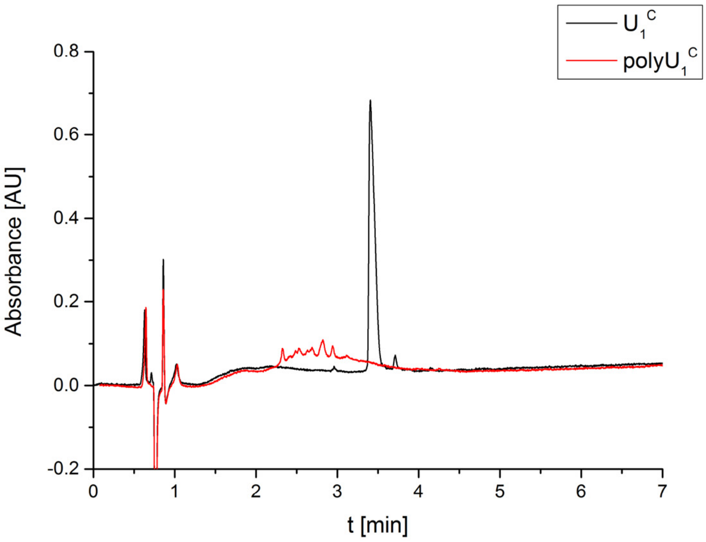

Figure S9. UPLC chromatograms of U_1^C unimer and polyU_1^C after 10 min reaction time.

5.7 SDS PAGE measurement of activated U_1^S

A 0.25 µM solution of U_1^S was activated according to protocol given in 3.4 using 100 U/mL *Ab*PPO4 tyrosinase. A reference unimer solution (0.25 µM) and *Ab*PPO4 tyrosinase solution were loaded onto the gel along with the sample after 1 h reaction time. Neither the unimer sample nor the activated U_1^S sample show any bands (besides the *Ab*PPO4 tyrosinase band at 44 kDa). Hence, no polymerization took place for the unimer U_1^S that contains no cysteine and a polymerization mechanism relying on other than cysteine and Dopa can be ruled out. Unimer mass of U_1^S (M=1332.5 Da) alone is too low to form distinct bands in the gel and is likely washed out of the gel during the staining process.

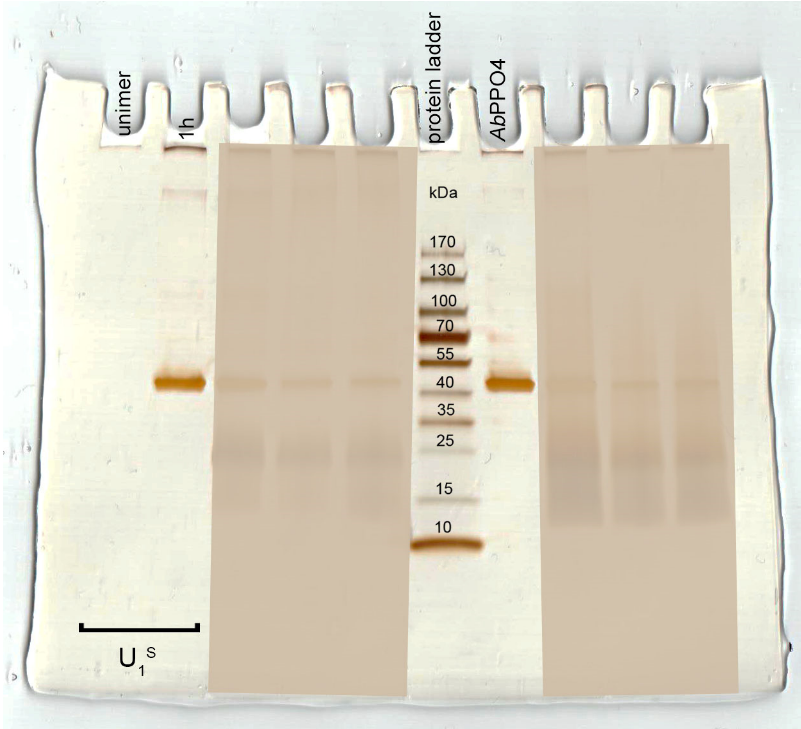

Figure S10. SDS PAGE measurement of U_1^S unimer and activated U_1^S after 1 h of reaction time. Lanes with a dark overlay contain irrelevant samples.

5.8 HPLC kinetic measurements of U_1^S activation

Solutions of 0.25 µM U_1^S were activated according to protocol given in 3.4 using 100 U/mL and 50 U/mL *Ab*PPO4 tyrosinase. Samples of 100 µL were taken after different reaction times, 1.6 µL 6 M HCl were added (resulting in pH 2) to stop the enzymatic reaction and samples were frozen in liquid nitrogen. Samples were measured immediately after thawing.

Since U_1^S contains no cysteine, polymerization does not take place and kinetics of the individual enzymatic activation process can be recorded. Figure S11 compares the conversion rates of the two experiments reaching full conversion after only 1 min for 100 U/mL *Ab*PPO4 and after 10 min for 50 U/mL *Ab*PPO4, respectively. Changes in the chromatogram of both experiments can be seen in Figure S12 and show the same result. Four distinct peaks are formed, while the peak of native U_1^S (15.0/14.7 min) vanishes. ESI-MS measurements of the peaks show all masses of activated unimers species (cf. Figure S13). The first two peaks have a mass corresponding to the unimer activation to Dopaquinone. The third peak appears to be resulting from a mixture of Dopa and Dopaquinone where peaks of the individual species may not be resolved. The mass of the last peak corresponds to Dopaquinone with a mass loss of 2 Da, which was already observed during MALDI-MS measurements (see 5.1/5.2) of polymerization solution and indicates either an intramolecular addition reaction or further oxidation of tautomerized Dopaquinone to α,β-Dehydrodopaquinone[9].

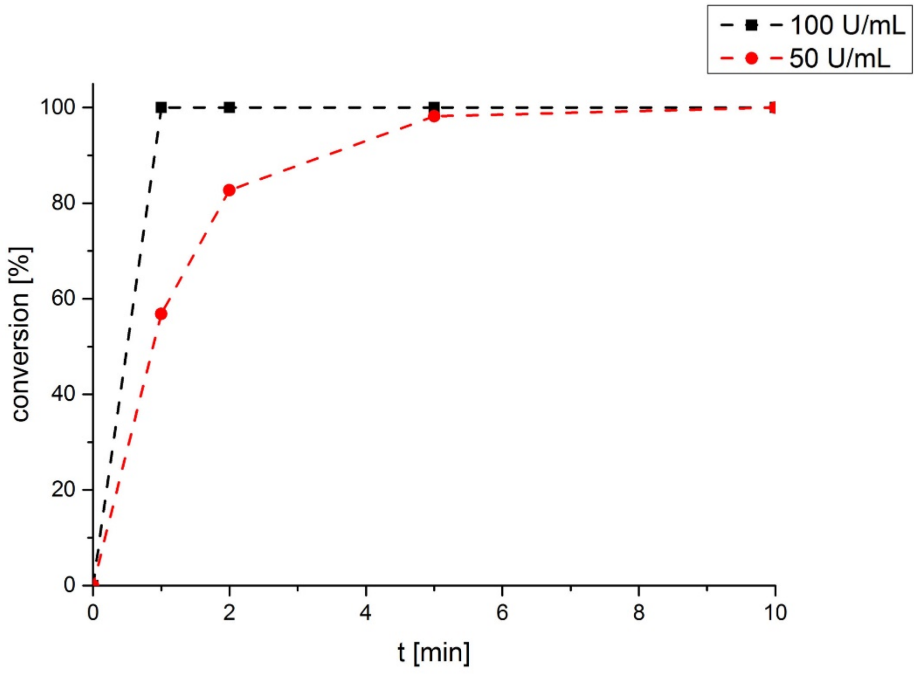

Figure S11. Time resolved conversion of U_1^S to enzymatically activated U_1^{S*} using an *Ab*PPO4 tyrosinase concentration of 100 U/mL (black) and 50 U/mL (red).

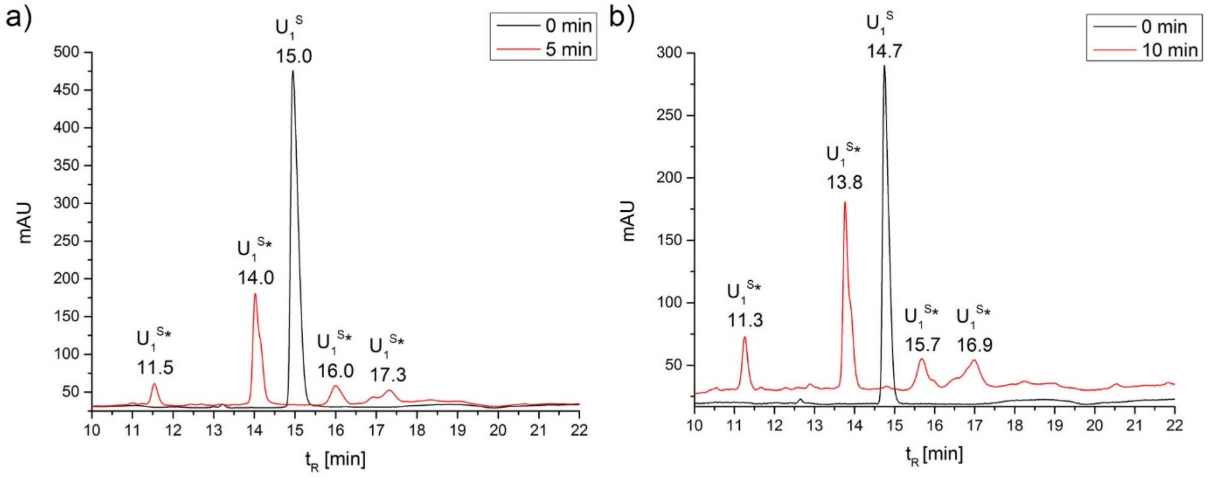

Figure S12. Comparison of HPLC chromatograms at 210 nm of U_1^S before and after activation using an *Ab*PPO4 tyrosinase concentration of a) 100 U/mL and b) 50 U/mL.

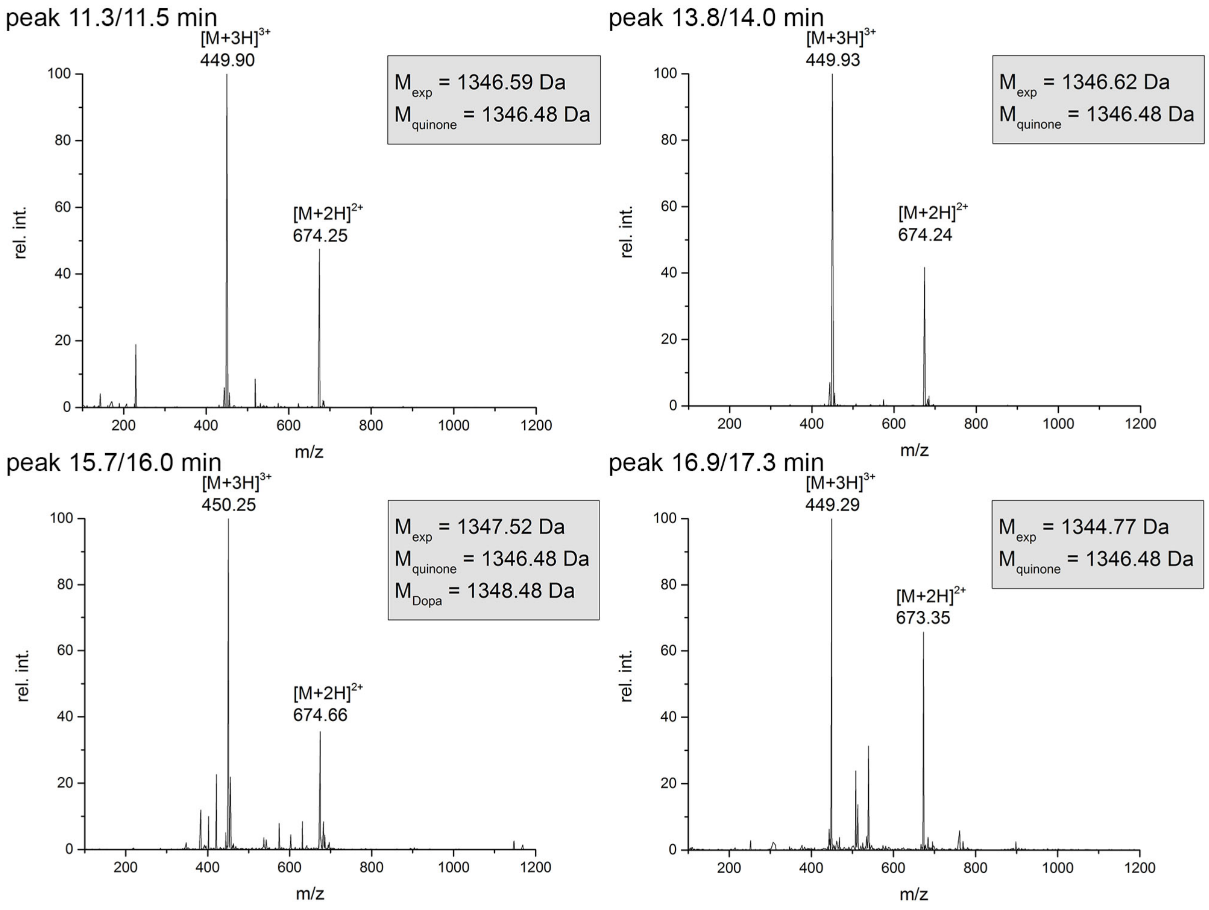

Figure S13. Representative ESI-MS spectra of the 4 peaks from the activated U_1^S chromatogram.

5.9 HPLC kinetic dimerization measurements of the model system

A mixture of U_1^S (0.25 µM) and U_{1N}^C (0.26 µM) in a ratio of 1:1.05 was activated according to protocol given in 3.4 using 50 U/mL *Ab*PPO4 tyrosinase. U_{1N}^C acted as a thiol component and U_1^S as a phenol component. Upon enzymatic activation they form a dimer that is linked via cysteinyldopa bond. Samples of 250 µL were taken after different reaction times, 2.25 µL 6 M HCl were added (resulting in pH 2) to stop the enzymatic reaction and samples were frozen in liquid nitrogen. Samples were measured immediately after thawing.

Quantitative dimerization is observed after 5 – 10 min reaction time. Figure S14 shows a comparison of the HPLC chromatograms of an initial sample and a sample after 10 min of dimerization. The unimer peaks vanish and a set of three distinct peaks is formed (15.7-16.8 min). The mass of all three peaks corresponds to the U_{1N}^C-U_1^S dimer in the oxidative state of cysteinylopa (cf. Figure S15). Ito and Prota *et al*. described that the tyrosinase activated reaction of Dopa with cysteine gives 5-, 2-, and 6-S-cysteinyldopa with a structural isomer distribution of 83%, 16% and 1%, respectively.[10] Consequently, the formation of the three addition products can be assumed and peak area integration suggests product distribution of 82%, 13% and 5% (t_R = 17.0, 15.8 and 16.1 min) to be 5-, 2-, and 6-S-cysteinyldopa linked U_1^S-U_{1N}^C. Surprisingly, the cysteinyldopa linked dimer is quite resistant to oxidation to cysteinyldopaquinone. Although catecholase activity of tyrosinase is much faster than phenolase activity, after 60 min reaction time the majority of the dimer product still remains in the oxidative state of cysteinyldopa. But a progression in oxidation to the cysteinylopaquinone species is visible in the increase of the peak at 17.9 min.

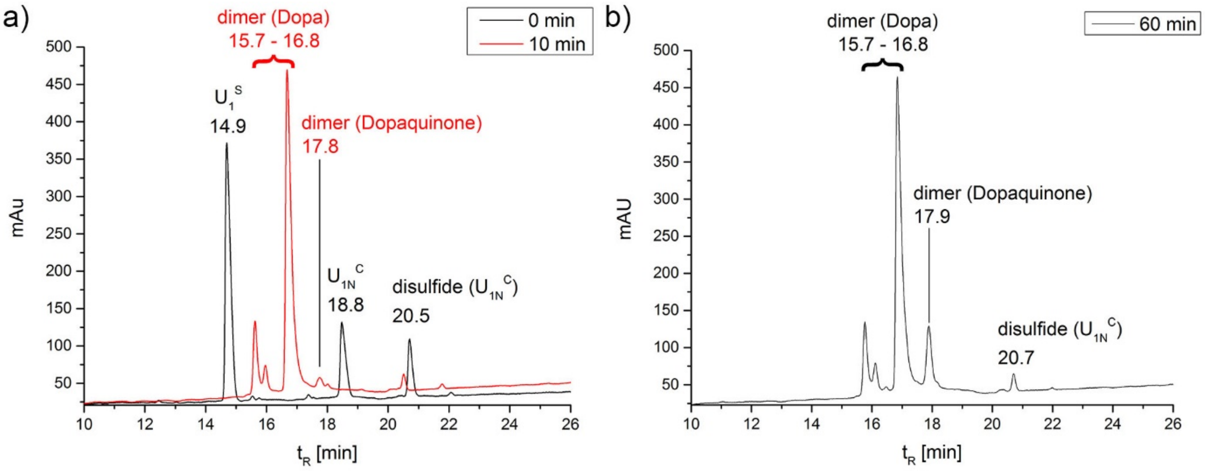

Figure S14. Comparison of chromatograms from HPLC kinetic measurements at 210 nm. a) Chromatograms at initial dimerization and after 10 min of dimerization. b) Chromatogram after 60 min.

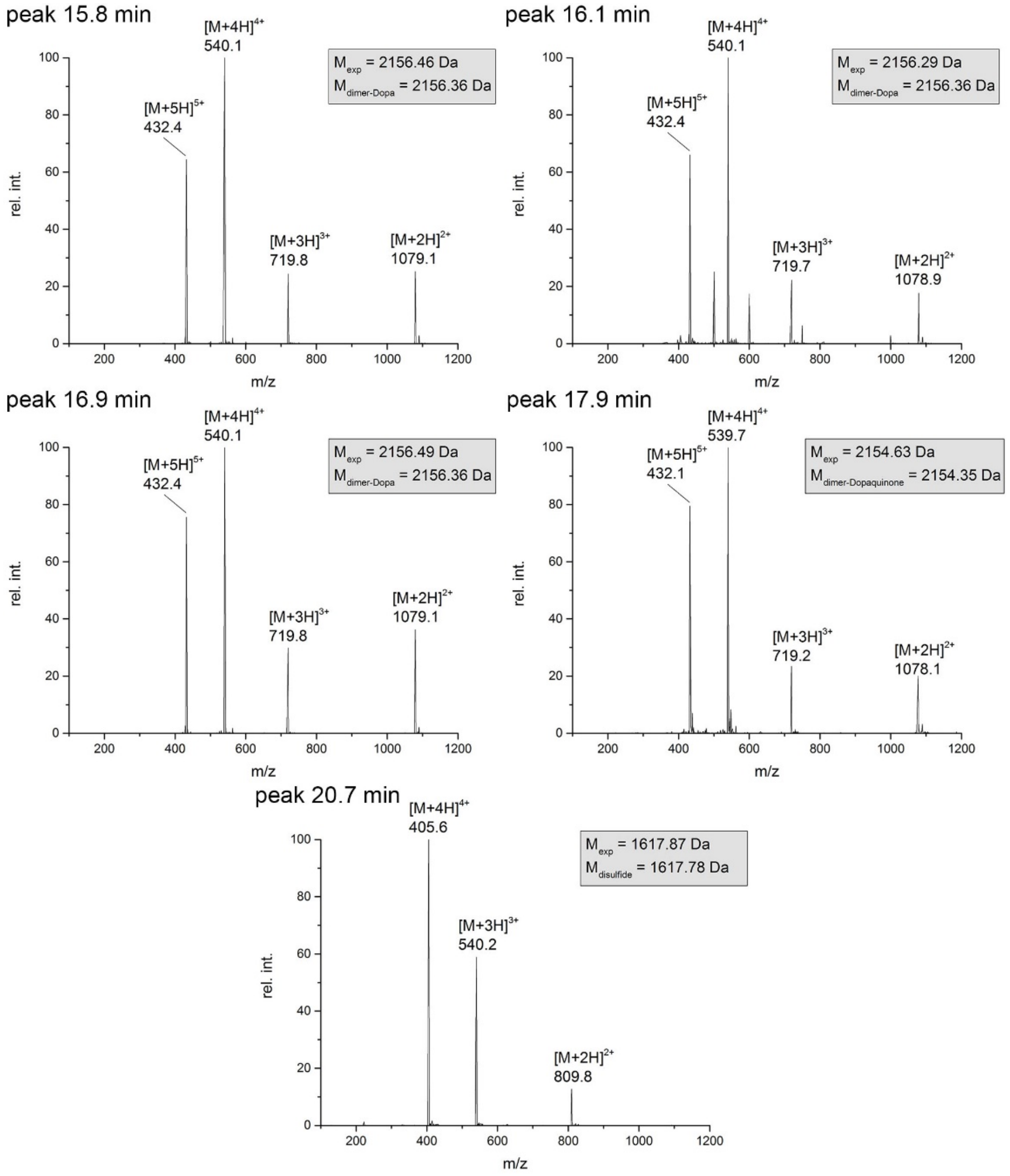

Figure S15. ESI-MS spectra of the chromatogram peaks after dimerization reaction of U_1^S and U_{1N}^C.

5.10 HPLC kinetic measurements of disulfide formation of U_{1N}^{C}

A 0.25 µM solution of U_1^S and U_{1N}^C was shaken over 72 h at 25 °C to determine how fast the autooxidation of the free thiol from U_{1N}^C to disulfide proceeds compared to the dimerization experiment (see 5.8). For kinetic HPLC measurements samples were taken after different reaction times and frozen in liquid nitrogen. Samples were measured immediately after thawing. The results show that the disulfide formation is a very slow process and has a negligible effect as a side reaction in the polymerization. Figure S16 shows that approximately 3% of the U_{1N}^C unimer oxidizes to disulfide within three days.

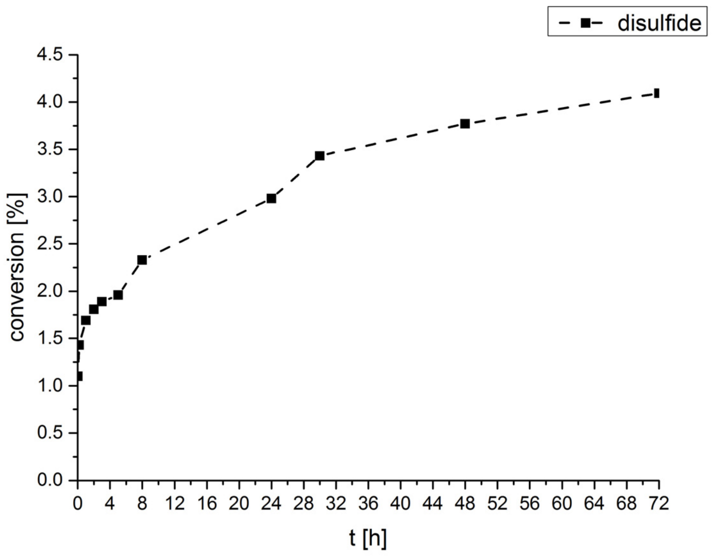

Figure S16. Oxidation kinetics of free thiol from U_{1N}^C to disulfide.

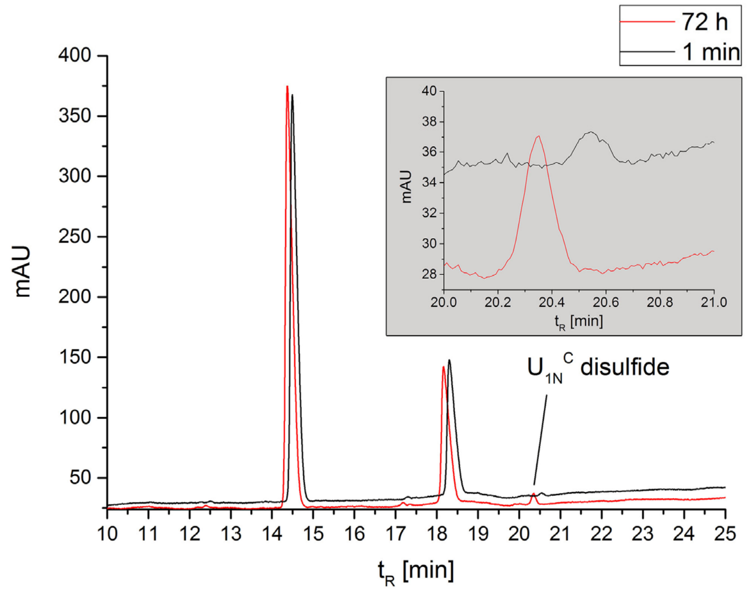

Figure S17. Comparison of HPLC chromatograms from kinetic measurements of the disulfide formation experiment.

5.11 MALDI-TOF-MS/MS measurements of polyU$_1$^{C}

Tandem MS fragmentation spectra were recorded for the dimer of U$_1$^{C} from polymerization reaction. A solution of U$_1$^{C} (0.25 mM) was polymerized for 10 min according to protocol given in 3.4 using 100 U/mL *Ab*PPO4 tyrosinase. 0.9 µL of 6 M HCl were added to 100 µL of polymerization solution to stop the enzymatic reaction and the solution was lyophilized and redissolved in ACN/water (1:1, v/v) + 0.1% TFA for MALDI measurement. The signals corresponding to the dimer were found at m/z 2722.27 - 2726.30 (Figure S19a, inset). In the resulting fragmentation spectrum 57% of the corresponding y- and b-ions for the dimer could be found with an average accuracy of 0.03 ± 0.02 Da (32.1 ppm, cf. Table S2). Several ions (y2-6 to y2-9, b2-10, b2-11) could be found that are characteristic for a cysteinyldopa bonding. Further, mercaptodopaquinone peptidyl fragment **1** and α-methylene peptidyl fragment **2** were observed, resulting from S-C$_\beta$ bond cleavage of the cysteinyldopa linked U$_1$^{C} dimer.

fragment **1** (m/z 1394.62)

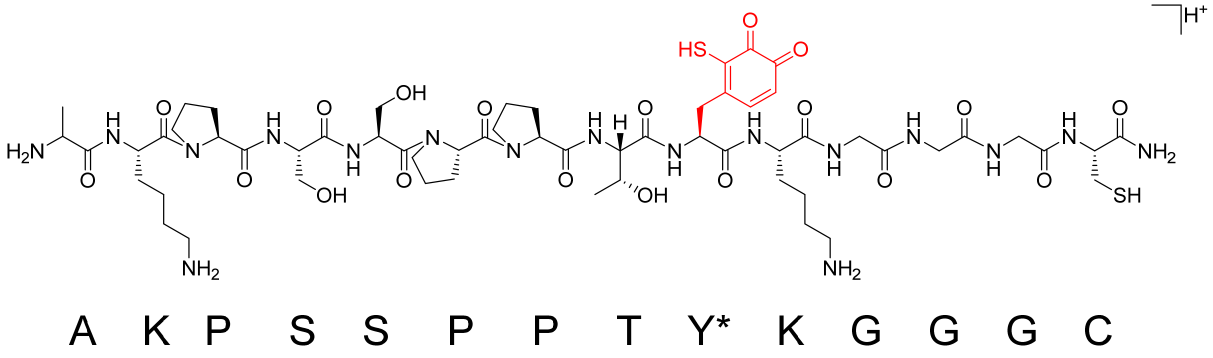

fragment **2** (m/z 1328.66)

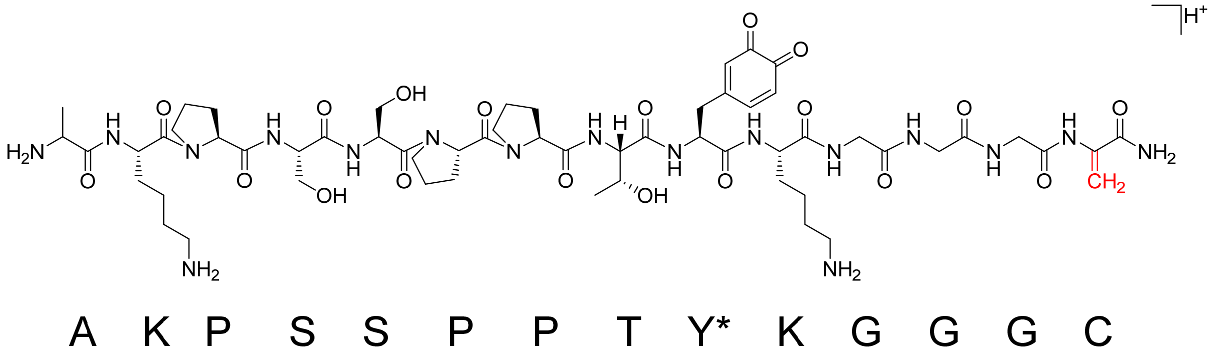

Figure S18. Structures of mercaptodopaquinone peptidyl (**1**) and α-methylene peptidyl (**2**) fragmentation ions.

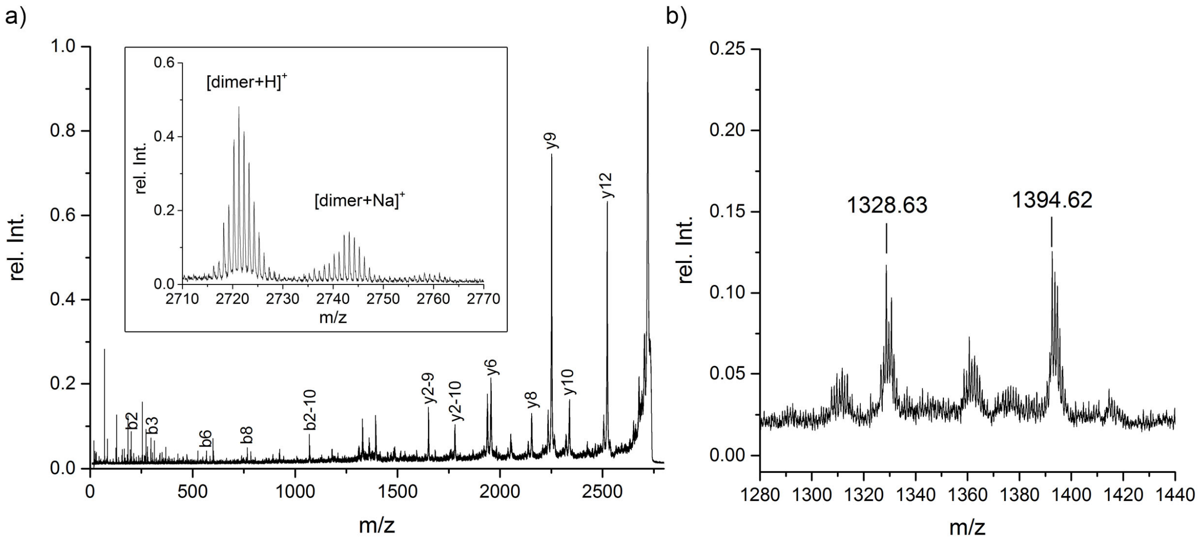

Figure S19. a) MALDI-TOF-MS/MS fragmentation spectrum of the polyU$_1^C$ dimer. Inset shows the parent MS spectrum. b) Close-up of the MALDI-TOF-MS/MS spectrum showing fragmentation ions **1** (m/z 1394.62) and **2** (m/z 1328.63) of the above outlined structures.

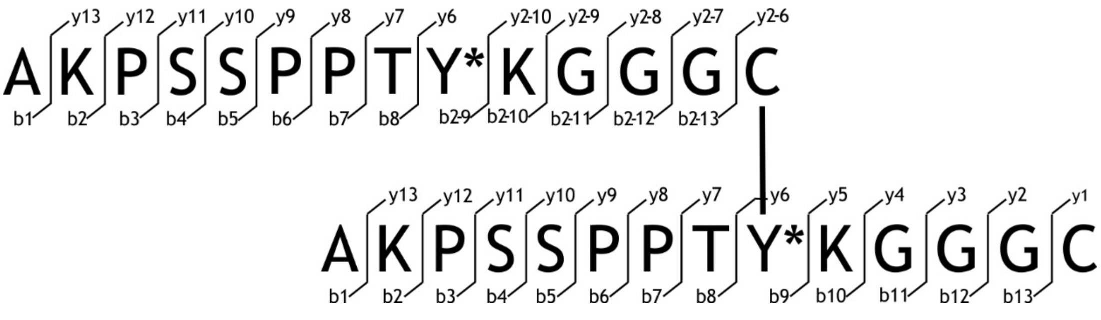

Figure S20. Assignment of the fragmentation ions of the cysteinyldopa linked U_1^C-U_1^C dimer.

Table S2. List of ions found in MALDI-TOF-MS/MS measurement of the dimer mass from polyU_1^C. Green shaded Ions are characteristic for cysteinyldopa cross-link.

ion	calc. [Da]	found [Da]	Δ [ppm]	ion	calc. [Da]	found [Da]	Δ [ppm]
b1	72.044	-		y1	121.043	-	
b2	200.139	200.168	144.9	y2	178.064	-	
b3	297.192	297.228	121.1	y3	235.086	-	
b4	384.224	-		y4	292.107	-	
b5	471.256	471.294	80.6	y5	420.202	-	
b6	568.309	568.358	86.2	y6	1956.869	1956.846	11.8
b7	665.362	665.364	3.0	y7	2057.917	-	
b8	766.409	766.419	13.0	y8	2154.970	2154.980	4.6
b9	2303.076	-		y9	2252.022	2251.982	17.8
b10	2431.171	-		y10	2339.054	2339.001	22.7
b11	2488.193	-		y11	2426.086	2426.039	19.4
b12	2545.214	-		y12	2523.139	2523.095	17.4
b13	2602.235	-		y13	2651.234	2651.181	20.0
b2-9	943.452	943.455	3.2	y2-6	1480.667	1480.697	20.3
b2-10	1071.547	1071.566	17.7	y2-7	1537.689	1537.718	18.9
b2-11	1128.568	1128.561	6.2	y2-8	1594.710	1594.746	22.6
b2-12	1185.590	-		y2-9	1651.732	1651.763	18.8
b2-13	1242.611	-		y2-10	1779.827	1779.834	3.9

5.12 LC-ESI-MS/MS cross-linking experiments

To investigate how secondary cross-linking between polymer chains can occur, a reference experiment was performed where U_1^S (AKPSS**S**^{5}PPT**Y**^{9}KGGG**S**) was enzymatically activated. The lack of cysteine in the unimer sequence does not allow a dimerization or oligomerization via cysteinyldopa, therefore any multimers found have to result from a different cross-linking mechanism. A solution of U_1^S (0.25 mM) was oxidized according to protocol given in 3.4 using 50 U/mL *Ab*PPO4 tyrosinase. After 1 h the reaction was stopped and the sample was submitted to MALDI-TOF-MS, which confirmed the formation of dimerized product with masses between 2685.28 and 2693.34 Da (Figure S21). The sample was then diluted to 20 µM and submitted to LC-ESI-MS/MS using a pikoTip column (75 µm*50 cm, Agilent Poroshell 120 EC-C18) with a gradient of 5.5-20.5% acetonitrile in water (0.1% formic acid) over 30 min (250 nl/min flow at 50 °C). Since the dimer signals with a charge of 2 are superimposed by unimer signals, MS signals with a charge of 3 were analysed. At 22.2, 22.9 and 27.0 min retention time, signals of m/z 897.4465 (Figure S24) were detected corresponding to a U_1^S-U_1^S dimer in the oxidative state of quinone (2689.3096 Da) at z = 3. These ions were fragmented using EThcd with 30% collision energy (Figure S25). In the resulting fragmentation spectrum 44% of the corresponding y-, b- and c-, z-ions for the dimer could be found with an average accuracy of 0.0075 ± 0.0092 Da (4.8 ppm, cf. Table S3). No fragmentation ions were found that would have directly proven the existence of lysinyldopa links. Instead, 5-(3,4-dioxy-1-methylphenyl)dopaquinone peptidyl fragment **3** (Figure S23) was found at m/z 1466.6892 (z=1+2H, Δ=1.5 ppm) and α-hydrogenyl peptidyl fragment **4** was found at m/z 1225.6425 (z=1+H, Δ=1.3 ppm) resulting from a Dopaquinone C_α-C_β bond cleavage of a 5,5'-diDopa linked dimer molecule. This shows that secondary cross-linking besides cysteinyldopa can occur and likely happens via aryloxy coupling instead of lysinyldopa.

A second reference experiment was performed, where U_1^S and U_{1N}^C were enzymatically activated and allowed to dimerize (2159.98 – 2155.01 Da). After further reaction time of up to 48 h samples were submitted to MALDI-TOF-MS and did not show any evidence for formation of tetramers $[U_{1N}^C$-$U_1^S]_2$ or higher order multimers (Figure S22). Hence, it seems that the cysteinyldopa functionality is not susceptible for secondary cross-linking.

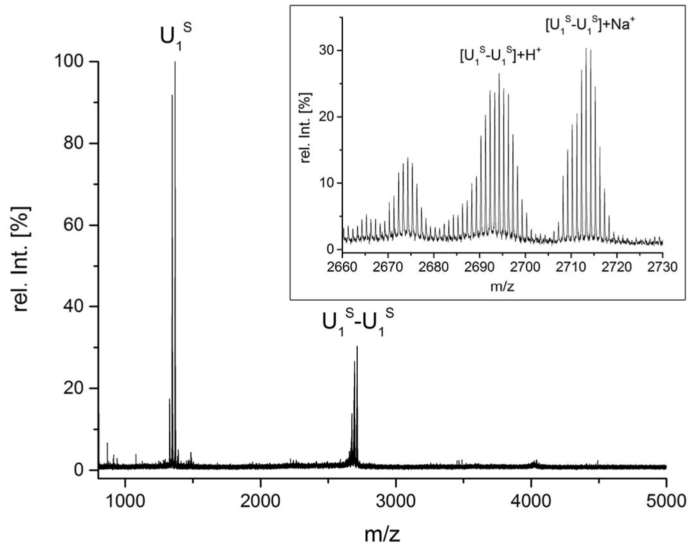

Figure S21. MALDI-TOF-MS spectrum of activated U_1^S after 1 h reaction time, inset shows signals of U_1^{S-} U_1^S dimer.

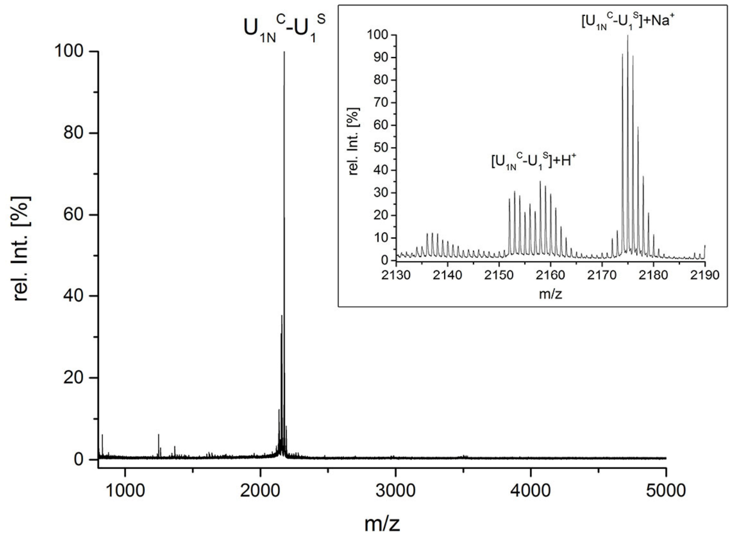

Figure S22. MALDI-TOF-MS spectrum of enzymatic activation of U_1^S in presence of U_{1N}^C after 48 h reaction time, inset shows signals of U_{1N}^C-U_1^S dimer. No further linkage of [U_{1N}^C-U_1^S] dimer units can be observed.

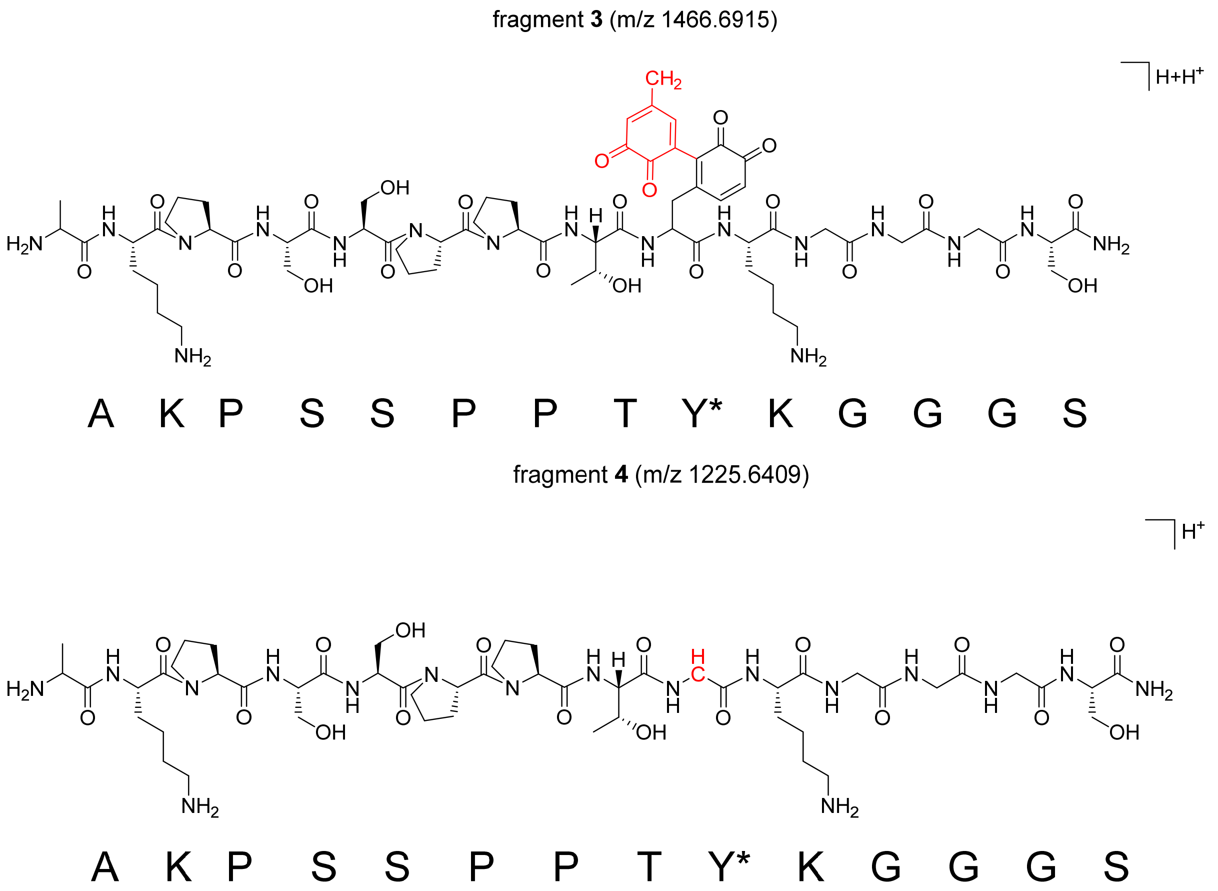

Figure S23. Structures of 5-(3,4-dioxy-1-methylphenyl)dopaquinone peptidyl (**3**) and α-hydrogenyl peptidyl (**4**) fragmentation ions.

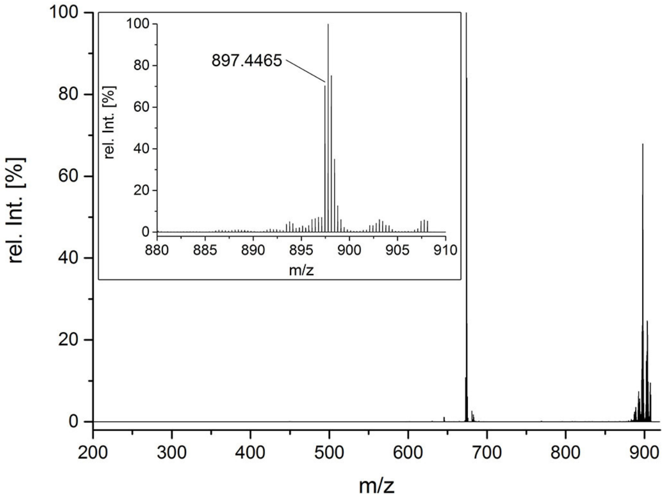

Figure S24. LC-ESI-MS spectrum at t_R 22.9 min.

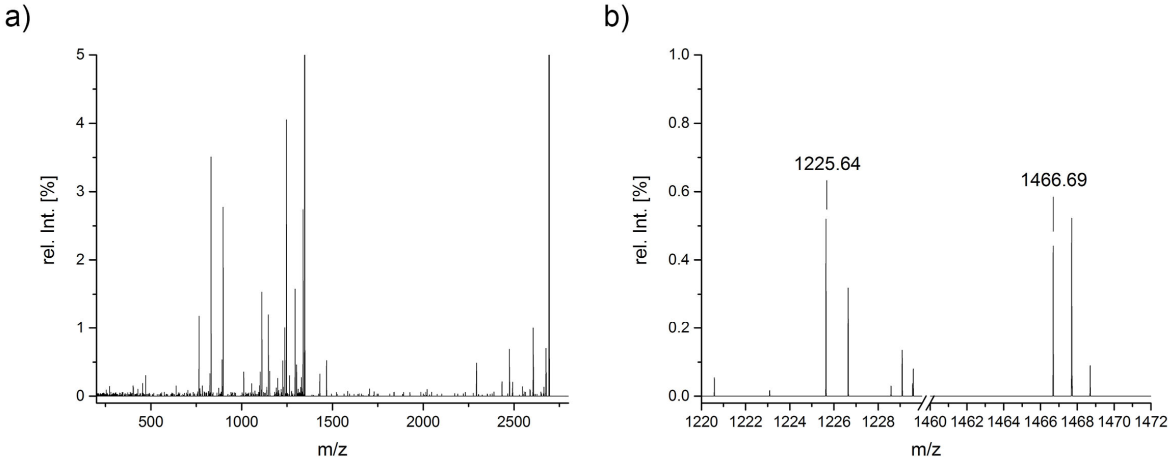

Figure S25. LC-ESI-MS/MS spectrum of U_1^S-U_1^S dimer with m/z 897.4465 parent ion at t_R 22.2 min. a) Full LC-ESI-MS/MS spectrum. b) Close-up of the LC-ESI-MS/MS spectrum showing fragmentation ions **3** (m/z 1466.69) and **4** (m/z 1225.64) of the above outlined structures.

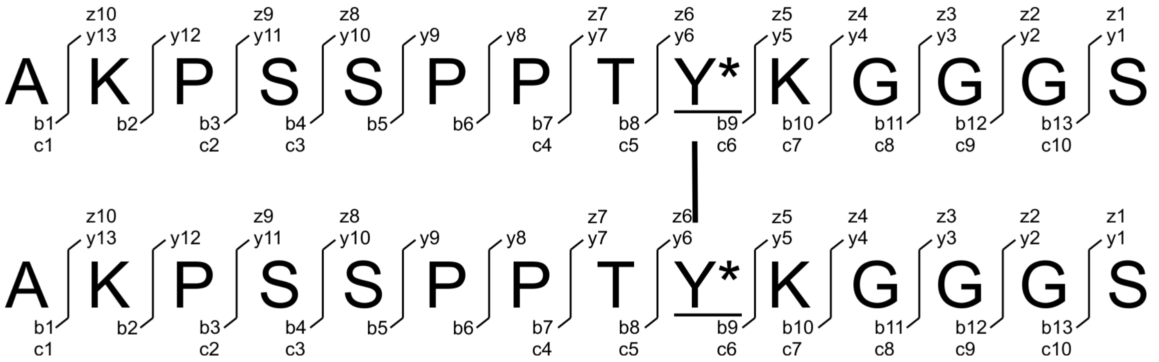

Figure S26. Assignment of the fragmentation ions of the diDopa linked U_1^S-U_1^S dimer.

Table S3. List of ions found in LC-MS/MS measurement of U_1^S-U_1^S dimer with m/z 897.445 at 22.9 min.

ion	charged state	calc. [m/z]	found [m/z]	Δ [ppm]
b2	z=1	200.1394	200.1393	0.3
b5	z=1	471.2562	471.2560	0.5
b9	z=2 (C13)	1144.5553	1144.5508	3.9
b11	z=1+2H (C13)	2475.2354	2475.2288	2.7
b11	z=2 (C13)	1237.1136	1237.0893	19.6
b11	z=3 (C13)	825.0781	825.0622	19.3
b13	z=2+H (C13)	1294.6390	1294.6243	11.4
c3	z=1	401.2507	401.2502	1.2
c5	z=1	783.4359	783.4360	0.1
c6	z=2	1152.5664	1152.5667	0.3
c7	z=1 (C13)	2433.2238	2433.2189	2.0
c9	z=1+2H	2548.2790	2548.2783	0.3
c10	z=1	2603.2849	2603.2708	5.4
y5	z=1	404.2252	404.2248	1.0
y10	z=2	1154.0536	1154.0534	0.2
y11	z=2 (C13)	1198.0714	1198.0698	1.3
y12	z=2	1246.0960	1246.0948	1.0
y12	z=3	831.0664	831.0660	0.5
y13	z=2+H (C13)	1311.1492	1311.1310	13.9
z5	z=1+H	388.2065	388.2061	1.0
z8	z=1+2H	2292.0891	2292.0879	0.5
z8	z=2	1145.5404	1145.5496	8.0
z8	z=3+2H (C13)	765.0357	765.0230	16.6
z9	z=2+H	1189.5603	1189.5685	6.9
z10	z=1+H (C13)	2604.2643	2604.2651	0.3
z10	z=2	1301.6302	1301.6204	7.5

5.13 QCM-D experiments on aluminium oxide surface

For QCM measurements sample concentration (0.75 µmol/mL) was reduced and samples were diluted after reaction prior to measurement with degassed Milli-Q water 1:21 v/v (0.03 µmol/mL). Therefore the citrate buffer (17 mM, pH 6.8) was diluted to 0.8 mM as well for use in equilibration and rinsing steps. All measurements were performed at 100 µL/min flow according to protocol 3.5 on an aluminium oxide coated sensor (QSX 309, Biolin Scientific, Sweden). Changes in frequency (Δf) and energy dissipation (ΔD) were recorded for overtones n = 3,5,7,9,11 and 13. Calculations of adsorbed masses for the polymer coatings were performed with QTools Software (version 3.0.10.286, Biolin Scientific AB, Sweden) by approximation of the recorded data according to the Voight-based model[11]. Up to two overtones were disregarded for calculations if the deviation from the other values was too high.

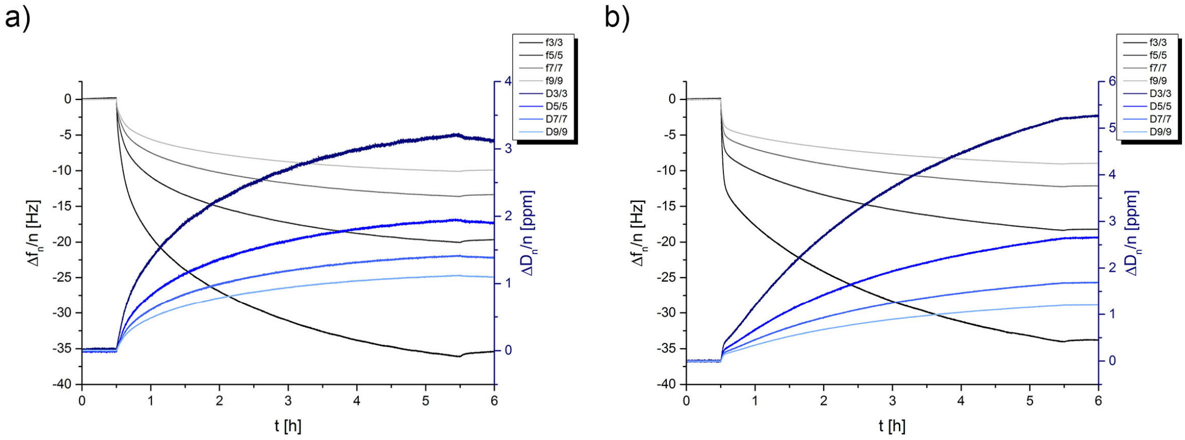

Figure S27. QCM-D response for $\Delta f_n/n$ (black) and $\Delta D_n/n$ (blue) for polyU$_1^C$ (a) and polyU$_2^C$ (b) on aluminium oxide coated sensors.

Figure S27 shows that by dividing frequency and energy dissipation by their corresponding overtones a frequency dependence is visible due to viscoelastic properties of the films. For rigid films with no frequency dependence the measurements would result in identical curves.

5.13.1 polyU$_1^C$ coating

Unimer polyU$_1^C$ was polymerized for 1 h according to protocol given in 3.4 with 0.75 µmol/mL substrate concentration and an *Ab*PPO4 tyrosinase concentration of 50 U/mL. Subsequently, the sample of 1.5 mL was diluted to 33 mL with degassed Milli-Q water and the sensor was incubated for 5 h followed by buffer rinsing. The resulted polyU$_1^C$ showed strong adsorption towards the aluminium oxide surface and during buffer rinsing Δf remained constant, proving the stability of the polymer coating.

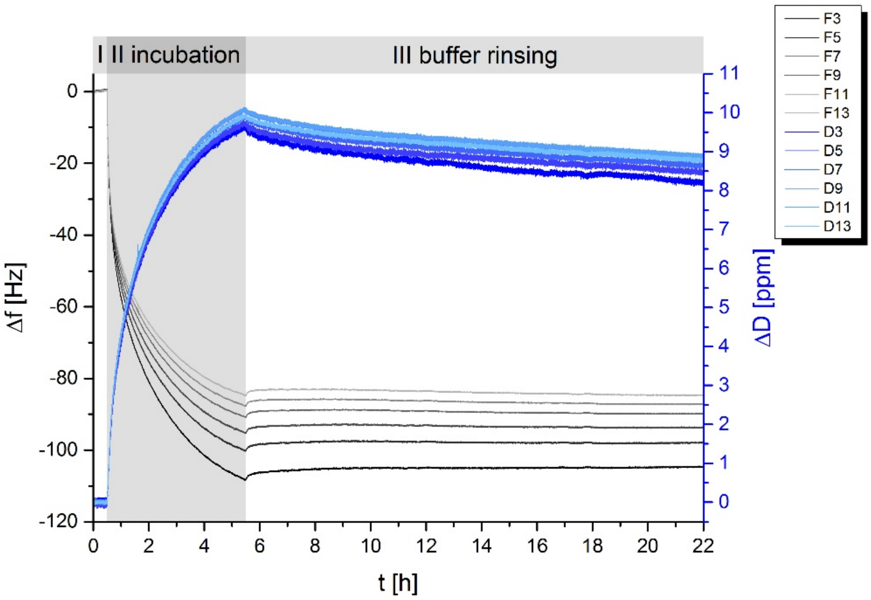

Figure S28. QCM-D adsorption and desorption kinetics of polyU$_1^C$ surface modification on Al_2O_3.

I. citrate buffer solution (0.8 mM, pH 6.8)
II. polyU$_1^C$ solution (34.1 nmol/mL)
III. citrate buffer solution (0.8 mM, pH 6.8)

5.13.2 Rinsing of polyU$_1^C$ coating

To test for coating stability, the polyU$_1^C$ coated sensor was rinsed with 599 mM NaCl solution and 4.2 M hypersaline solution for 1 h. The used hypersaline solution was modeled after salt concentrations of Dead Sea water[12] and contained $MgCl_2$•6H_2O (368.0 g/L, 1.81 mol/L), NaCl (97.0 g/L, 1.66 mol/L), $CaCl_2$•2H_2O (63.2 g/L, 0.43 mol/L), KCl (14.9 g/L, 0.20), NaBr (6.82 g/L, 66.33 mmol/L), Na_2SO_4 (664.7 mg/L, 4.68 mmol/L), $NaHCO_3$ (275.6 mg/L, 3.28 mmol/L). The calculated differences in adsorbed masses amounts to 49.2 ng/cm² (2.4%) for NaCl rinsing and 151.2 ng/cm² (7.2%) for hypersaline rinsing compared to the initial coating, which demonstrates the coating's high stability against salinity.

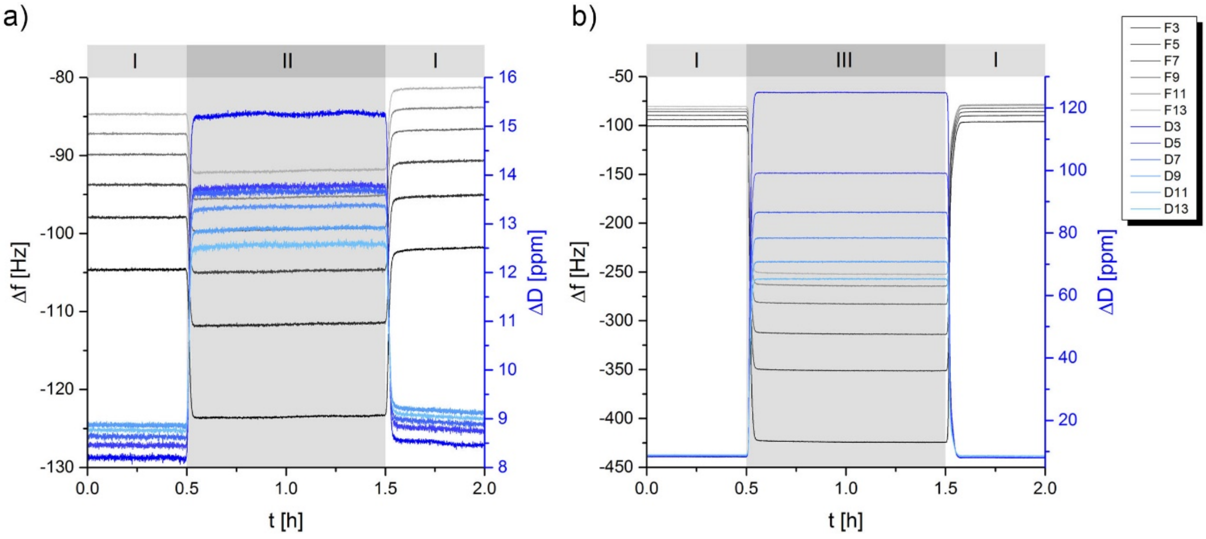

Figure S29. QCM-D adsorption and desorption kinetics of rinsing polyU$_1^C$ modified Al_2O_3 surface with 599 mM NaCl solution (a) and 4.2 M hypersaline solution (b).

I. citrate buffer solution (0.8 mM, pH 6.8)
II. NaCl solution (599 mM)
III. hypersaline solution (4.2 M)

5.13.3 polyU_2^C coating

Unimer polyU_2^C was polymerized for 1 h according to protocol given in 3.4 with 0.75 µmol/mL substrate concentration and an *Ab*PPO4 tyrosinase concentration of 50 U/mL. Subsequently, the sample of 1.5 mL was diluted to 33 mL with degassed Milli-Q water and the sensor was incubated for 5 h followed by buffer rinsing. The resulted polyU_2^C showed strong adsorption towards the aluminium oxide surface. The Δf signal of polyU_2^C decreased further during the buffer rinsing step after coating. Accompanied with an increase in energy dissipation this suggests swelling of the coating and accommodation of solvating water in the polyU_2^C film during buffer rinsing, which is less pronounced for the coatings consisting of polyU_1^C.

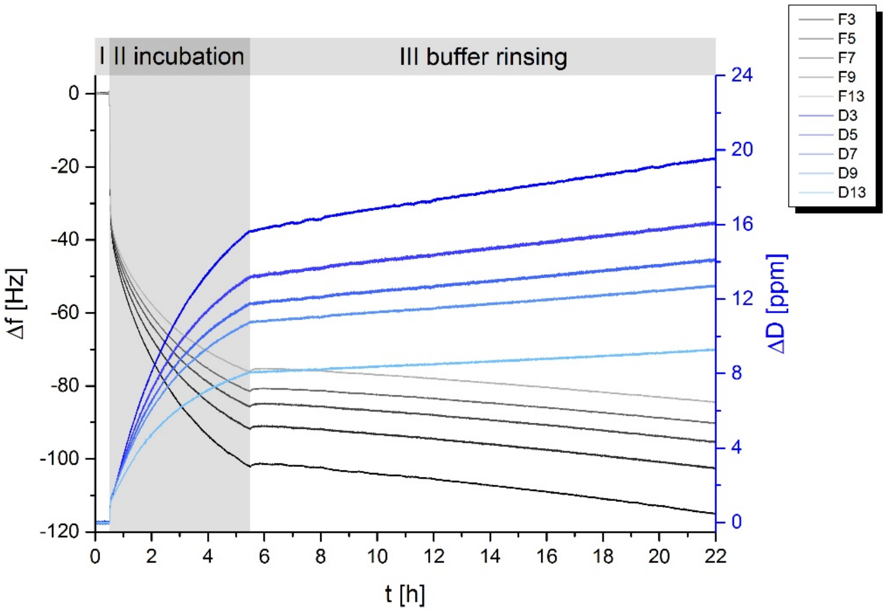

Figure S30. QCM-D adsorption and desorption kinetics of polyU_2^C surface modification on Al_2O_3.

I. citrate buffer solution (0.8 mM, pH 6.8)
II. polyU_2^C solution (34.1 nmol/mL)
III. citrate buffer solution (0.8 mM, pH 6.8)

The Δf signal of polyU_2^C decreased further during the buffer rinsing step after the coating (cf. Fig. S30 step III). Accompanied with an increase in energy dissipation, this suggests swelling of the coating and accommodation of solvating water in the polyU_2^C film during buffer rinsing. This effect is less pronounced for the polyU_1^C coating (cf. Fig. S28 step III), probably because the solvating water content is already higher during the film formation.

5.13.4 Rinsing of polyU$_2^C$ coating

Coating stability of the polyU$_2^C$ coated sensor was tested by rinsing with 599 mM NaCl solution and 4.2 M hypersaline solution for 1 h. The calculated difference in adsorbed masses amounts to 115.2 ng/cm² (3.5%) for NaCl rinsing and 46.8 ng/cm² (1.4%) for hypersaline rinsing compared to the initial coating, which shows high stability of the coating.

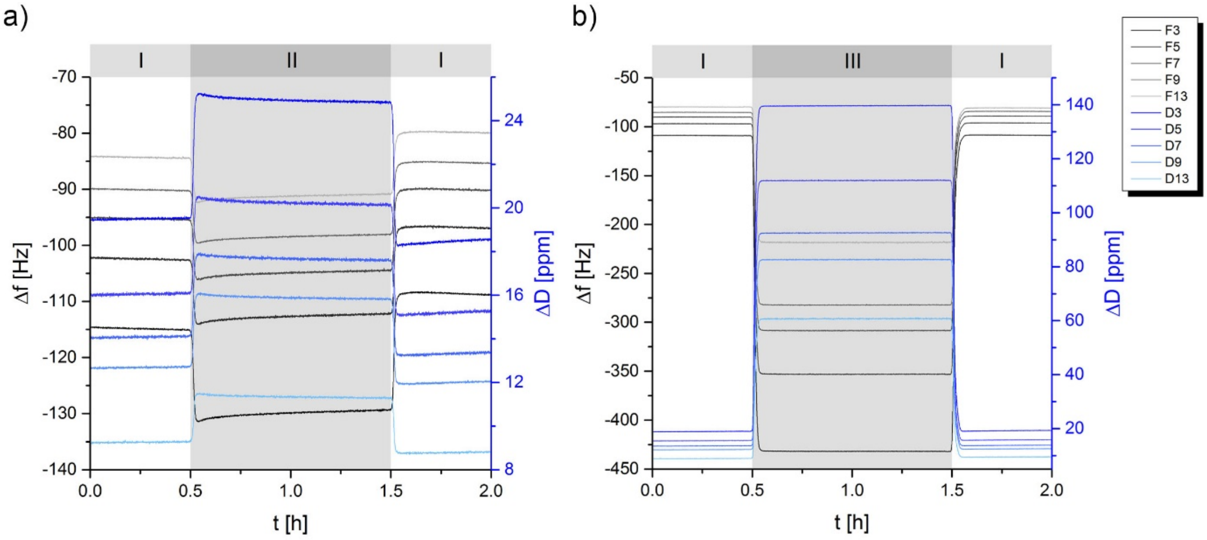

Figure S31. QCM-D adsorption and desorption kinetics of rinsing polyU$_2^C$ modified Al_2O_3 surface with 599 mM NaCl solution (a) and 4.2 M hypersaline solution (b).

I. citrate buffer solution (0.8 mM, pH 6.8)
II. NaCl solution (599 mM)
III. hypersaline solution (4.2 M)

5.13.5 Unimer U_1^C control

As a control experiment QCM-D measurement of the pure unimer was carried out. Therefore, a solution of U_1^C (0.75 µmol/mL) was prepared, the sample of 1.5 mL was diluted to 33 mL with degassed Milli-Q water and the sensor was incubated for 5 h followed by buffer rinsing. Only minor adsorption was observed for the unimer (Figure S32a) which was fully abolished upon rinsing with 599 mM NaCl solution (Figure S32b).

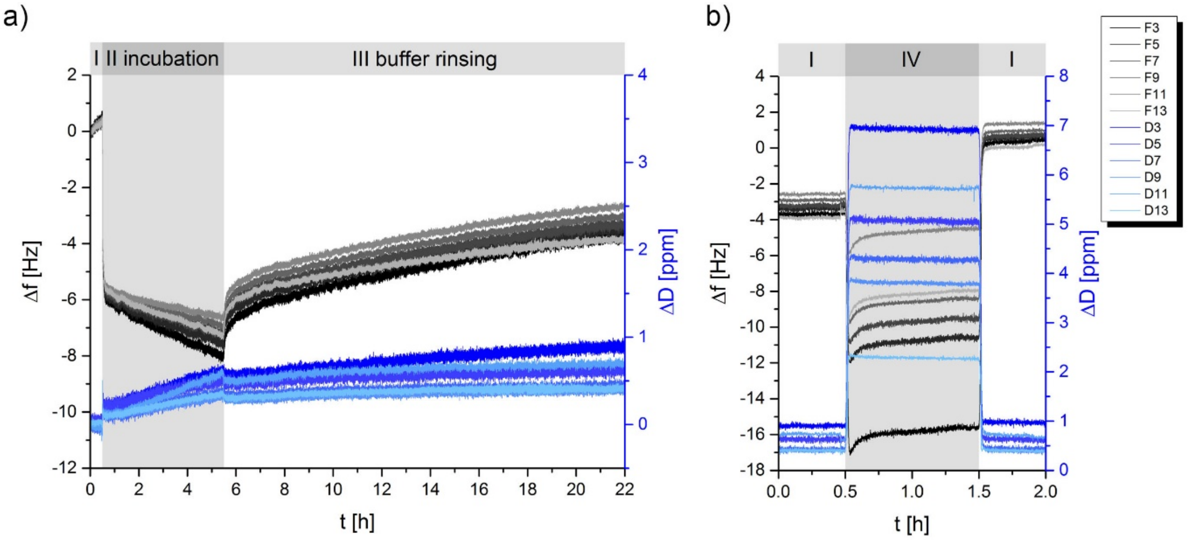

Figure S32. QCM-D adsorption and desorption kinetics of U_1^C surface modification on Al_2O_3. a) Incubation of U_1^C and buffer rinsing. b) Rinsing of U_1^C modified Al_2O_3 surface with NaCl solution (599 mM).

I. citrate buffer solution (0.8 mM, pH 6.8)
II. unimer U_1^C solution (34.1 nmol/mL)
III. citrate buffer solution (0.8 mM, pH 6.8)
IV. NaCl solution (599 mM)

5.13.6 Unimer U_2^C control

QCM-D measurement of the pure unimer was carried out as a control experiment. A solution of U_2^C (0.75 µmol/mL) was prepared, the sample of 1.5 mL was diluted to 33 mL with degassed Milli-Q water and the sensor was incubated for 5 h followed by buffer rinsing. Only minor adsorption was observed for the unimer (Figure S33a). Rinsing the U_2^C coated aluminium oxide sensor with NaCl solution (599 mM) resulted in partial removal of approximately 37% of the adsorbed substance (Figure S33b).

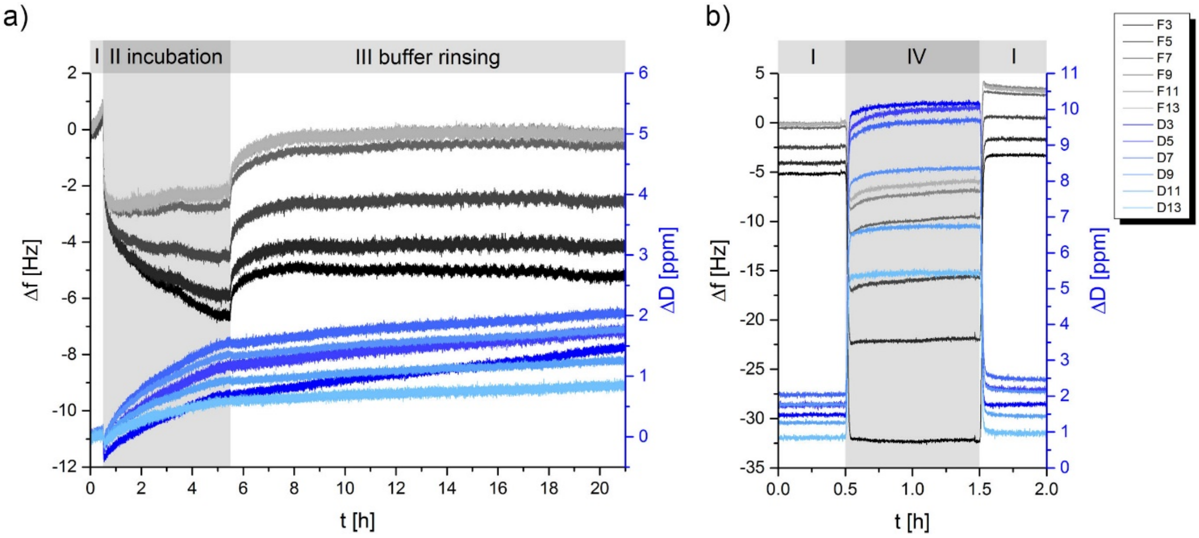

Figure S33. QCM-D adsorption and desorption kinetics of U_2^C surface modification on Al_2O_3. a) Incubation of U_2^C and buffer rinsing. b) Rinsing of U_2^C modified Al_2O_3 surface with NaCl solution (599 mM).

I. citrate buffer solution (0.8 mM, pH 6.8)
II. unimer U_2^C solution (34.1 nmol/mL)
III. citrate buffer solution (0.8 mM, pH 6.8)
IV. NaCl solution (599 mM)

5.13.7 *Ab*PPO4 tyrosinase control

As a control experiment, QCM-D measurement of pure *Ab*PPO4 was performed. Therefore, a solution of *Ab*PPO4 tyrosinase (50 U/mL) was prepared, that contained the same enzyme concentration as in the polymerization mixture. The sample of 1.5 mL was diluted to 33 mL with degassed Milli-Q water and the sensor was incubated for 5 h followed by buffer rinsing. Only minor adsorption was observed, that was almost completely washed off during the buffer rinsing step.

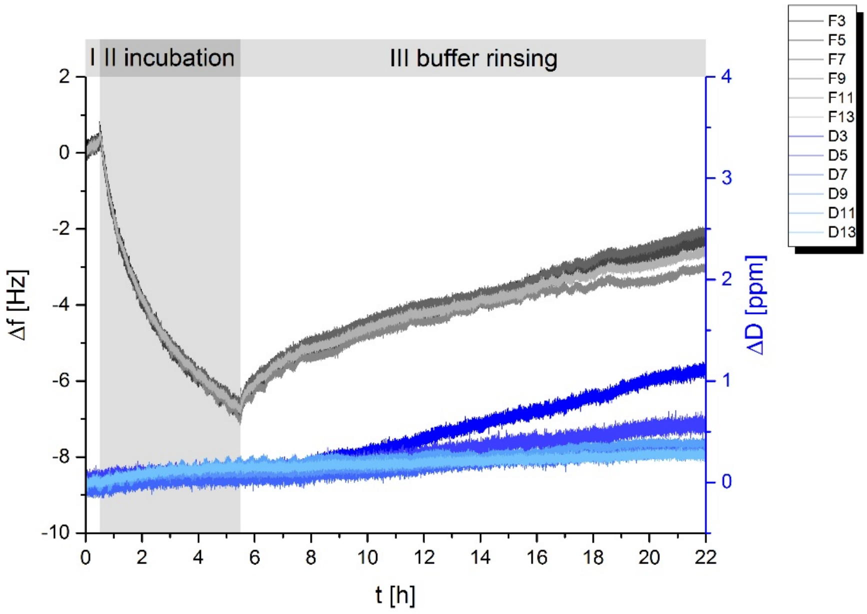

Figure S34. QCM-D adsorption and desorption kinetics of *Ab*PPO4 surface modification on Al_2O_3.

I. citrate buffer solution (0.8 mM, pH 6.8)
II. *Ab*PPO4 tyrosinase solution (2.3 U/mL)
III. citrate buffer solution (0.8 mM, pH 6.8)

5.14 QCM-D experiments on fluoropolymer surface

All measurements were performed like described in 5.13 using fluoropolymer coated quartz sensors (QSX 331 AF 1600, Biolin Scientific, Sweden).

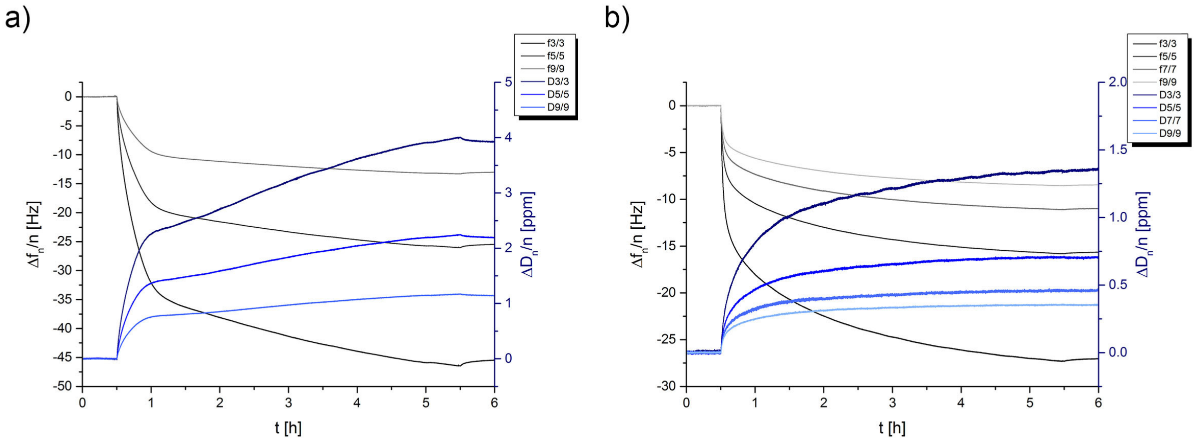

Figure S35. QCM-D response for $\Delta f_n/n$ (black) and $\Delta D_n/n$ (blue) for polyU$_1^C$ (a) and polyU$_2^C$ (b) on fluoropolymer coated sensors.

Figure S35 shows that by dividing frequency and energy dissipation by their corresponding overtones a frequency dependence is visible due to viscoelastic properties of the films. For rigid films with no frequency dependence the measurements would result in identical curves.

5.14.1 polyU$_1^C$ coating

Unimer polyU$_1^C$ was polymerized for 1 h according to protocol given in 3.4 with 0.75 µmol/mL substrate concentration and an *Ab*PPO4 tyrosinase concentration of 50 U/mL. Subsequently, the sample of 1.5 mL was diluted to 33 mL with degassed Milli-Q water and the sensor was incubated for 5 h followed by buffer rinsing. The resulted polyU$_1^C$ showed strong adsorption towards the fluoropolymer surface. In the beginning of the buffer rinsing step a minor fraction of adsorbed substrate was removed, afterwards Δf remained constant, proving the stability of the polymer coating.

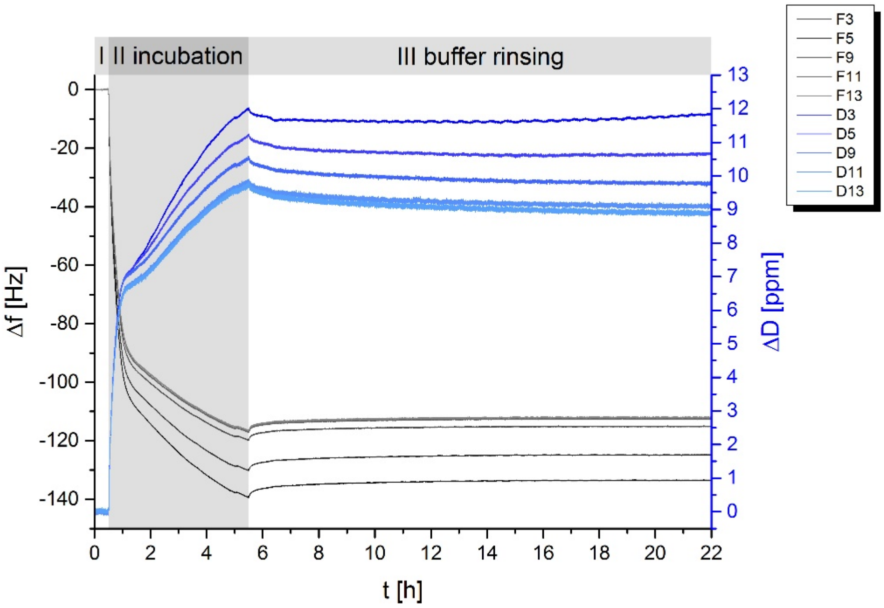

Figure S36. QCM-D adsorption and desorption kinetics of polyU$_1^C$ surface modification on fluoropolymer.

I. citrate buffer solution (0.8 mM, pH 6.8)
II. polyU$_1^C$ solution (34.1 nmol/mL)
III. citrate buffer solution (0.8 mM, pH 6.8)

5.14.2 Rinsing of polyU$_1$^{C} coating

To test for coating stability, the polyU$_1$^{C} coated sensor was rinsed with 599 mM NaCl solution and with hypersaline solution (4.2 M) for 1 h. After NaCl rinsing the equilibration of the buffer system takes longer (up to 2 h) indicating structural change of the coating. This is supported by the increase of energy dissipation and a higher frequency dependence pointing towards the formation of a more viscoelastic coating after rinsing. Because more water can couple to a less densely packed polymer with higher viscoelasticity, the calculated adsorbed mass of the polyU$_1$^{C} coating is higher after the NaCl rinsing than before. Nonetheless, since Δf is proportional to the adsorbed mass, by comparison of F3 (third overtone of Δf) before and after NaCl rinsing, a difference of 9.4 Hz (7.1%) can be assigned. The same effect is observed for the hypersaline solution rinsing step, leading to a higher calculated adsorbed mass after rinsing with hypersaline solution than before. The difference in F3 before and after rinsing amounts to -0.6 Hz (0.5%) compared to the initial coating leading to practically the same value after rinsing, which indicates a stable coating.

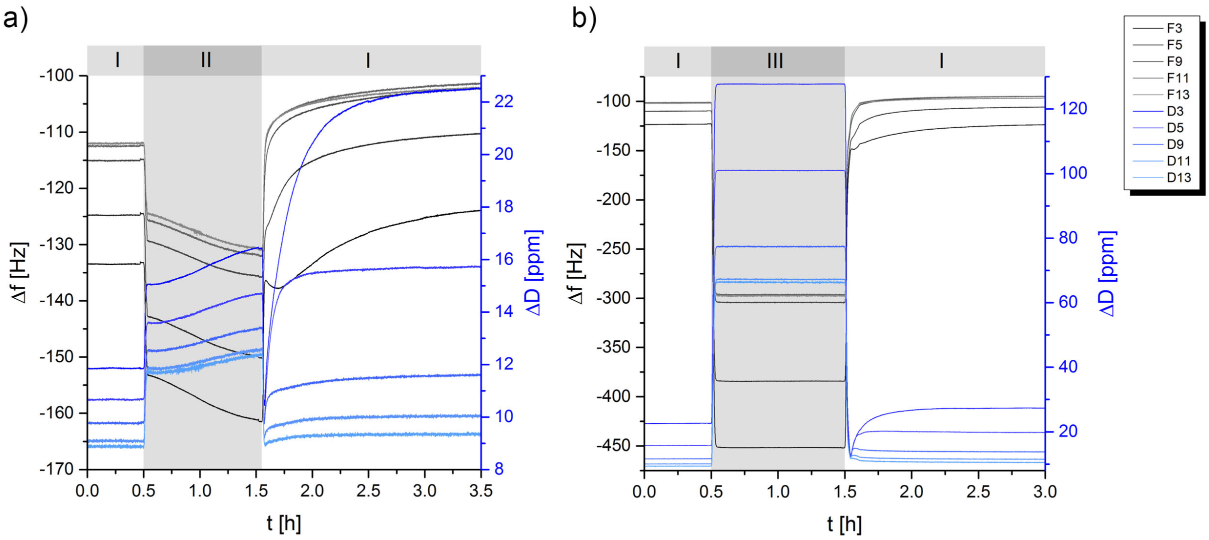

Figure S37. QCM-D adsorption and desorption kinetics of rinsing polyU$_1$^{C} modified fluoropolymer surface with 599 mM NaCl solution (a) and 4.2 M hypersaline solution (b).

I. citrate buffer solution (0.8 mM, pH 6.8)
II. NaCl solution (599 mM)
III. hypersaline solution (4.2 M)

5.14.3 polyU$_2^C$ coating

Unimer polyU$_2^C$ was polymerized for 1 h according to protocol given in 3.4 with 0.75 µmol/mL substrate concentration and an *Ab*PPO4 tyrosinase concentration of 50 U/mL. Subsequently, the sample of 1.5 mL was diluted to 33 mL with degassed Milli-Q water and the sensor was incubated for 5 h followed by buffer rinsing. The resulted polyU$_2^C$ showed strong adsorption towards the fluororpolymer surface. During buffer rinsing step Δf remained constant, proving the stability of the polymer coating.

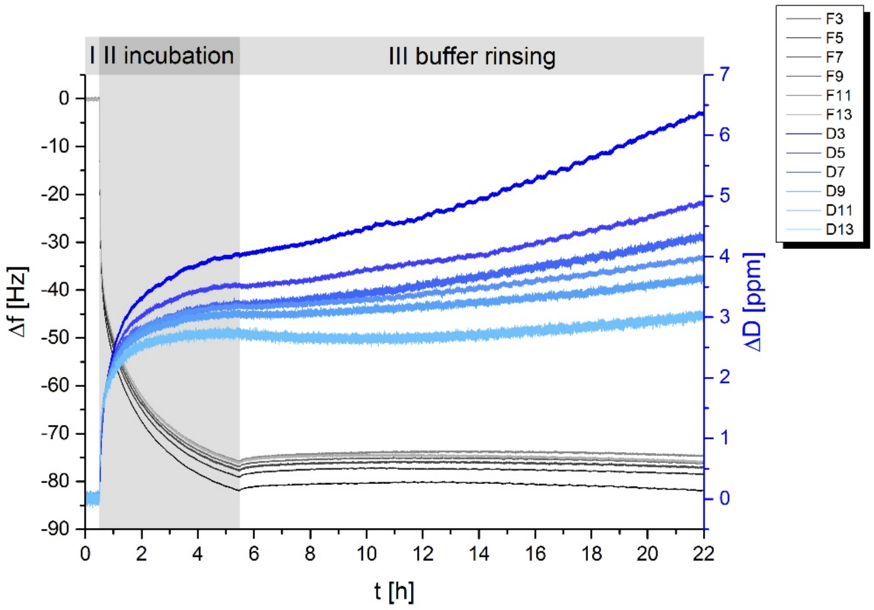

Figure S38. QCM-D adsorption and desorption kinetics of polyU$_2^C$ surface modification on fluoropolymer.

I. citrate buffer solution (0.8 mM, pH 6.8)
II. polyU$_2^C$ solution (34.1 nmol/mL)
III. citrate buffer solution (0.8 mM, pH 6.8)

5.14.4 Rinsing of polyU$_2^C$ coating

Coating stability of the polyU$_2^C$ coated sensor was tested by rinsing with 599 mM NaCl and with hypersaline solution (4.2 M) for 1 h. The calculated difference in adsorbed masses amounts to 19.2 ng/cm² (0.9%) for NaCl and to 88.8 ng/cm² (4.4%) for hypersaline solution compared to the initial coating, which demonstrates high resistance to salinity.

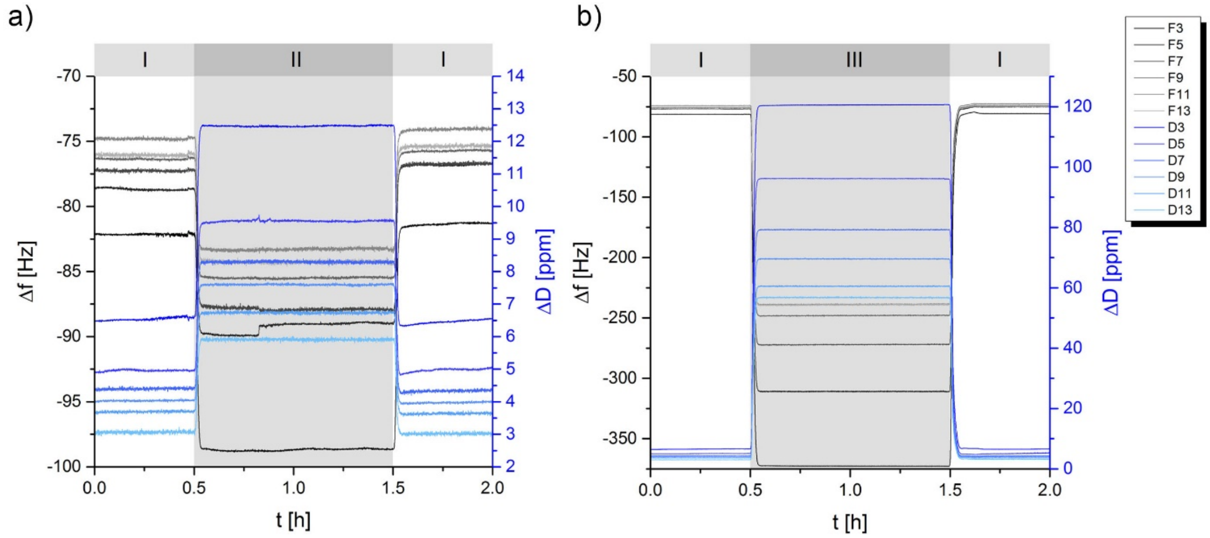

Figure S39. QCM-D adsorption and desorption kinetics of rinsing polyU$_2^C$ modified fluoropolymer surface with 599 mM NaCl solution (a) and 4.2 M hypersaline solution (b).

I. citrate buffer solution (0.8 mM, pH 6.8)
II. NaCl solution (599 mM)
III. hypersaline solution (4.2 M)

5.14.5 Unimer U_1^C control

As a control experiment QCM-D measurement of the pure unimer was carried out. Therefore, a solution of U_1^C (0.75 µmol/mL) was prepared, the sample of 1.5 mL was diluted to 33 mL with degassed Milli-Q water and the sensor was incubated for 5 h followed by buffer rinsing. The observed adsorption is rather weak, but Δf remained constant during buffer rinsing (Figure S40a), not washing off the formed coating. Also rinsing with 599 mM NaCl solution (Figure S40b) did not significantly remove the polymer film.

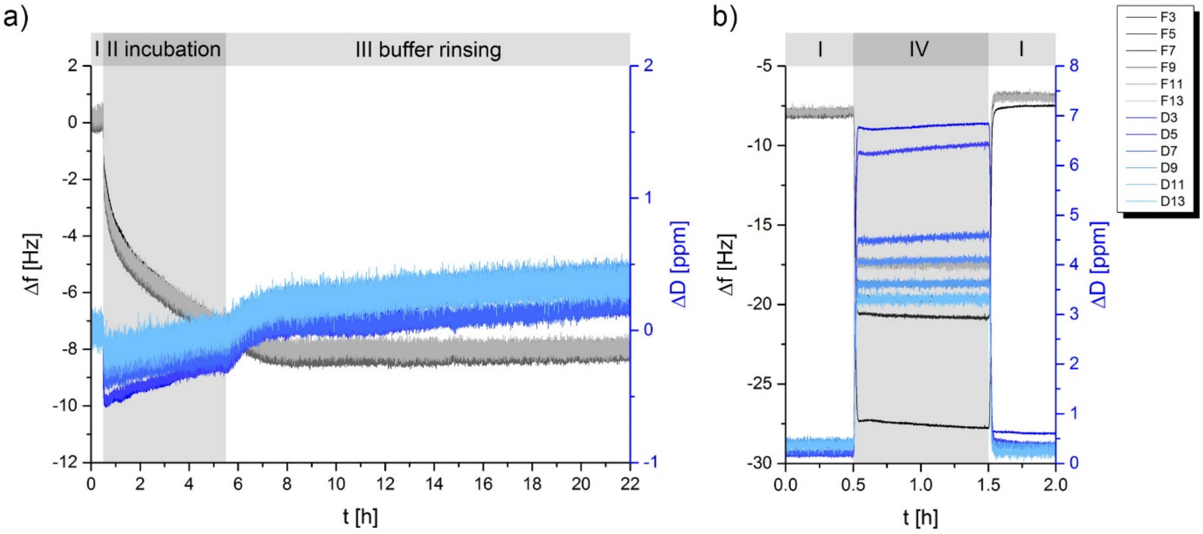

Figure S40. QCM-D adsorption and desorption kinetics of U_1^C surface modification on fluoropolymer. a) Incubation of U_1^C and buffer rinsing. b) Rinsing of U_1^C modified fluoropolymer surface with NaCl solution (599 mM).

I. citrate buffer solution (0.8 mM, pH 6.8)
II. unimer U_1^C solution (34.1 nmol/mL)
III. citrate buffer solution (0.8 mM, pH 6.8)
IV. NaCl solution (599 mM)

5.14.6 Unimer U_2^C control

QCM-D measurement of the pure unimer was carried out as a control experiment. A solution of U_2^C (0.75 µmol/mL) was prepared, the sample of 1.5 mL was diluted to 33 mL with degassed Milli-Q water and the sensor was incubated for 5 h followed by buffer rinsing. Negligible adsorption was observed for the unimer (Figure S41a). Rinsing the U_2^C coated fluoropolymer sensor with NaCl solution (599 mM) caused a slow equilibration, reaching almost the same Δf values after 6.5 h (Figure S41b).

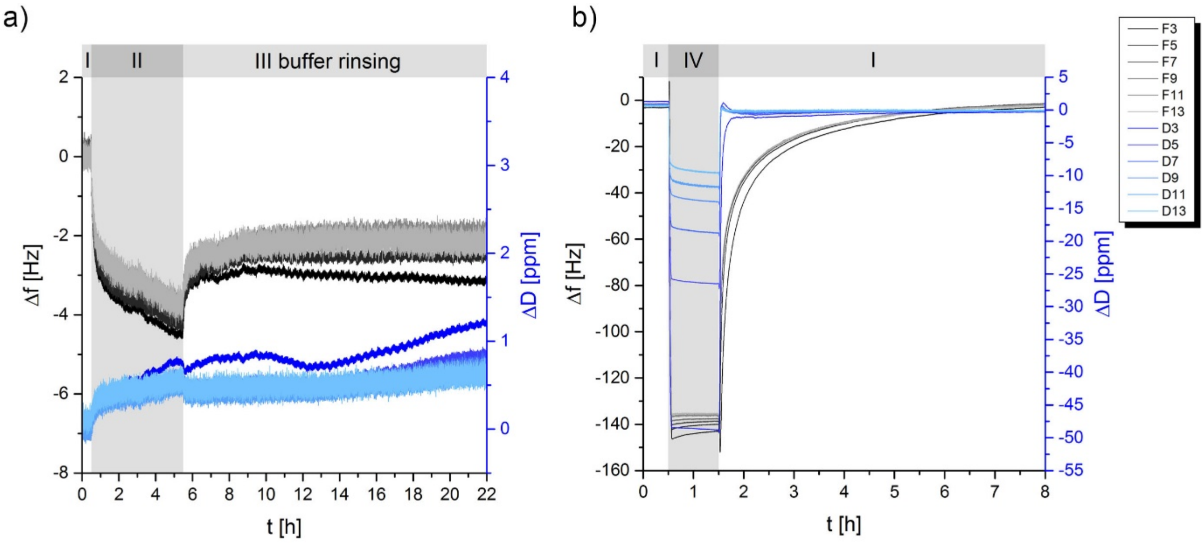

Figure S41. QCM-D adsorption and desorption kinetics of U_2^C surface modification on fluoropolymer. a) Incubation of U_2^C and buffer rinsing. b) Rinsing of U_2^C modified fluoropolymer surface with NaCl solution (599 mM).

I. citrate buffer solution (0.8 mM, pH 6.8)
II. unimer U_2^C solution (34.1 nmol/mL)
III. citrate buffer solution (0.8 mM, pH 6.8)
IV. NaCl solution (599 mM)

5.14.7 *Ab*PPO4 tyrosinase control

As a control experiment, QCM-D measurement of pure *Ab*PPO4 was performed. Therefore, a solution of *Ab*PPO4 tyrosinase (50 U/mL) was prepared, that contained the same enzyme concentration as in the polymerization mixture. The sample of 1.5 mL was diluted to 33 mL with degassed Milli-Q water and the sensor was incubated for 5 h followed by buffer rinsing. No adsorption of the enzyme was observed.

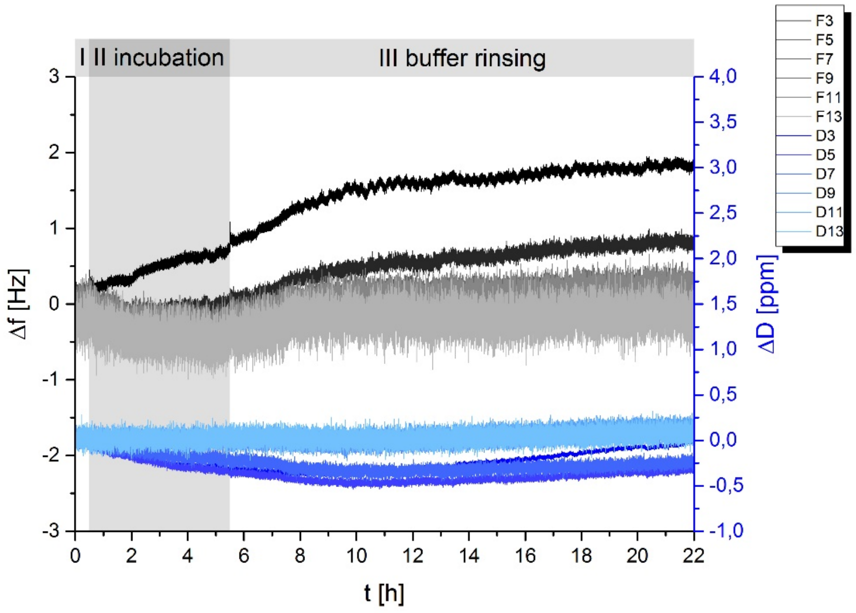

Figure S42. QCM-D adsorption and desorption kinetics of *Ab*PPO4 surface modification on fluoropolymer.

I. citrate buffer solution (0.8 mM, pH 6.8)
II. *Ab*PPO4 tyrosinase solution (2.3 U/mL)
III. citrate buffer solution (0.8 mM, pH 6.8)

5.15 Comparison of QCM-D kinetics and areal mass density

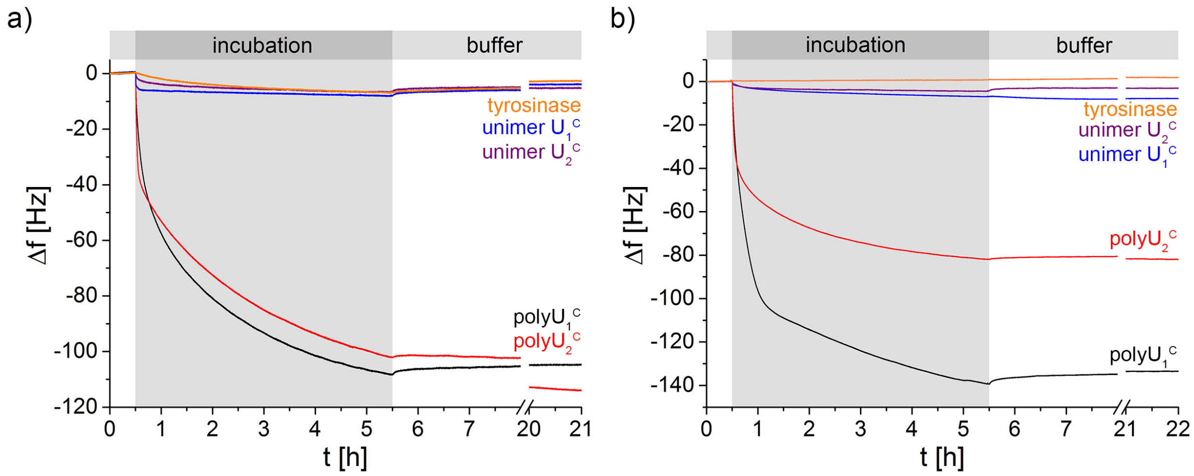

Figure S43. Comparison of QCM-D adsorption and desorption kinetics of polymers and unimer/enzyme references on Al_2O_3 coated sensors a) and fluoropolymer coated sensors b).

By using the Sauerbrey-model[13] for rigid coatings or the Voight-model[14] for viscoelastic coatings to approximate the kinetic QCM-D data, different areal mass densities (Δm) are obtained. The values from Table S4 show that films possess a certain viscoelasticity, since calculations from both models should be comparable for rigid films. Higher deviations are observed for $polyU_1^C$ films. The formed coatings of $polyU_2^C$ show lower deviation and are therefore more rigid, which can be explained by the branched topography and higher cross-linking tendency that results in a more dense coating while on the other hand a higher amount of coupled water molecules between the layers of $polyU_1^C$ contributes to the increased areal mass density.

Table S4. Comparison of areal mass densities obtained by different models for QCM-D data.

Coating	Δm Sauerbrey (ng/cm²)	Δm Voight (ng/cm²)	Deviation (%)
$polyU_1^C$ @ alox (pH 6.8)	1730 ± 110	4020 ± 480	132
$polyU_2^C$ @ alox (pH 6.8)	1580 ± 140	2950 ± 350	87
$polyU_1^C$ @ fluoropolymer (pH 6.8)	2260 ± 140	3090 ± 250	37
$polyU_2^C$ @ fluoropolymer (pH 6.8)	1380 ± 30	1750 ± 120	27

5.16 Colloidal Probe Atomic Force Microscopy to Characterize Adhesion Properties

5.16.1 Determination of the work of adhesion per unit area

a)

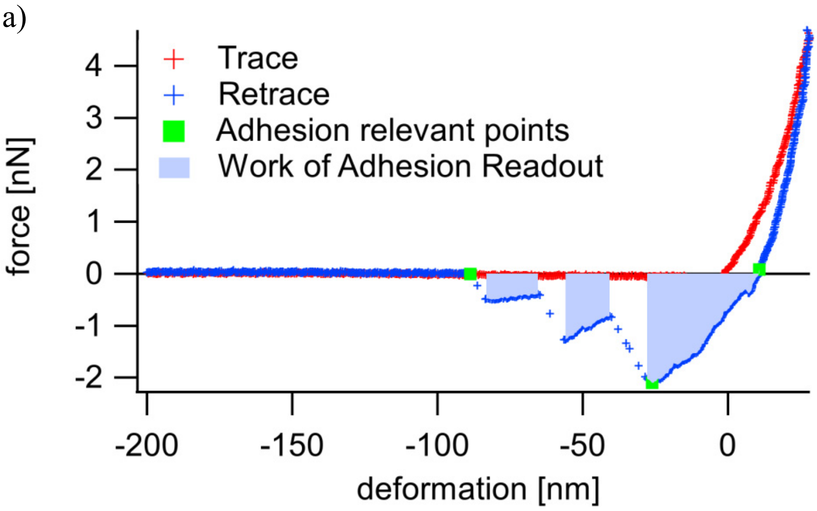

b)

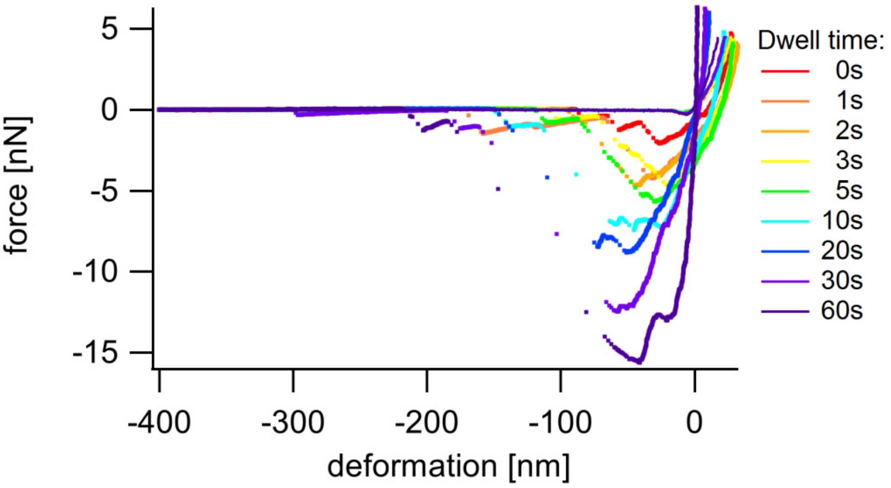

Figure S44. a) Typical force deformation measurement with colloidal probe on the example of polyU$_1^C$ coating for 2 h with zero dwell time on the surface. Red markers refer to the approach and the blue ones to the retraction direction, respectively. Green squares show adhesion relevant points from right to left: first, zero force while retracting at which point adhesion forces are dominating; second, maximum restoring force equaling the force of adhesion; third, jump out of contact. Blue shaded areas represent the part of the measurement at which the colloidal probe is in stable adhesive contact with the coating. b) Series of force deformation measurements recorded continuously with varying dwell times in contact between 0 and 60 s.

Quantification of adhesive interaction properties of two surfaces with colloidal probe is done by analyzing its detachment process, exemplarily depicted by the blue parts in Figure S44a. Two quantities are usually determined: i) The maximum restoring force of the cantilever (lowest green square) is commonly referred to as force of adhesion. ii) The total work needed

to pull the two surfaces apart is indicated by the shaded blue area in Figure S44a. However, it is not directly possible to compare results from different experiments by either their adhesion force or work of adhesion values. It is because both are dependent on other parameters, especially geometry of probe and sample, load force and indentation depth, all affecting the contact area over which adhesion is acting. To account for these influences one approach is to normalize the work of adhesion by the contact area. With regard to colloidal probe AFM, it is very difficult to measure contact areas independently in the range of some tens or hundreds nm^2, which is the order of magnitude for our experiments. To overcome this issue, well-established contact mechanics theories like the Johnson-Kendall-Roberts model (JKR)[15], the Derjaguin-Muller-Toporov model (DMT)[16], or the Maugis Dugdale model (MD)[17] can be used. In the JKR and DMT model the work of adhesion per unit area γ is given by:

$$\gamma_{JKR} = \frac{2F_A}{3\pi R_{eff}}; \gamma_{DMT} = \frac{F_A}{2\pi R_{eff}}; \frac{1}{R_{eff}} = \frac{1}{R_{probe}} + \frac{1}{R_{sample}} \quad \text{(eq. 5.17.1)}$$

Both theories are based among others on the assumption of mechanical contact between two isotropic elastic bodies but differ crucially on the way adhesion is contributing. In JKR the adhesion is exclusively acting within the contact area while contrarily in DMT adhesion forces are acting outside but close to the contact zone. Maugis[17] discovered that both models can be unified to a universal model with JKR and DMT being the limiting cases but also describing all contact scenarios in between. One practical issue with the Maugis model is that it is a purely analytical model which makes fitting or describing experimental data a very complex task. Therefore, we used the approximation method of the Maugis model developed by Carpick, Ogletree and Salmeron.[18]

In general, the dependency of the contact radius a, the measured load force L, and the adhesion force L_C is given by:

$$a = \left(\frac{\alpha+\sqrt{1-L/L_C}}{1+\alpha}\right)^{2/3} \cdot a_0 \quad \text{(eq. 5.17.2)}$$

Here a_0 is the contact radius at zero load force and α is the JKR ($\alpha = 1$) - DMT ($\alpha = 0$) transition parameter. To fit this equation to the experimental force-deformation curves during retraction the contact radius is required. We calculated a by geometrical consideration of a sphere pressing on a plain film and deforming it by δ according to:

$$a = \left({R_{probe}}^2 - \left(R_{probe} - \delta\right)^2\right)^{0.5} \quad \text{(eq. 5.17.3)}$$

Since there is no reference point for absolute values of the contact radius available in measurements, we set the contact radius of the measurement $a(L{=}L_C) = 0$, which equals the theoretical lower limit for DMT $a(L{=}L_C)_{\alpha=0} = ((0+0)/1)^{2/3}\, a_0 = 0$. A finite contact area at break off is introduced by expansion of eq. 5.17.2 by a general offset $a(L{=}L_C)$ so the fit function becomes:

$$a = \left(\frac{\alpha+\sqrt{1-L/L_C}}{1+\alpha}\right)^{2/3} \cdot a_0 - \left(\frac{\alpha}{1+\alpha}\right)^{2/3} \cdot a_0 \qquad \text{(eq. 5.17.4)}$$

One example of the described method is presented in Figure S45 for a measurement on polyU$_1^C$.

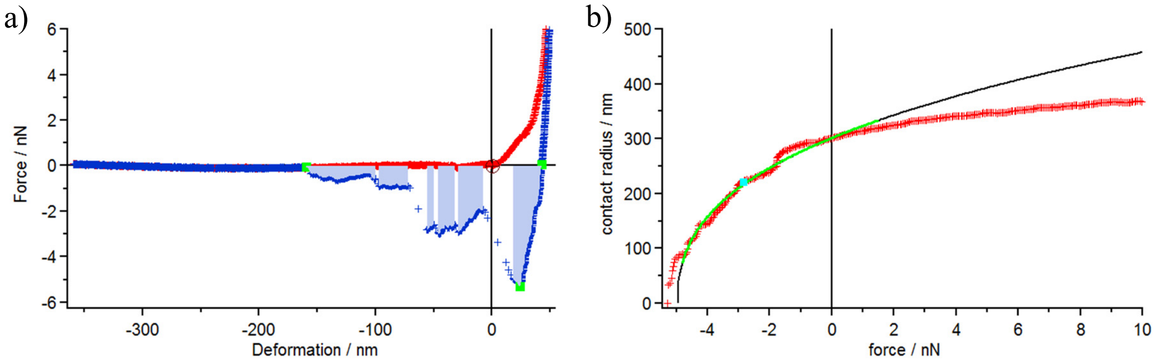

Figure S45. a) Force-deformation curve for poly U$_1^C$ similarly displayed as Figure S44a. b) Calculated contact radius from measurement in dependency of the measured load force (red). The fitted range according to eq. 5.17.4 is displayed in green and the black line represents eq. 5.17.4 over the full force range.

The fit in Figure S45b reveals a force of adhesion $L_{C,fit}$ of 5.0 nN which is in good agreement with the measured value of 5.2 nN. Using the approximation described by Carpick *et al.*[18] α can be transferred to the Maugis parameter and $\gamma_{MD} = 0.33$ mJ m^{-2} is determined, compared to $\gamma_{JKR} = 0.46$ mJ m^{-2} and $\gamma_{DMT} = 0.34$ mJ m^{-2} according to eq. 5.17.1.Since we cannot tell *a priori* if JKR or DMT is applicable and the Maugis approximation offers more versatility we determined γ by the technique described here.[8]

In Figure S46 a statistical overview for both polymers, the two coating times, and the additional treated samples is shown in a box plot fashion. All box-plots presented are according to the following criteria: whiskers end at 10% and 90%, boxes enclose the values between 25% and 75% and the mean value is represented as the line within the box.

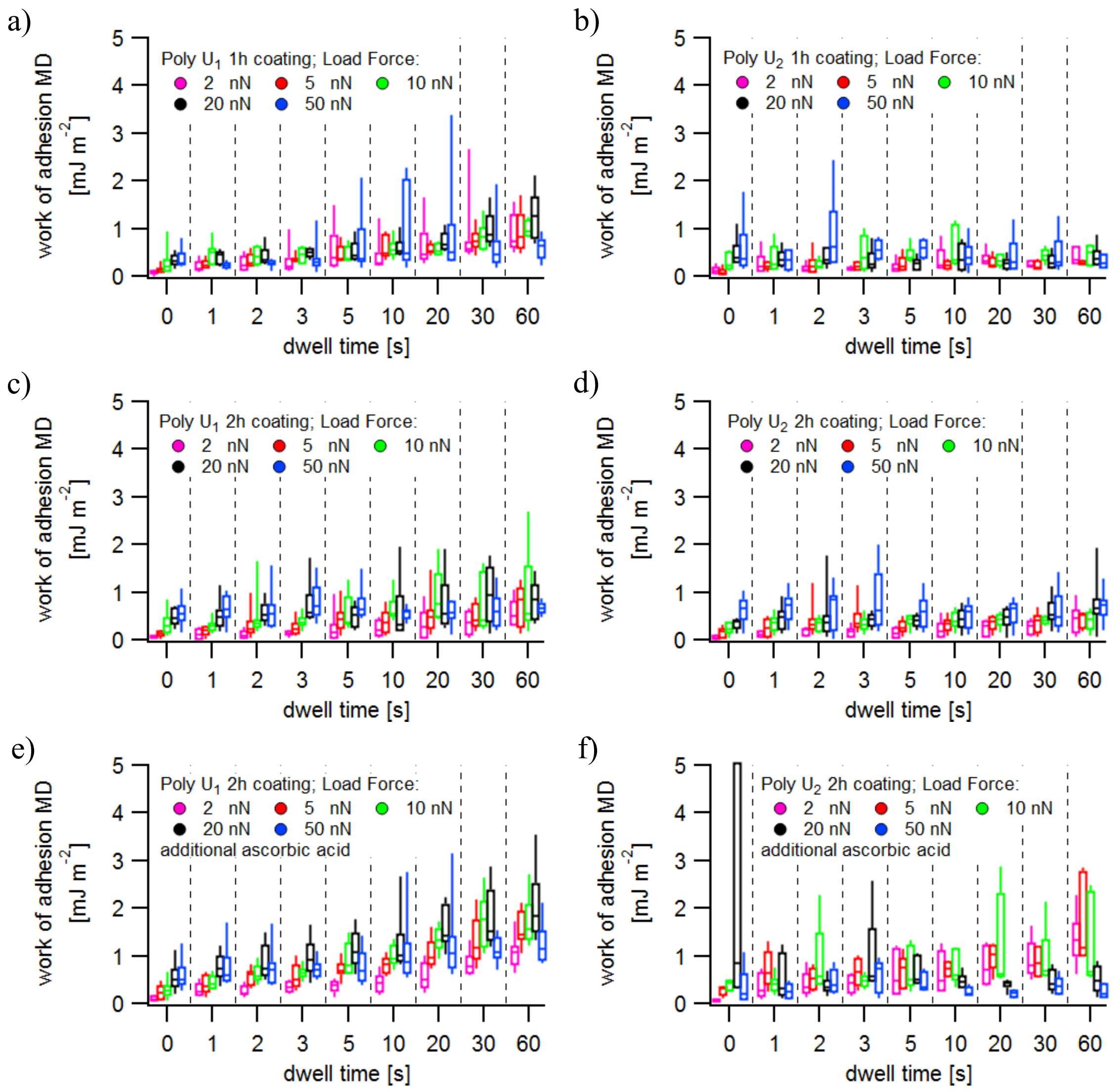

Figure S46. Statistical evaluation of the work of adhesion per unit area from the Maugis approximation. a) each box n = 9, b) each box n = 6, c) each box n = 9, d) each box n = 15. e) each box n = 27, f) each box n = 6. For all six different samples colloidal probe experiments were conducted with load forces between 2 nN and 50 nN and dwell times between 0 s and 60 s.

For almost every sample and load force we find an increase of adhesion with dwell time, with varying magnitude. While increasing the load force from 2 nN up to 10 – 20 nN we find a slight increase in adhesion. Depending on the polymer coating at load forces between 10 to 50 nN a sudden drop in the work of adhesion per unit area is observed. This is probably due to an indentation of the colloidal probe through the polymer coating, which finally presses against the underlying silicon wafer. This was observed in the loading part of these force deformation curves with a maximum deformation close to the film thickness, found in AFM images, and reaching the constant compliance regime.

5.16.2 Determination of the work of adhesion per unit area for sequential adhesives failures

While the above described calculation method of γ allows for characterizing the adhesion properties for the first jump out of contact, it is not taking into account additional rip-off events observable in Figure S45 and S44a. We attribute these additional rip-off events to an initially incomplete adhesive failure between silica probe and polymer layer. At higher separations longer polymer chains or parts of the polymer layer are still adhesively connected to the probe and contribute to further adhesive interactions, similar to the stepwise opening of a Velcro fastener. Conclusively, these interactions should be taken into account since for adhesive applications the total energy needed to fully separate two surfaces is required. Therefore, we use the established theory of the Maugis approximation presented above and advanced it further to include these events.

Generally, γ is defined by the ratio of the performed work W needed to separate two surfaces to the normalization contact area A. W is directly accessible from the AFM measurements by the area enclosed by force-deformation curve and the x-axis at negative forces (see blue shaded regions in Figure S45a. In case of sequential rip-off events the total work W_{rip} equals the sum of all performed work in stable adhesive contact between probe and sample. Note here, that the areas of instable contacts, indicated by interruptions between blue areas of Figure S45a, should be excluded since the AFM cantilever is not in equilibrium there.

The determination of the normalization contact area A however is not as straight forward. First, we transferred the contact radius versus force fit curve of eq. 5.17.4 to a force-deformation curve of the fit by solving eq. 5.17.3 for δ. (Figure S47)

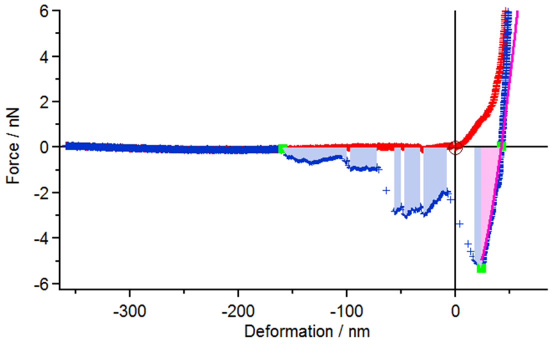

Figure S47. Force-deformation curve of Figure S45a with the Maugis fit as pink line and the calculated W_{fit} as shaded pink area.

Since we know γ_{MD} from the Maugis approximation and we can now calculate the performed work W_{fit} for the first adhesive failure, as described above, A_{fit} here is given by:

$$A_{fit} = \frac{W_{fit}}{\gamma_{MD}} \qquad \text{(eq. 5.17.5)}$$

In this situation all adhesive interaction sites are contributing to the measured restoring force acting over the contact area A_{fit} . To allow for comparability of our results to literature values, where typically only one break off is occurring, we determine the work of adhesion per unit area for sequential failure events according to:

$$\gamma_{rip} = \frac{W_{rip}}{A_{fit}} \qquad \text{(eq. 5.17.6)}$$

Thereby we are emulating a single adhesive failure event until which all the measured adhesive work W_{rip} is performed while pulling the two involved surfaces apart.
Figure S48 shows the statistical evaluation of the same force-deformation curve as in Figure S47 but evaluated including sequential adhesive failures as described above.

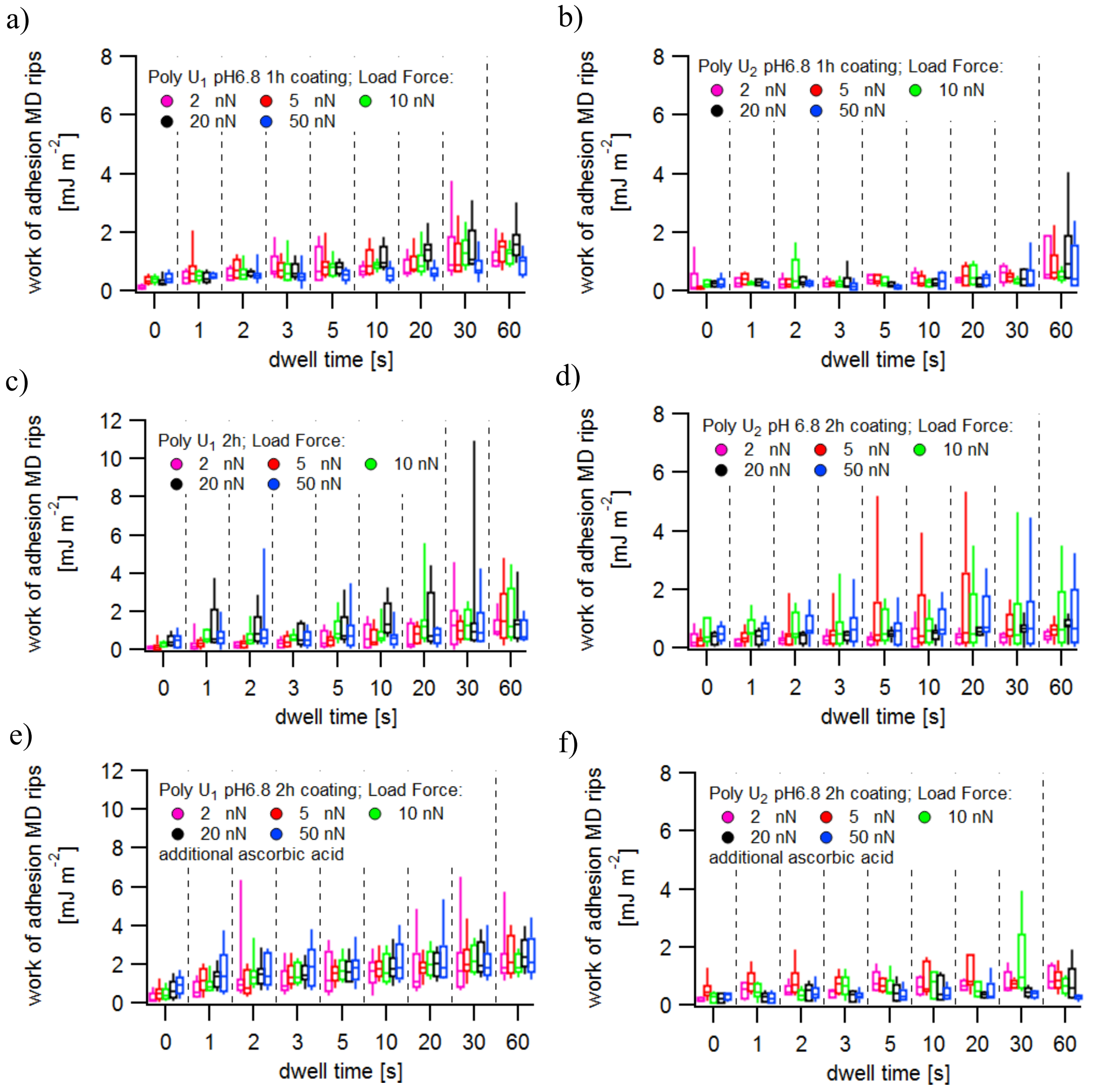

Figure S48. Statistical evaluation of the work of adhesion per unit area from the Maugis approximation including sequential adhesive failure events. a) each box n = 9, b) each box n = 6, c) each box n = 9, d) each box n = 15. e) each box n = 27, f) each box n = 6. For all six different samples colloidal probe experiments were conducted with load forces between 2 nN and 50 nN and dwell times between 0 s and 60 s.

The observed trends for the dependency of γ_{rip} with varying dwell time are similar to those found in Figure S46 however the absolute values for the adhesion increase up to one order of magnitude while additionally the scattering increases.

5.16.3 Comparison of work of adhesion

For ease of comparison of the above obtained values for work of adhesion, average values for the different coating experiments only with 2 nN load force and the different polymer coatings were extracted and are shown in Figure S49. The passivated silicon wafers were incubated with polyU$_1^C$ and polyU$_2^C$ for 1 h (blue circles), for 2 h (green diamonds) and for 1 h with additional post-treatment with sodium ascorbate for another hour (red triangles).

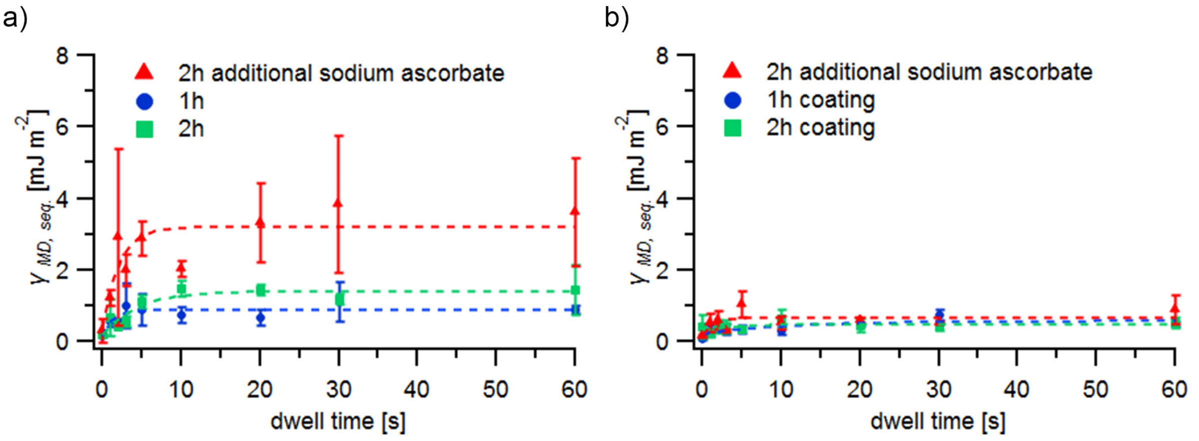

Figure S49. Work of adhesion per unit area $\gamma_{MD,seq.}$ of polyU$_1^C$ coating (a) and polyU$_2^C$ coating (b) in dependence of dwell time of the probe. Constant load force of 2 nN; lines are to guide the eye only. Every point represents the mean of 3 measurements, except polyU$_1^C$ red triangles n = 6; error bars represent s.e.m.

5.16.4 Reference experiment with Cell-Tak™

As a reference measurement Cell-Tak™ coated silicon wafer substrates were used for adhesion testing with CP-AFM at pH 6.8. Cell-Tak™ is a tissue adhesive based on a extracted protein mixture from *Mytilus edulis*, including Mefp-1.[19] Coating solution of 1.5 mL with a protein concentration of 0.5 mg/mL was prepared by adding 100 µL Milli-Q water, 505.5 µL 1 M NaOH and 206,4 µL sodium citrate buffer (17 mM, pH 6.8) to 688.1 µL Cell-Tak™ solution (1.09 mg/mL in 5% acetic acid). After 1 h of substrate incubation 10 µL of sodium ascorbate solution (1.13 M) was added and coating was continued for 1 h until the substrate was rinsed with sodium citrate buffer (17 mM, pH 6.8). Storage and measurement was done in sodium citrate buffer solution (17 mM, pH 6.8).

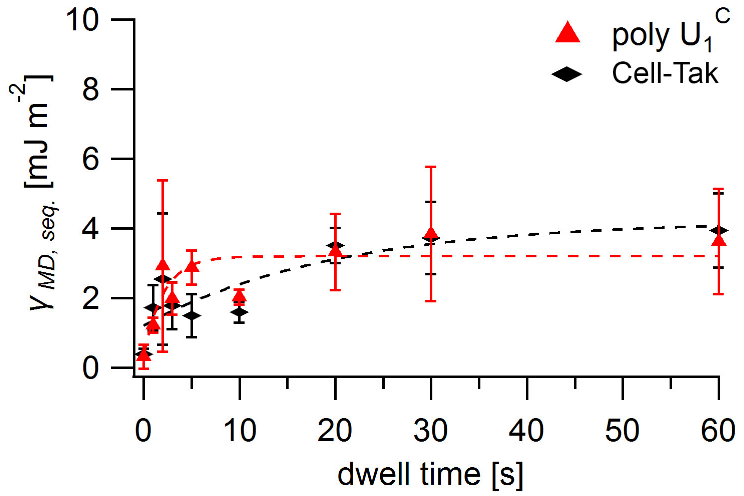

Figure S50. Comparison of the work of adhesion per unit area $\gamma_{MD,seq}$ of Cell-Tak™ (black diamonds) coated and polyU$_1^C$ (red triangles) coated silicon wafer substrates after identical coating conditions (1 h followed by addition of sodium ascorbate for another 1 h) at 2 nN load force and various dwell times. (pulling rate 2 µm/s, n = 3, error bars represent s.e.m, lines are to guide the eye only).

6. References

[1] M. Pretzler, A. Bijelic, A. Rompel, *Sci. Rep.* **2017**, *7*, 1810.

[2] J. B. W. Hammond, R. Nichols, *J. Gen. Microbiol.* **1976**, *93*, 309.

[3] F. W. Studier, *Protein Expres. Purif.* **2005**, *41*, 207.

[4] C. S. French, H. W. Milner, in *Methods in Enzymology, Vol. Volume 1*, Academic Press, **1955**, pp. 64.

[5] H. W. Duckworth, J. E. Coleman, *J. Biol. Chem.* **1970**, *245*, 1613.

[6] J. L. Hutter, J. Bechhoefer, *Rev. Sci. Instrum.* **1993**, *64*, 1868.

[7] H.-J. Butt, B. Cappella, M. Kappl, *Surf. Sci. Rep.* **2005**, *59*, 1.

[8] M. Seuss, A. Fery, *MaxSeuss/AFM-Force-Curve-Analyzer-IgorPro: AFM Force Curve Analyzer for IgorPRO (2018). doi:10.5281/zenodo.1208549.*

[9] R. Mirshafian, W. Wei, J. N. Israelachvili, J. H. Waite, *Biochemistry* **2016**, *55*, 743.

[10] S. Ito, G. Prota, *Experientia* **1977**, *33*, 1118.

[11] H. L. Bandey, A. Robert Hillman, M. J. Brown, S. J. Martin, *Faraday Discussions* **1997**, *107*, 105.

[12] I. STEINHORN, G. ASSAF, J. R. GAT, A. NISHRY, A. NISSENBAUM, M. STILLER, M. BEYTH, D. NEEV, R. GARBER, G. M. FRIEDMAN, W. WEISS, *Science* **1979**, *206*, 55.

[13] G. Sauerbrey, *Z. Phys.* **1959**, *155*, 206.

[14] M. V. Voinova, M. Rodahl, M. Jonson, B. Kasemo, *Phys. Scr.* **1999**, *59*, 391.

[15] K. L. Johnson, K. Kendall, A. D. Roberts, *P. Roy. Soc. A.-Math. Phy.* **1971**, *324*, 301.

[16] B. V. Derjaguin, V. M. Muller, Y. P. Toporov, *J. Colloid Interf. Sci.* **1975**, *53*, 314.

[17] D. Maugis, *J. Colloid Interf. Sci.* **1992**, *150*, 243.

[18] R. W. Carpick, D. F. Ogletree, M. Salmeron, *J. Colloid Interf. Sci.* **1999**, *211*, 395.

[19] D. S. Hwang, H. J. Yoo, J. H. Jun, W. K. Moon, H. J. Cha, *Appl. Environ. Microbiol.* **2004**, *70*, 3352.
